# Supplementary material for: Flexible experimental designs for valid single-cell RNA-sequencing experiments allowing batch effects correction
Source: Nat Commun. 2020 Jul 1;11:3274. doi: 10.1038/s41467-020-16905-2 (PMC7330047; doi:10.1038/s41467-020-16905-2)
Supplement: Supplementary file 1 — Supplementary Information [file 41467_2020_16905_MOESM1_ESM.pdf]

Flexible Experimental Designs for Valid Single-cell RNA-sequencing Experiments Allowing Batch Effects Correction

Fangda Song[1], Ga Ming Angus Chan[1], and Yingying Wei[1*]

[1] *Department of Statistics, The Chinese University of Hong Kong, Hong Kong SAR, China*
[*] *Correspondence should be addressed to YY.W. (yweicuhk@gmail.com)*

Supplementary Figures

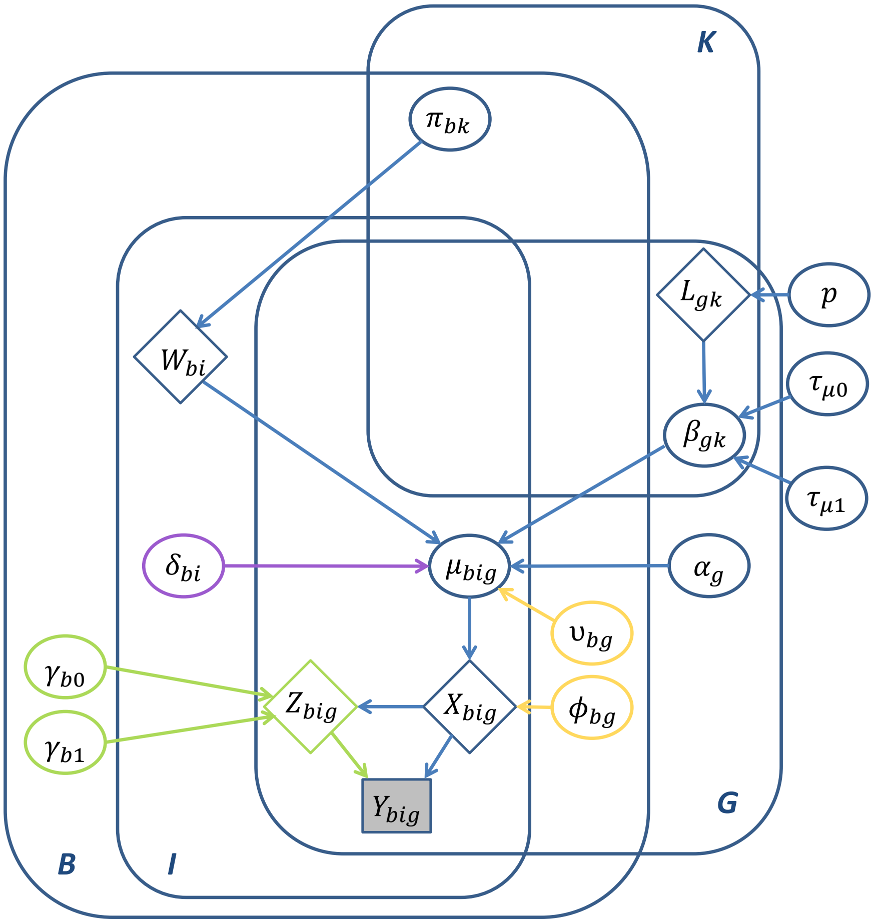

Supplementary Figure 1: The graphical representation of the BUSseq model. The yellow color corresponds to batch effects; the green color models the dropout events; the purple color indicates the cell-specific size factor. The ellipses are for parameters; the diamonds represent latent variables; and only Y_{big} in the grey rectangle is observed.

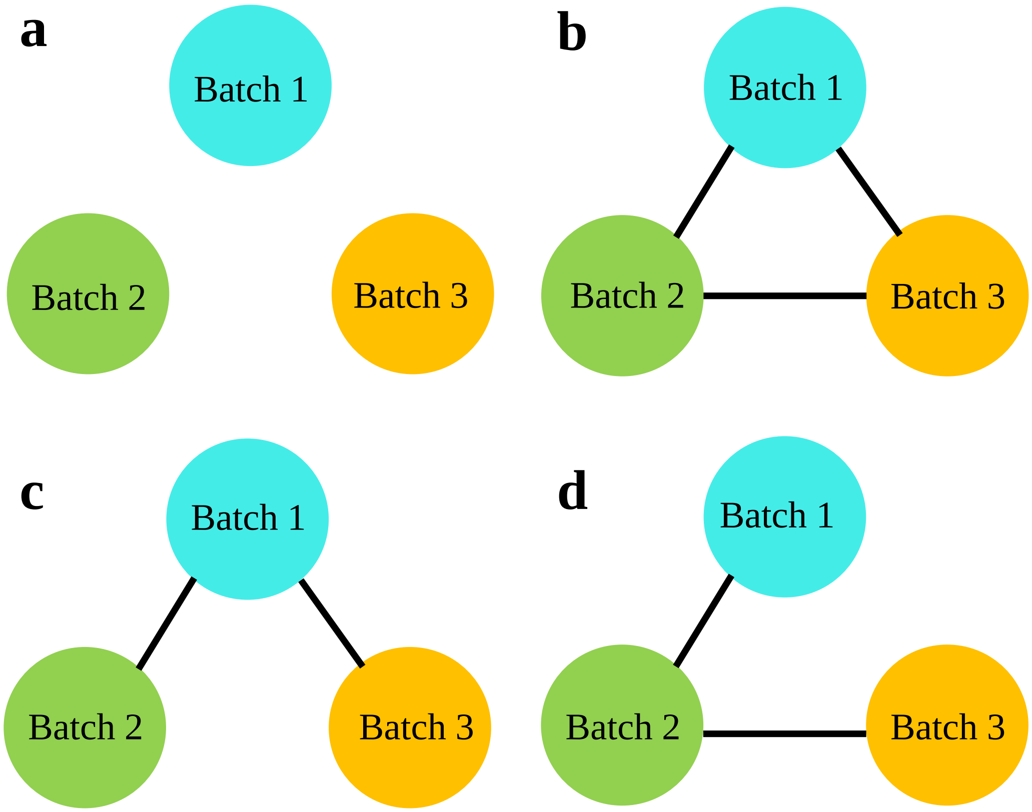

Supplementary Figure 2: The batch graphs for the experiment designs in **Figure 2**. **(a)** The confounded design. **(b)** The complete setting design. **(c)** The reference panel design. **(d)** The chain-type design.

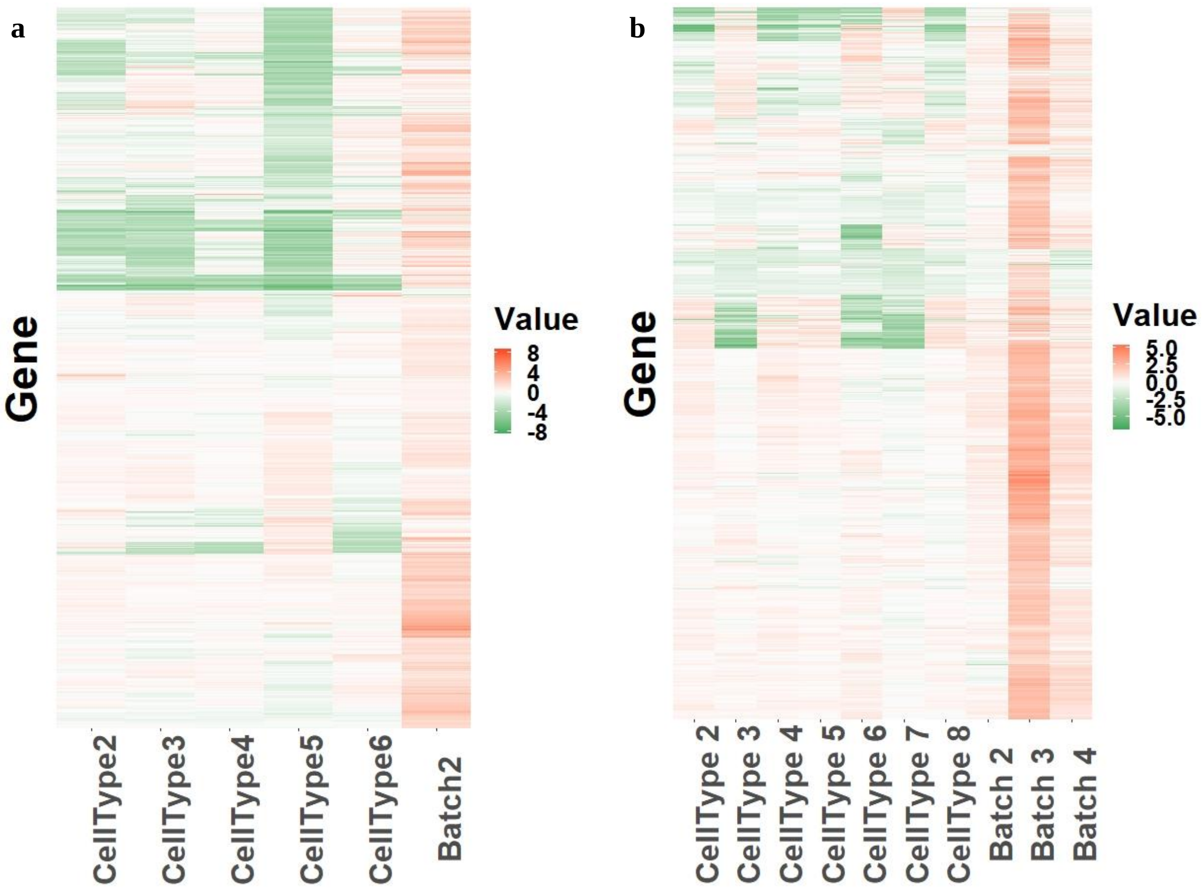

Supplementary Figure 3: Heatmap of the estimated cell type effects and batch effects by BUSseq in two real studies. Each row represents a gene, and each column corresponds to the cell type effect $\beta_{gk}, 2 \leq k \leq K$ or the batch effect $\nu_{bg}, 2 \leq b \leq B$. **(a)** Mouse Hematopoietic study. **(b)** Human Pancreas study.

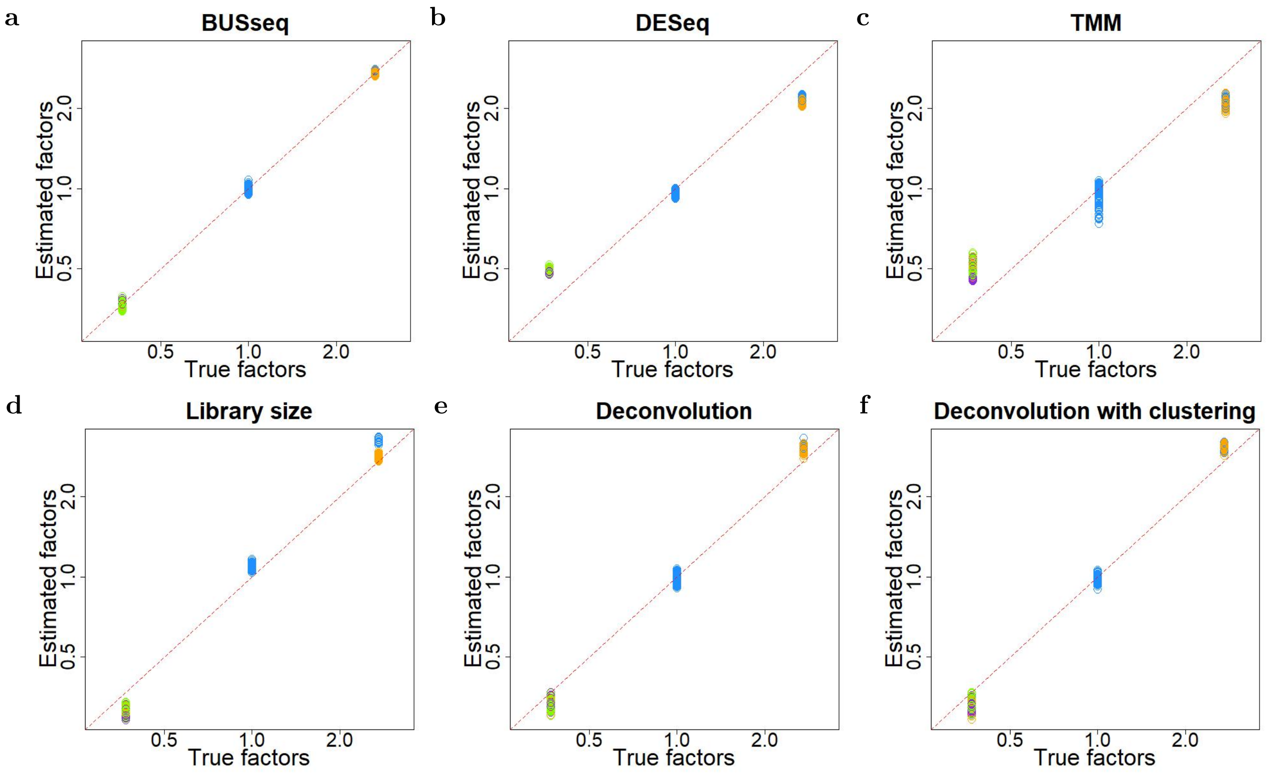

Supplementary Figure 4: Performance of benchmarked normalization methods with the simulation dataset. The size factor estimates for all cells are plotted against the true values for **(a)** BUSseq, **(b)** DESeq, **(c)** TMM, **(d)** library size normalization, **(e)** deconvolution and **(f)** deconvolution with clustering. The axes are in the log-scale. For comparison, the size factor estimates by each method are scaled such that the grand mean across cells was the same as that for the true values. The diagonal line means that the scaled estimate is equal to the true factors. The four cell types are colored by blue, orange, green and pink, respectively.

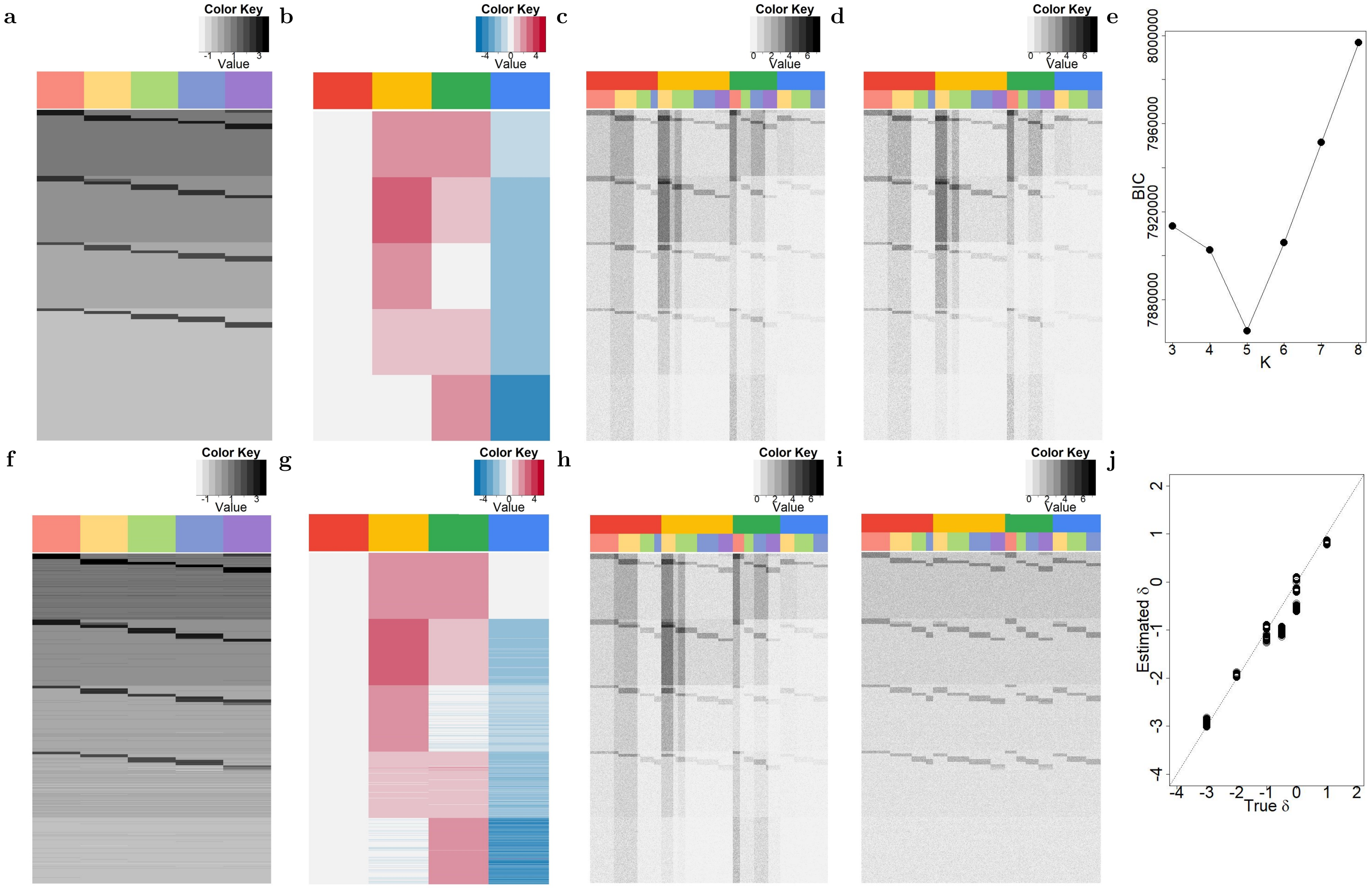

Supplementary Figure 5: Patterns of the simulation dataset in the sensitivity analysis with high zero rates. **(a)** True log-scale mean expression level for each cell type $\boldsymbol{\alpha}+\boldsymbol{\beta}$. Each row represents a gene, and each column corresponds to a cell type. **(b)** True batch effects. Each row represents a gene, and each column corresponds to a batch. **(c)** True underlying expression levels $\boldsymbol{X}$. Each row represents a gene, and each column corresponds to a cell. The upper colored bar indicates the batches, and the lower colored bar represents the cell type. **(d)** The simulated observed data $\boldsymbol{Y}$. **(e)** BIC plot for different numbers of cell types. **(f)** The estimated log-scale mean expression level for each cell type $\widehat{\boldsymbol{\alpha}}+\widehat{\boldsymbol{\beta}}$. **(g)** Estimated batch effects. **(h)** Imputed expression levels $\widehat{\boldsymbol{X}}$. **(k)** Corrected count data $\widetilde{\boldsymbol{X}}$ grouped by batches. **(l)** Scatter plot of the estimated versus true cell-specific size factors. BUSseq correctly recovers the parameters even if one batch has more than 80% zero counts.

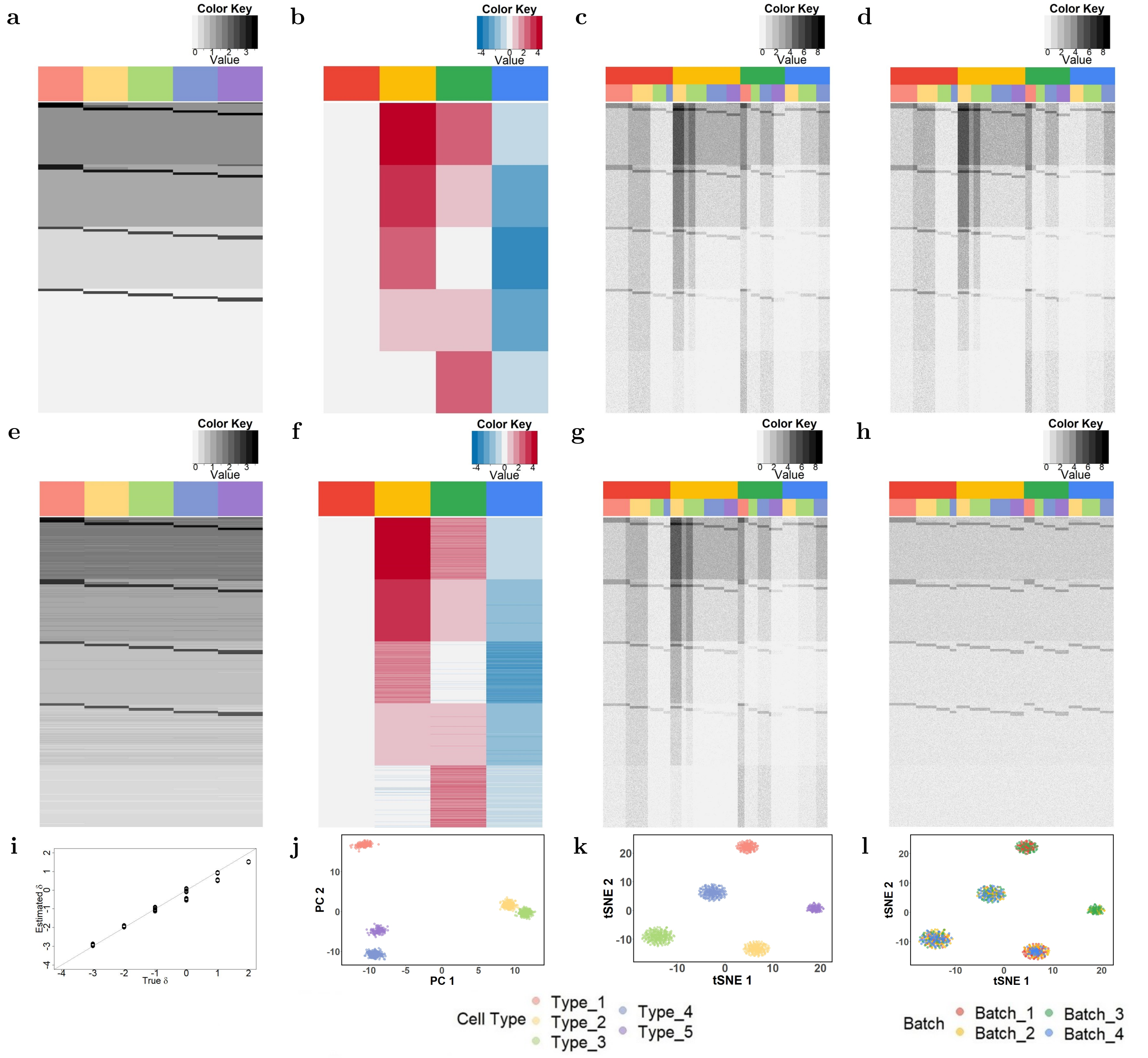

Supplementary Figure 6: Patterns of the simulation dataset in the sensitivity analysis which allows cell-type-specific overdispersion parameters. **(a)** True log-scale mean expression levels $\boldsymbol{\alpha} + \boldsymbol{\beta}$ for each cell type. Each row represents a gene, and each column corresponds to a cell type. **(b)** True batch effects $\boldsymbol{\nu}$. Each row represents a gene, and each column corresponds to a batch. **(c)** True underlying expression levels $\boldsymbol{X}$. Each row represents a gene, and each column corresponds to a cell. The upper colored bar indicates the batch of each cell, and the lower colored bar represents the type of each cell. **(d)** The simulated observed data $\boldsymbol{Y}$. **(e)** The estimated log-scale mean expression levels $\widehat{\boldsymbol{\alpha}} + \widehat{\boldsymbol{\beta}}$ for each cell type. **(f)** Estimated batch effects $\widehat{\boldsymbol{\nu}}$. **(g)** Imputed expression levels $\widehat{\boldsymbol{X}}$. **(h)** Corrected count data $\widetilde{\boldsymbol{X}}$ grouped by batch. **(i)** Scatter plot of the estimated cell-specific size factor versus the true cell-specific size factor. Finally, we draw **(j)** the principal component analysis (PCA) plot for each cell colored by cell type, **(k)** the t-distributed stochastic neighbor embedding (t-SNE) plot colored by cell type and **(l)** the t-SNE plot colored by batch indicators based on the corrected count data shown in **(h)**. BUSseq precisely recovers the true parameter values and perfectly clusters cells by cell type even if the overdispersion parameter is not only gene- and batch-specific but also cell type specific, with HEGs having lower overdispersion.

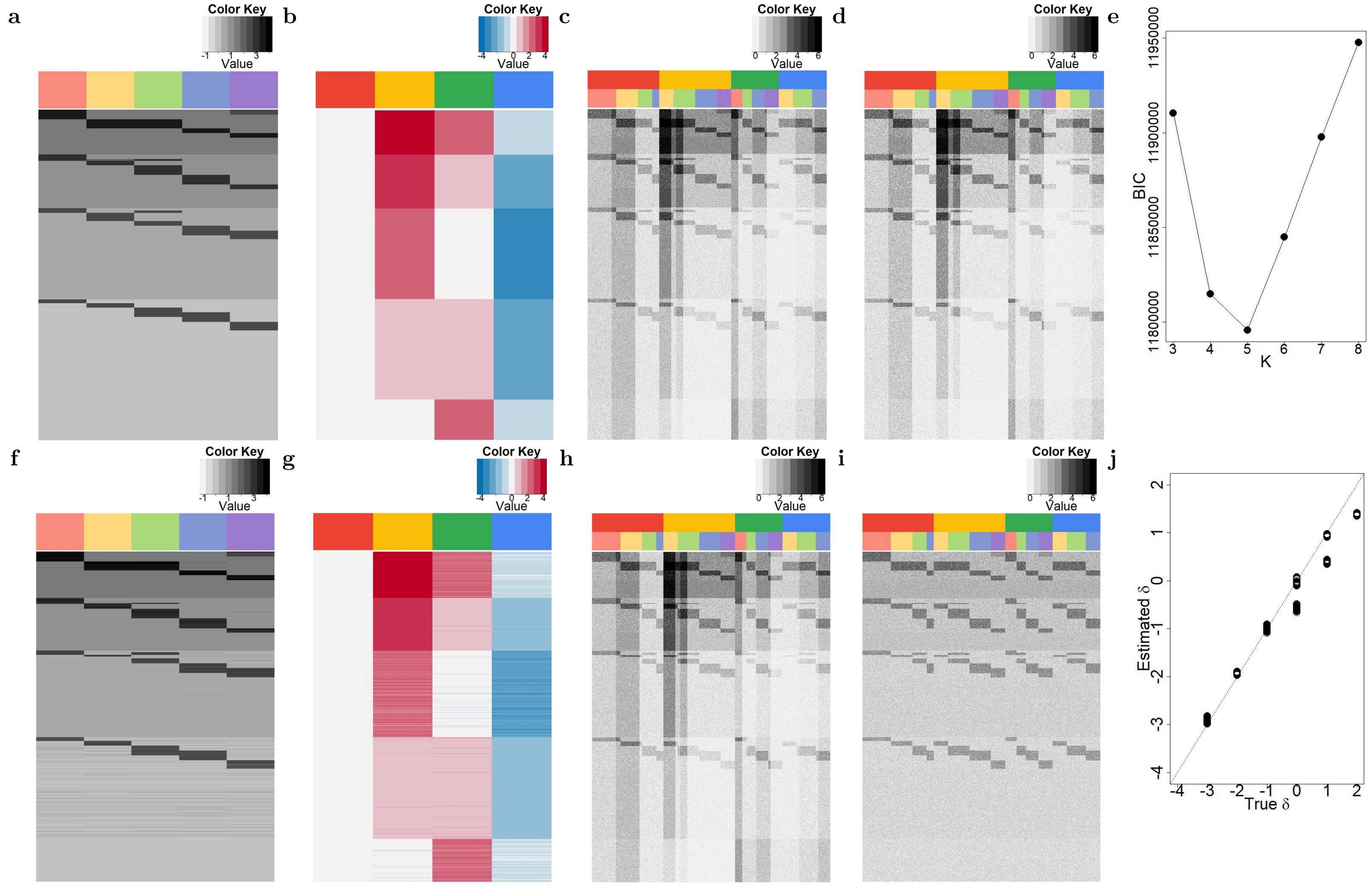

Supplementary Figure 7: Patterns of the simulation dataset under which gene filtering is conducted before applying BUSseq. **(a)** True log-scale mean expression level for each cell type $\boldsymbol{\alpha} + \boldsymbol{\beta}$. Each row represents a gene, and each column corresponds to a cell type. **(b)** True batch effects. Each row represents a gene, and each column corresponds to a batch. **(c)** True underlying expression levels $\boldsymbol{X}$. Each row represents a gene, and each column corresponds to a cell. The upper colored bar indicates the batches, and the lower colored bar represents the cell types. **(d)** The simulated observed data $\boldsymbol{Y}$. **(e)** The BIC plot under different numbers of cell types. **(f)** The estimated log-scale mean expression level for each cell type $\widehat{\boldsymbol{\alpha}} + \widehat{\boldsymbol{\beta}}$. **(g)** Estimated batch effects. **(h)** Imputed expression levels $\widehat{\boldsymbol{X}}$. **(k)** Corrected count data $\widetilde{\boldsymbol{X}}$ grouped by batch. **(l)** Scatter plot of the estimated versus true cell-specific size factors. BUSseq correctly recovers the parameters when BUSseq is applied after conducting gene filtering.

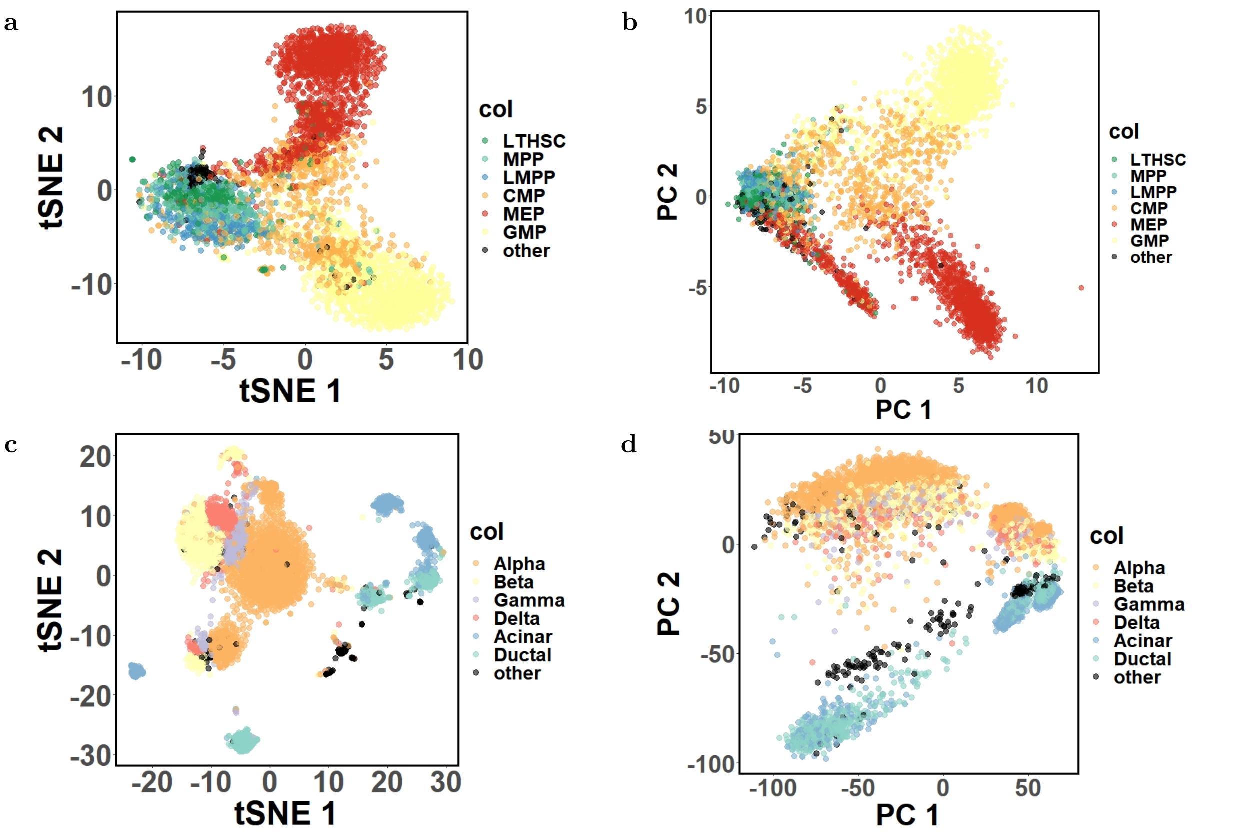

Supplementary Figure 8: t-SNE and PCA plots of the count data after normalization but without batch-effects-correction colored by FACS labels for **(a,b)** the hematopoietic study and **(c,d)** the pancreas study.

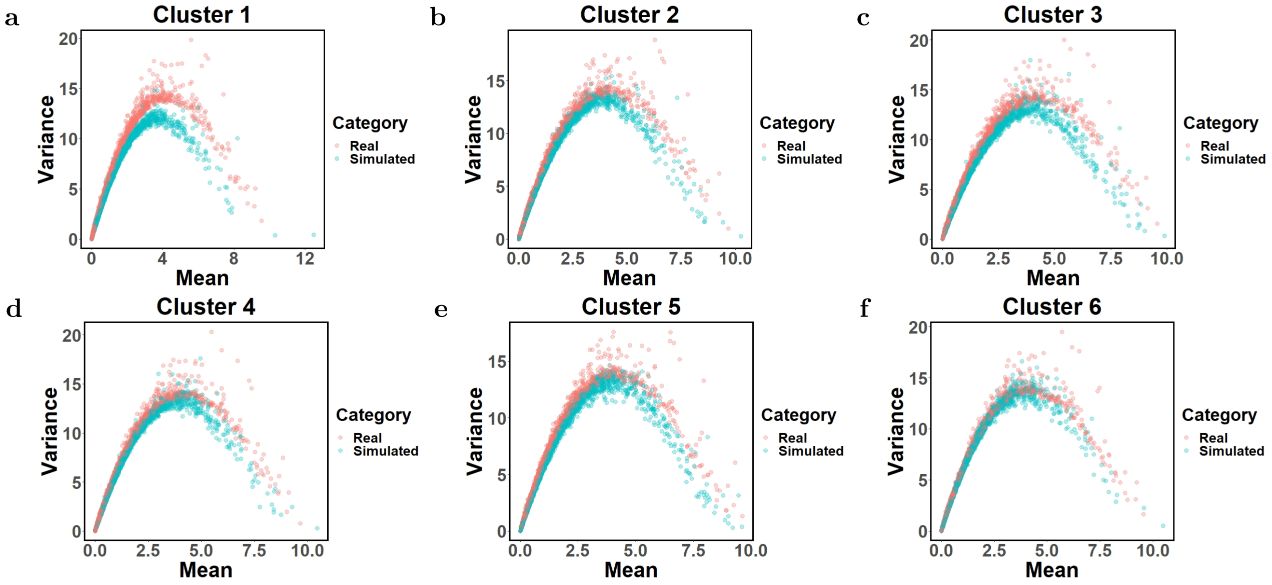

Supplementary Figure 9: Scatter plot of the variance versus the mean of each gene within cluster 1-6 **(a-f)**, respectively, for the hematopoietic study. Red points are the observed values from real data; blue points correspond to the values of the data simulated according to the estimates of BUSseq for the real data.

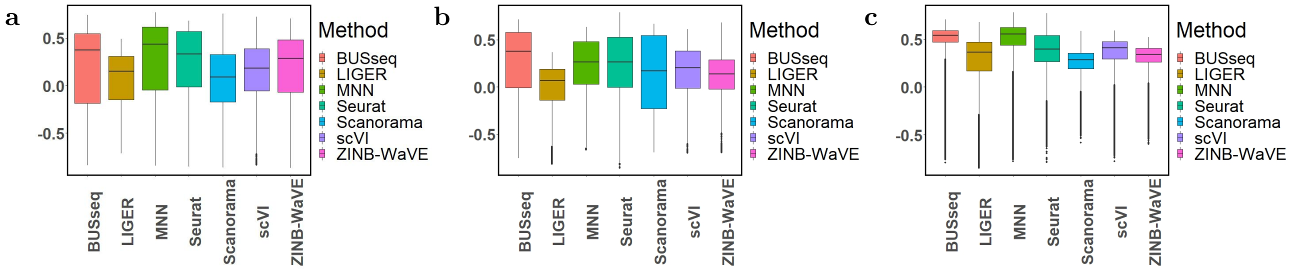

Supplementary Figure 10: Silhouette coefficients of all cells in **(a)** the hematopoietics study based the two-dimensional t-SNE coordinates of the corrected data, **(b)** the hematopoietics study based on the first 10 PCs of the corrected data and **(c)** pancreas study based on the first 10 PCs of the corrected data by each method.

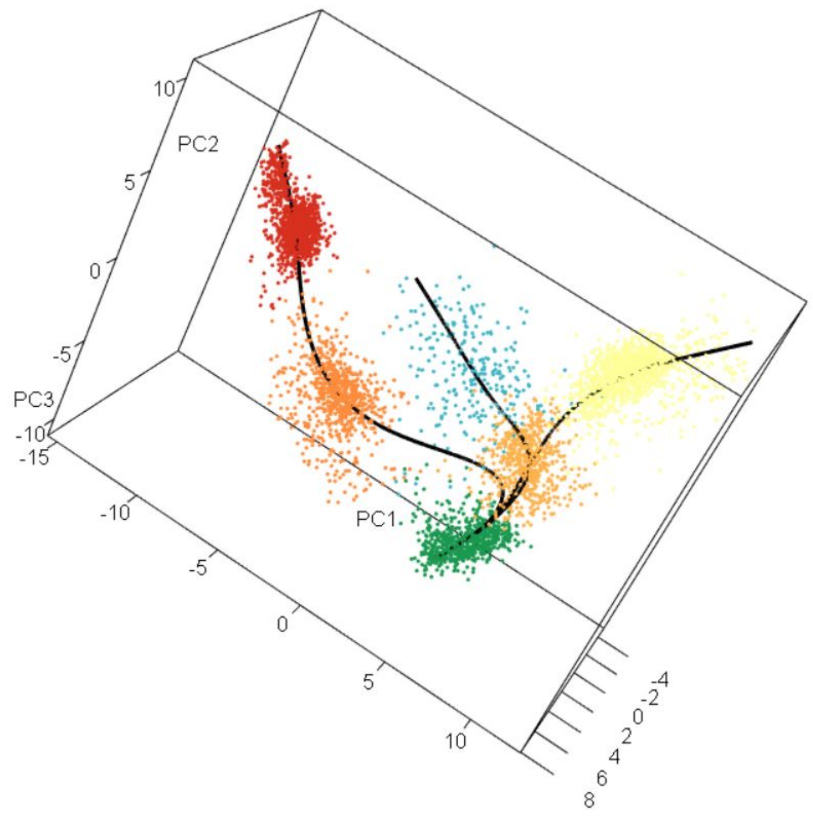

Supplementary Figure 11: The slingshot of hematopoietic studies. Each node represents a cell colored by its estimated cell type label by BUSseq, and the 3-dimensional coordinates of cells correspond to the first three principal components of the corrected count data by BUSseq. The differentiation trajectory from LTHSC or MPP (green cluster in the figure) to MEP (red cluster), GMP (yellow cluster) and LMPP (cyan cluster) can be found.

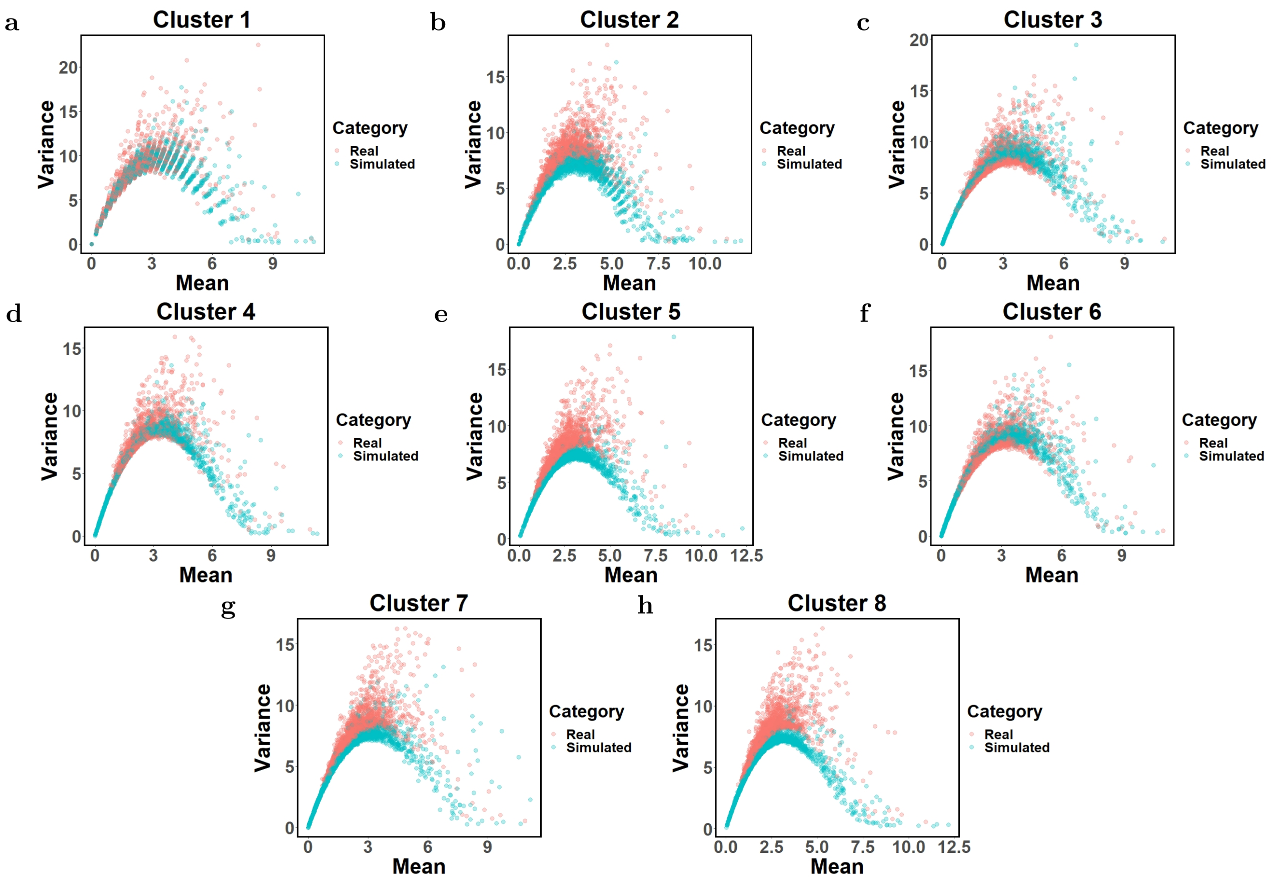

Supplementary Figure 12: Scatter plot of the variance versus the mean of each gene within cluster 1-8 **(a-h)**, respectively, for the pancreas study. Red points are the observed values from real data; blue points correspond to the values of the data simulated according to the estimates of BUSseq for the real data.

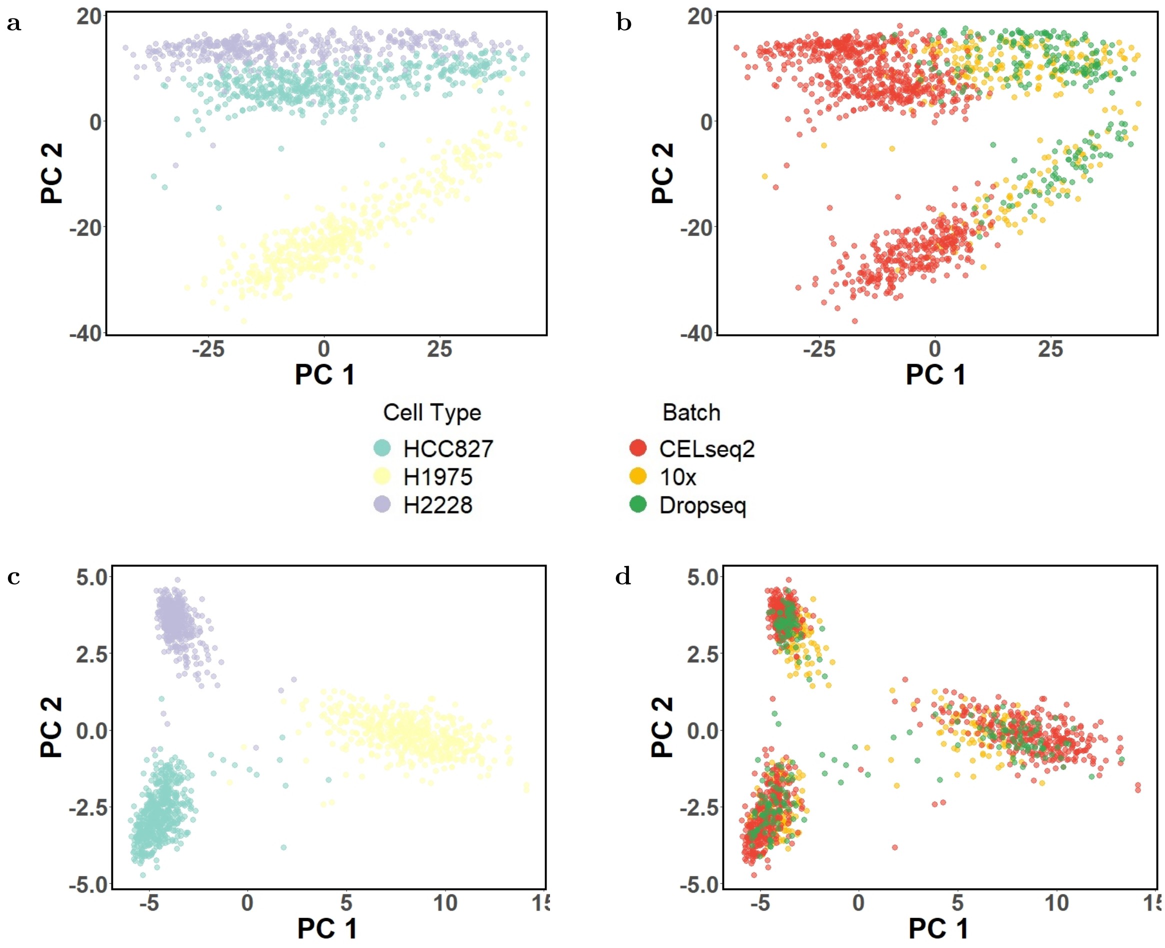

Supplementary Figure 13: PCA plots of the raw count data of three cell lines (H1975, H2228 and HCC827) generated by three protocols (CELseq2, 10x Chromium, and Dropseq) colored **(a)** by cell line and **(b)** by protocol. After correction, PCA plots of the corrected count data by BUSseq colored **(c)** by cell line and **(d)** by protocol.

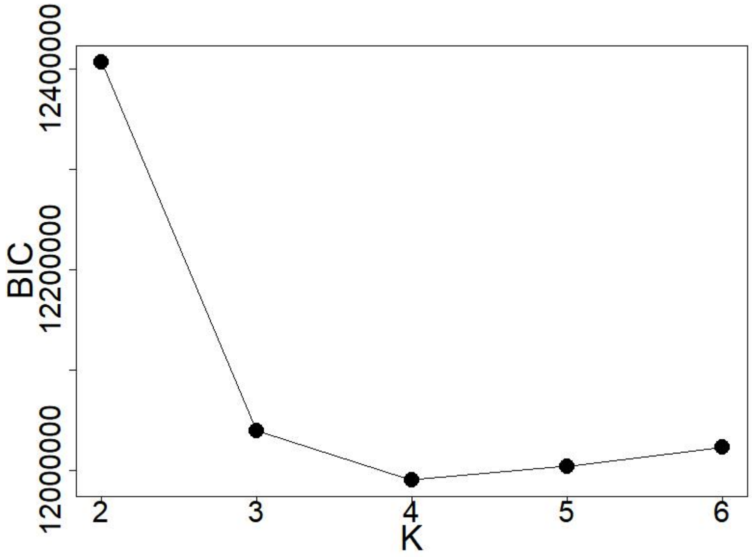

Supplementary Figure 14: BIC curve of the LUAD study.

a

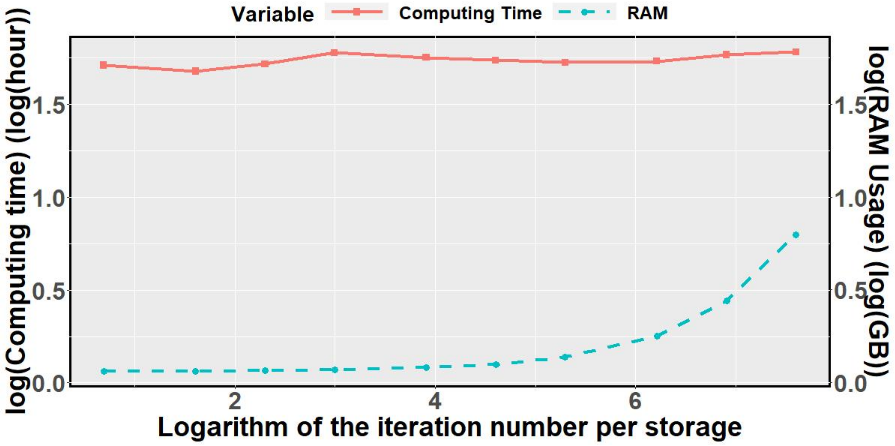

Supplementary Figure 15: Running time and RAM usage for the CPU parallel version of BUSseq on 8 cores of Dual Intel Xeon E5-2650 v2 2.60GHz processors, when n_s varies from 2 to 2,000 with $N = 1,000$ and $G = 3,000$ fixed. This figure is plotted in log-scale.

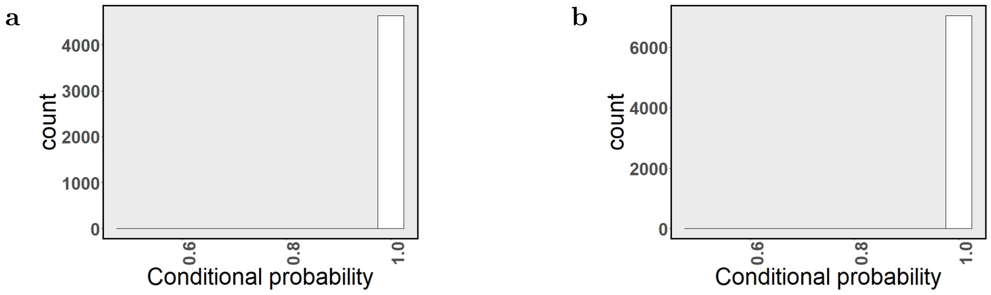

Supplementary Figure 16: Label switching is often observed for low dimensional mixture models, but fortunately, we find there is no label-switching in our inference, according to the histogram of the highest conditional probabilities $\max_k Pr(W_{bi} = k|\widehat{\boldsymbol{X}}, \widehat{\boldsymbol{\Theta}})$ for all cells in **(a)** the hematopoietic study and **(b)** the pancreas study.

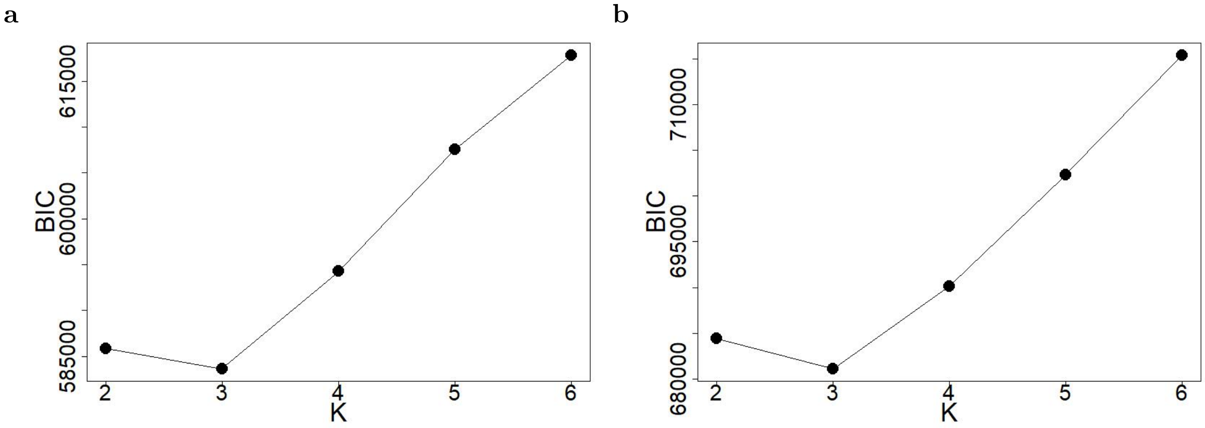

Supplementary Figure 17: BIC curves of **(a)** the three pure cell line mixtures ((9,0,0),(0,9,0) and (0,0,9)) and **(b)** the five cell line mixtures ((9,0,0),(0,9,0), (0,0,9), (4,0,5) and (5,0,4)).

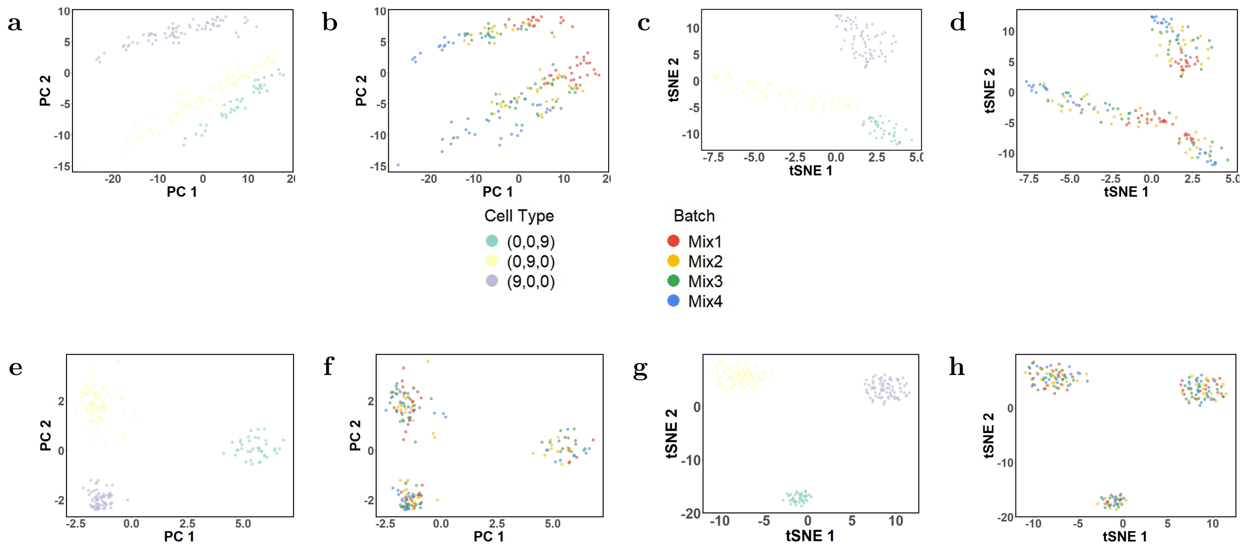

Supplementary Figure 18: PCA and t-SNE plots of three pure cell lines ((9,0,0),(0,9,0) and (0,0,9)) **(a-d)** before and **(e-h)** after BUSseq correction. Each node represents a cell, which is colored by cell line mixture (by batch) in the first (second) column. The first row **(a-b)** denotes the PCA plot of raw count data, while the second row **(c-d)** denotes the t-SNE plot of raw count data. The third row **(e-f)** denotes the PCA plot of the corrected count data by BUSseq, while the forth row **(g-h)** denotes the t-SNE plot of the corrected count data by BUSseq.

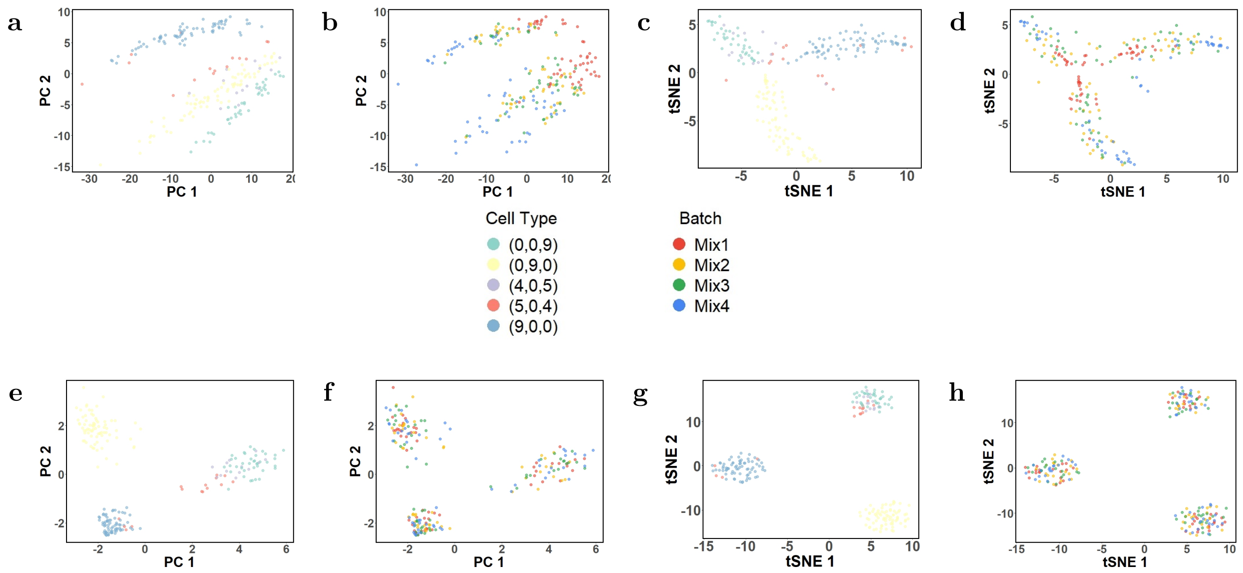

Supplementary Figure 19: PCA and t-SNE plots of five cell line mixtures ((9,0,0),(0,9,0), (0,0,9), (4,0,5) and (5,0,4)) **(a-d)** before and **(e-h)** after BUSseq correction. Each node represents a cell, which is colored by cell line mixture (by batch) in the first (second) column. The first row **(a-b)** denotes the PCA plot of raw count data, while the second row **(c-d)** denotes the t-SNE plot of raw count data. The third row **(e-f)** denotes the PCA plot of the corrected count data by BUSseq, while the forth row **(g-h)** denotes the t-SNE plot of the corrected count data by BUSseq.

Supplementary Tables

The number of cells	Batch 1	Batch 2	Batch 3	Batch 4	Total
Cell Type 1	120	0	48	0	168
Cell Type 2	90	60	0	60	210
Cell Type 3	60	90	40	80	270
Cell Type 4	30	90	52	60	232
Cell Type 5	0	60	60	0	120
Total	300	300	200	200	1000

Supplementary Table 1: Distribution of cells among the four batches of the simulation study in the main text.

Count Data	True	Observed	BUSseq	SAVER	DrImpute	scImpute
d	0	169,525.4	4,115.656	68,894.57	44,003.8	37,254.39
Zero rates	27.92%	44.13%	27.58%	38.12%	22.27%	24.39%

Supplementary Table 2: Comparison of Euclidean distance d and zero rates in the observed count data $\boldsymbol{Y}$ (Observed), the underlying count data $\boldsymbol{X}$ (True), the imputed data by BUSseq, the imputed data by SAVER, the imputed data by DrImpute and the imputed data by scImpute for the first batch of the simulation dataset.

Parameter	Definition	Prior distribution	Hyper-parameters
$\boldsymbol{\pi}_b$	The cell type proportions in batch b	$\boldsymbol{\pi}_b \sim \text{Dir}(\xi, \cdots, \xi)$	$\xi = 2$
γ_{b0}	The odds ratio for the dropout events in the batch b	$\gamma_{b0} \sim \text{N}(0, \sigma^2_{\gamma 0})$	$\sigma^2_{\gamma 0} = 3$
γ_{b1}	The slope of the logistic regression for the dropout events in the batch b	$-\gamma_{b1} \sim \text{Gamma}(a_\gamma, b_\gamma)$	$a_\gamma = 0.001$ and $b_\gamma = 0.01$
α_g	The log-scale baseline expression level of gene g	$\alpha_g \sim \text{N}(m^a_g, \sigma^2_a)$	m^a_g is the empirical estimate of α_g and $\sigma^2_a = 5$
β_{gk}	The cell type effects of gene g in the cell type k	$\beta_{gk} \mid L_{gk} \sim \text{N}(0, \tau^2_{\beta, L_{gk}})$	$\tau^2_{\beta,1} = 50$
p	The proportion of appearing differentially expressed genes	$p \sim \text{Beta}(a_p, b_p)$	$a_p = 1$ and $b_p = 3$
$\tau^2_{\beta,0}$	The variance of the slab prior for the cell type effects	$\tau^2_{\beta,0} \sim \text{Inv-Gamma}(a_\tau, b_\tau)$	$a_\tau = 2$ and $b_\tau = 0.01$
ν_{bg}	The location batch effects of gene g in the batch b	$\nu_{bg} \sim \text{N}(m^c_{bg}, \sigma^2_c)$	m^c_{bg} is the empirical estimate of ν_{bg} and $\sigma^2_c = 5$
δ_{bi}	The size factor of the cell i in the batch b	$\delta_{bi} \sim \text{N}(m^d_{bi}, \sigma^2_d)$	m^d_{bi} is the empirical estimate of δ_{bi} and $\sigma^2_d = 5$
ϕ_{bg}	The overdispersion parameter of gene g in the batch b	$\phi_{bg} \sim \text{Gamma}(a_\phi, b_\phi)$	$a_\phi = 1$ and $b_\phi = 0.1$

Supplementary Table 3: The prior distribution for all parameters.

$\boldsymbol{\pi}_b \sim \text{Dir}(\xi, \xi, \cdots, \xi)$						$p \sim \text{Beta}(a_p, b_p)$					
ξ	0.1	0.5	2	5	10	(a_p, b_p)	(0.1,0.3)	(10,30)	(1,3)	(3,1)	(30, 10)
ARI	1.000	1.000	1.000	1.000	1.000	ARI	1.000	1.000	1.000	1.000	1.000
$\gamma_{b0} \sim \text{N}(0, \sigma^2_{\gamma 0})$						$\tau^2_{\beta,0} \sim \text{Inv-Gamma}(a_\tau, b_\tau)$					
$\sigma^2_{\gamma 0}$	0.3	1.5	3	6	30	ξ	(3,0.02)	(5,0.04)	(2,0.01)	(2,0.1)	(2, 1)
ARI	1.000	1.000	1.000	1.000	1.000	ARI	1.000	1.000	1.000	1.000	1.000
$-\gamma_{b1} \sim \text{Gamma}(a_\gamma, b_\gamma)$						$\nu_{bg} \sim \text{N}(m^c_{bg}, \sigma^2_c)$					
(a_γ, b_γ)	(0.1,0.3)	(1,10)	(0.001,0.01)	(0.01,0.001)	(1,0.1)	σ^2_c	1	2	5	10	100
ARI	1.000	1.000	1.000	1.000	1.000	ARI	0.997	1.000	1.000	1.000	1.000
$\alpha_g \sim \text{N}(m^a_g, \sigma^2_a)$						$\delta_{bi} \sim \text{N}(m^d_{bi}, \sigma^2_d)$					
σ^2_a	1	2	5	10	100	σ^2_d	1	2	5	10	100
ARI	1.000	1.000	1.000	1.000	1.000	ARI	1.000	1.000	1.000	1.000	1.000
$\beta_{gk} \mid L_{gk} \sim \text{N}(0, \tau^2_{\beta, L_{gk}})$						$\phi_{bg} \sim \text{Gamma}(a_\phi, b_\phi)$					
$\tau^2_{\beta,1}$	5	20	50	100	150	(a_ϕ, b_ϕ)	(10,1)	(0.1,0.01)	(1,0.1)	(5,0.1)	(0.1,1)
ARI	1.000	1.000	1.000	1.000	1.000	ARI	1.000	1.000	1.000	1.000	1.000

Supplementary Table 4: Setting of hyperparameter values and their corresponding ARIs as inferred by BUSseq. The third value for each hyperparameter is the setting we use in our inference for simulation and two real datasets.

Method	Output	Clustering	ARI by their own strategies		ARI by k-means clustering	
			Hematopoietic	Pancreas	Hematopoietic	Pancreas
BUSseq	The logarithm of the corrected count data of the identified intrinsic genes	The cluster indicators of mixture model	0.5822	0.6080	0.4757	0.8797
LIGER	The cell-specific factor loadings	SFN graph + Louvain community detection	0.3066	0.5421	0.3188	0.8331
MNN	The logarithm of the corrected count data	SNN graph + Walktrap algorithm	0.5754	0.2793	0.4522	0.4220
Scanorama	The batch-corrected data	k-means	0.5184	0.5272	0.5184	0.5272
scVI	The low-dimensional embedding of cells	k-means	0.1969	0.2819	0.4444	0.5202
Seurat	The first 30 principal components of the corrected data	SNN graph + modularity optimization	0.2663	0.2868	0.3413	0.1828
ZINB-WaVE	The low-dimensional embedding of cells	SNN graph + modularity optimization	0.3484	0.3804	0.4892	0.6404

Supplementary Table 5: Outputs and original clustering strategies of all of the benchmarked methods. We list the ARIs of each method with reference to its original clustering method and the unified k-means clustering, with the number of clusters set to 7 as provided by the FACS labels, respectively.

Ranking	Pathway	p values	Category
1	Hematopoietic cell lineage	1.73×10^{-14}	
2	Cytokine-cytokine receptor interaction	1.84×10^{-12}	Cell growth and differentiation
3	Cell adhesion molecules (CAMs)	3.29×10^{-9}	Immune system
4	Leukocyte transendothelial migration	1.54×10^{-6}	Immune system
5	Primary immunodeficiency	6.75×10^{-6}	Immune system
6	Rap1 signaling pathway	3.44×10^{-5}	Cell growth and differentiation
7	Transcriptional misregulation in cancer	4.23×10^{-5}	
8	Rheumatoid arthritis	6.59×10^{-5}	
9	Pathways in cancer	1.15×10^{-4}	
10	Tuberculosis	1.40×10^{-4}	
11	Malaria	3.02×10^{-4}	
12	Toll-like receptor signaling pathway	3.60×10^{-4}	
13	Staphylococcus aureus infection	4.53×10^{-4}	
14	PI3K-Akt signaling pathway	5.53×10^{-4}	Cell growth and differentiation
15	Osteoclast differentiation	9.74×10^{-4}	Cell growth and differentiation
16	T cell receptor signaling pathway	9.96×10^{-4}	Immune system
17	Intestinal immune network for IgA production	1.43×10^{-3}	Immune system
18	Leishmaniasis	1.44×10^{-3}	Immune system
19	Platelet activation	1.62×10^{-3}	
20	NF-kappa B signaling pathway	1.65×10^{-3}	Immune system
21	Asthma	2.13×10^{-3}	
22	Jak-STAT signaling pathway	2.61×10^{-3}	Cell growth and differentiation
23	B cell receptor signaling pathway	3.34×10^{-3}	Immune system
24	ECM-receptor interaction	3.99×10^{-3}	Cell growth and differentiation
25	Neuroactive ligand-receptor interaction	4.05×10^{-3}	
26	Ras signaling pathway	4.93×10^{-3}	Cell growth and differentiation
27	Pertussis	5.48×10^{-3}	
28	Inflammatory bowel disease (IBD)	6.51×10^{-3}	Immune system
29	Thyroid hormone signaling pathway	9.06×10^{-3}	
30	Phagosome	9.51×10^{-3}	Immune system
31	Mineral absorption	1.09×10^{-2}	
32	Amoebiasis	1.17×10^{-2}	
33	Focal adhesion	1.43×10^{-2}	Cell growth and differentiation
34	Glycosphingolipid biosynthesis - lacto and neolacto series	1.49×10^{-2}	
35	p53 signaling pathway	1.67×10^{-2}	
36	Calcium signaling pathway	1.70×10^{-2}	
37	Fc epsilon RI signaling pathway	1.86×10^{-2}	
38	Natural killer cell mediated cytotoxicity	1.98×10^{-2}	Immune system
39	Proteoglycans in cancer	2.02×10^{-2}	
40	Chemokine signaling pathway	2.40×10^{-2}	Immune system
41	Gastric acid secretion	2.74×10^{-2}	
42	ABC transporters	2.82×10^{-2}	
43	HIF-1 signaling pathway	3.25×10^{-2}	
44	Chagas disease (American trypanosomiasis)	3.50×10^{-2}	
45	Retrograde endocannabinoid signaling	3.50×10^{-2}	
46	NOD-like receptor signaling pathway	3.63×10^{-2}	Immune system
47	Aldosterone-regulated sodium reabsorption	3.77×10^{-2}	
48	Sphingolipid signaling pathway	3.87×10^{-2}	
49	Progesterone-mediated oocyte maturation	4.41×10^{-2}	
50	MAPK signaling pathway	4.58×10^{-2}	Cell growth and differentiation
51	Carbohydrate digestion and absorption	4.76×10^{-2}	

Supplementary Table 6: 51 KEGG pathways (p-value < 0.05 [1]) significantly enriched among the intrinsic genes identified by BUSseq from the hematopoietic data.

Ranking	Pathway	p values	
1	Maturity onset diabetes of the young	9.09×10^{-9}	Diabetes
2	Pancreatic secretion	6.42×10^{-7}	Protein Secretion
3	Insulin secretion	1.58×10^{-6}	Protein Secretion
4	Protein digestion and absorption	1.89×10^{-3}	Metabolism
5	ECM-receptor interaction	7.08×10^{-3}	
6	Type II diabetes mellitus	7.63×10^{-3}	Diabetes
7	Morphine addiction	8.99×10^{-3}	
8	Proteoglycans in cancer	1.44×10^{-2}	
9	Dopaminergic synapse	1.76×10^{-2}	
10	GABAergic synapse	2.24×10^{-2}	
11	Type I diabetes mellitus	2.26×10^{-2}	Diabetes
12	Tight junction	2.48×10^{-2}	
13	Drug metabolism - cytochrome P450	3.08×10^{-2}	Metabolism
14	Focal adhesion	4.07×10^{-2}	

Supplementary Table 7: 14 KEGG pathways (p-value< 0.05 [1]) significantly enriched among the intrinsic genes identified by BUSseq from the pancreatic data.

	HCC827	H1975	H2228
1	1	317	2
2	1	191	0
3	424	0	0
4	0	3	462

Supplementary Table 8: Contingency table between the estimated cell types $\hat{w}_{bi} \in \{1, 2, 3, 4\}$ and the known cell-line labels.

	F1 score	Accuracy	ARI
BUSseq	0.9971	0.9979	0.9933
CellAssign	0.9892	0.9907	0.9721

Supplementary Table 9: Comparison of F1 score, Accuracy and ARI between BUSseq and CellAssign in the LUAD dataset.

Study	Simulation	Mouse Hematpoietic	Human Pancreas
Number of cell N	1,000	4,649	7,095
Number of highly variable genes G	3,000	3,470	2,480
Number of batches B	4	2	4
Number of cell types K	5	6	8
Total number of iterations in MCMC	4,000	8,000	8,000
Number of burn-in iterations	2,000	4,000	4,000
CPU Runtime (hours)	1.00	8.00	12.09
GPU Runtime (hours)	0.35	1.15	1.50

Supplementary Table 10: Summary and runtime of BUSseq in the simulation and case studies. The CPU runtime records the time consumption of running BUSseq on eight Intel Xeon Gold 6128 CPU cores in parallel, whereas the GPU runtime records the time consumption of running BUSseq with a single core of an Intel Xeon Gold 6132 CPU of 512GM RAM and one NVIDIA Tesla P100 GPU of 16GM RAM.

$G = 3000$ and $n_s = 1000$					
N	1000	2000	4000	10000	20000
Running time (hour)	1.768	3.263	6.343	16.426	29.702
RAM peak usage (GB)	0.442	0.509	0.643	1.046	1.726
$N = 1000$ and $n_s = 1000$					
G	1500	3000	4500	6000	9000
Running time (hour)	0.868	1.768	2.416	3.411	5.123
RAM peak usage (GB)	0.229	0.442	0.661	0.867	1.291
$N = 1000$ and $G = 3000$					
n_s	100	200	500	1000	2000
Running time (hour)	1.738	1.726	1.733	1.768	1.783
RAM peak usage (GB)	0.100	0.138	0.252	0.442	0.799
n_s	2	5	10	20	50
Running time (hour)	1.711	1.679	1.718	1.778	1.752
RAM peak usage (GB)	0.062	0.062	0.065	0.069	0.081

Supplementary Table 11: Trend of running time and RAM usage for the CPU code with respect to the number of cells, the number of genes and the number of iterations per storage, respectively. The CPU code are run on 8 cores of Dual Intel Xeon E5-2650 v2 2.60GHz processors.

Study	m	Total number of iterations	Number of Burn-ins	$\{\text{EPSR}(\theta_{gk})\} < 1.3$	$\{\text{EPSR}(\nu_{bg})\} < 1.3$	$\{\text{EPSR}(\phi_{gk})\} < 1.3$
Simulation	4	4,000	2,000	98.29%	98.19%	99.09%
Hematopoietic	4	8,000	4,000	89.09%	88.73%	97.94%
Pancreas	4	8,000	4,000	84.08%	86.03%	97.96%

Supplementary Table 12: EPSR factors of $\{\text{EPSR}(\theta_{gk})\}$, $\{\text{EPSR}(\nu_{bg})\}$ and $\{\text{EPSR}(\phi_{bg})\}$ in the simulation study and the two real applications. The last three columns show the proportion of the corresponding EPSR factors less than 1.3

Study	Parameter	Min	First quartile	Median	Mean	Third quartile	Max
Simulation	$\boldsymbol{\alpha}$	0.196	0.267	0.378	0.352	0.415	0.526
	$\boldsymbol{\beta}$	0.209	0.258	0.274	0.290	0.286	0.850
	$\boldsymbol{\nu}$	0.293	0.486	0.625	0.596	0.702	0.850
	$\boldsymbol{\delta}$	0.116	0.172	0.198	0.208	0.233	0.338
	$\boldsymbol{\phi}$	0.132	0.352	0.499	0.529	0.740	0.873
	$\boldsymbol{\gamma}$	0.010	0.012	0.025	0.026	0.038	0.044
Hematopoietic	$\boldsymbol{\alpha}$	0.073	0.356	0.471	0.488	0.619	0.951
	$\boldsymbol{\beta}$	0.122	0.622	0.723	0.705	0.816	0.991
	$\boldsymbol{\nu}$	0.122	0.432	0.525	0.541	0.646	0.961
	$\boldsymbol{\delta}$	0.234	0.367	0.379	0.422	0.462	0.722
	$\boldsymbol{\phi}$	0.002	0.011	0.014	0.016	0.019	0.097
	$\boldsymbol{\gamma}$	0.001	0.012	0.049	0.217	0.254	0.771
Pancreas	$\boldsymbol{\alpha}$	0.058	0.177	0.206	0.215	0.246	0.433
	$\boldsymbol{\beta}$	0.116	0.408	0.476	0.490	0.565	0.951
	$\boldsymbol{\nu}$	0.088	0.279	0.366	0.415	0.553	0.798
	$\boldsymbol{\delta}$	0.135	0.283	0.304	0.340	0.441	0.515
	$\boldsymbol{\phi}$	0.006	0.015	0.032	0.152	0.173	0.857
	$\boldsymbol{\gamma}$	0.004	0.014	0.020	0.038	0.027	0.179

Supplementary Table 13: Acceptance rates of all BUSseq parameters updated by Metropolis steps in the simulation study and two real applications.

Parameter	γ_{10}	γ_{11}	γ_{20}	γ_{21}	γ_{30}	γ_{31}	γ_{40}	γ_{41}
True values	-0.500	-0.200	-0.500	-0.200	-0.500	-0.200	-0.500	-0.200
Posterior mean	-0.475	-0.200	-0.461	-0.204	-0.461	-0.201	-0.443	-0.201

Supplementary Table 14: Comparison between true values and posterior mean for $\boldsymbol{\gamma}$ in the simulation study. $\boldsymbol{\gamma}$ can be precisely estimated by MCMC algorithm, even if the acceptance rate is low.

Cell line mixture	Batch 1	Batch 2	Batch 3	Batch 4
(0,0,9)	9	10	10	10
(0,9,0)	19	19	19	19
(4,0,5)	3	2	4	4
(5,0,4)	4	3	4	4
(9,0,0)	18	18	18	18

Supplementary Table 15: Distribution of each cell line mixture in the four batches.

The number of HCC827	$m = 18$	$m = 10$	$m = 8$	$m = 6$	$m = 5$	$m = 4$	$m = 3$	$m = 2$	$m = 1$
ARI	1.00	1.00	1.00	1.00	1.00	1.00	1.00	1.00	0.785

Supplementary Table 16: ARI between the known cell line types and the estimated cell type indicators by BUSseq under different downsampling levels of the HCC827 cells.

HVG / TEG	Cluster1 (LTHSC & MPP)	Cluster2 (CMP)	Cluster3 (CLP)	Cluster4 (CMP & MEP)	Cluster5 (GMP)	Cluster6 (MEP)
Cluster1 (LTHSC & MPP)	556	80	6	6	1	0
Cluster2 (CMP)	66	171	52	80	60	1
Cluster3 (CLP)	2	0	164	0	0	0
Cluster4 (CMP & MEP)	1	5	5	573	0	79
Cluster5 (GMP)	14	315	24	66	1216	0
Cluster6 (MEP)	0	0	2	19	0	1085

Supplementary Table 17: Contingency table between the cell type labeling learned by BUSseq when filtering HVGs (columns) and filtering genes with high mean expression levels (rows) in the hematopoietic study.

HVG / TEG	Cluster1 (Alpha)	Cluster2 (Alpha)	Cluster3 (Alpha)	Cluster4 (Beta)	Cluster5 (Gamma & Delta)	Cluster6 (Acinar)	Cluster7 (Ductal)	Cluster8 (Other)
Cluster1 (Alpha)	1452	143	7	0	73	0	0	0
Cluster2 (Alpha)	194	678	106	447	139	1	1	0
Cluster3 (Beta)	5	9	59	6	8	7	7	20
Cluster4 (Beta)	2	2	6	785	192	0	0	0
Cluster5 (Gamma & Delta)	22	285	4	14	559	1	0	0
Cluster6 (Acinar)	0	0	3	1	1	625	9	0
Cluster7 (Ductal)	0	0	8	0	0	66	916	6
Cluster8 (Other)	0	0	0	0	0	1	2	223

Supplementary Table 18: Contingency table between the cell type labeling learned by BUSseq when filtering HVGs (columns) and filtering genes with high mean expression levels (rows) in the pancreas study.

Supplementary Notes

Supplementary Note 1: BUSseq model

The hierarchical model of BUSseq can be summarized as:

$$\begin{aligned}
&Pr(W_{bi}=k)=\pi_{bk}, \sum_{k=1}^{K}\pi_{bk}=1;\\
&X_{big}|W_{bi}=k\sim NB(\mu_{big},\phi_{bg}), \quad log(\mu_{big})=\alpha_g+\beta_{gk}+\nu_{bg}+\delta_{bi};\\
&Z_{big}|X_{big}=x_{big}\sim Bernoulli(p_{big}), \quad log(\frac{p_{big}}{1-p_{big}})=\gamma_{b0}+\gamma_{b1}x_{big};\\
&Y_{big}=X_{big}|Z_{big}=0, \quad Y_{big}=0|Z_{big}=1. \qquad (1)
\end{aligned}$$

Collectively, $\boldsymbol{Y}=\{Y_{big}\}_{b=1,\cdots,B;i=1,\cdots,n_b}^{g=1,\cdots,G}$ are the observed data; the underlying expression levels $\boldsymbol{X}=\{X_{big}\}_{b=1,\cdots,B;i=1,\cdots,n_b}^{g=1,\cdots,G}$, the dropout indicators $\boldsymbol{Z}=\{Z_{big}\}_{b=1,\cdots,B;i=1,\cdots,n_b}^{g=1,\cdots,G}$ and the cell type indicators $\boldsymbol{W}=\{W_{bi}\}_{b=1,\cdots,B;i=1,\cdots,n_b}$ are all missing data; the log-scale baseline gene expression levels $\boldsymbol{\alpha}=\{\alpha_g\}_{g=1,\cdots,G}$, the cell type effects $\boldsymbol{\beta}=\{\beta_{gk}\}_{k=2,\cdots,K}^{g=1,\cdots,G}$, the location batch effects $\boldsymbol{\nu}=\{\nu_{bg}\}_{b=2,\cdots,B}^{g=1,\cdots,G}$, the overdispersion parameters $\boldsymbol{\phi}=\{\phi_{bg}\}_{b=1,\cdots,B}^{g=1,\cdots,G}$, the cell-specific size factors $\boldsymbol{\Delta}=\{\delta_{bi}\}_{b=1,\cdots,B}^{i=2,\cdots,n_b}$, the dropout parameters $\boldsymbol{\Gamma}=\{\gamma_{b0},\gamma_{b1}\}^{b=1,\cdots,B}$ and the cell compositions $\boldsymbol{\pi}=\{\pi_{bk}\}_{b=1,\cdots,B}^{k=1,\cdots,K}$ are the parameters. Without loss of generality, for model identifiability, we assume that the first batch is the reference batch measured without batch effects with $\nu_{1g}=0$ for every gene and the first cell type is the baseline cell type with $\beta_{g1}=0$ for every gene. Similarly, we take the cell-specific size factor $\delta_{b1}=0$ for the first cell of each batch. We gather all the parameters as $\boldsymbol{\Theta}=\{\boldsymbol{\alpha},\boldsymbol{\beta},\boldsymbol{\nu},\boldsymbol{\phi},\boldsymbol{\Delta},\boldsymbol{\Gamma},\boldsymbol{\pi}\}$. Let $f_{\text{NB}}(x;\mu,\phi)=C_x^{\phi+x-1}(\frac{\mu}{\mu+\phi})^x(\frac{\phi}{\mu+\phi})^\phi$ denote the probability mass function (PMF) of the negative binomial distribution $NB(\mu,\phi)$, where C_k^n is the binomial coefficient, then the complete data likelihood function equals to:

$$\begin{aligned}
L_c(\boldsymbol{\Theta}|\boldsymbol{y},\boldsymbol{x},\boldsymbol{z},\boldsymbol{w})=\prod_{b=1}^{B}\prod_{i=1}^{n_b}\prod_{k=1}^{K}\{\pi_{bk}\prod_{g=1}^{G}[I(y_{big}=x_{big}(1-z_{big}))\frac{exp[(\gamma_{b0}+\gamma_{b1}x_{big})z_{big}]}{1+exp(\gamma_{b0}+\gamma_{b1}x_{big})}\\
\cdot f_{\text{NB}}(x_{big};exp(\alpha_g+\beta_{gk}+\nu_{bg}+\delta_{bi}),\phi_{bg})]\}^{I(w_{bi}=k)}. \qquad (2)
\end{aligned}$$

Besides, the observed data likelihood function becomes

$$L_o(\boldsymbol{\Theta}|\boldsymbol{y})=\prod_{b=1}^{B}\prod_{i=1}^{n_b}[\sum_{k=1}^{K}\pi_{bk}\prod_{g=1}^{G}Pr(Y_{big}=y_{big}|\boldsymbol{\Theta})], \qquad (3)$$

where

$$Pr(Y_{big} = y_{big}|\mathbf{\Theta}) = \begin{cases} \sum_{x=1}^{\infty} \frac{exp(\gamma_{b0}+\gamma_{b1}x)}{1+exp(\gamma_{b0}+\gamma_{b1}x)} f_{\mathrm{NB}}(x; exp(\alpha_g + \beta_{gk} + \nu_{bg} + \delta_{bi}), \phi_{bg}) & \\ +f_{\mathrm{NB}}(0; exp(\alpha_g + \beta_{gk} + \nu_{bg} + \delta_{bi}), \phi_{bg}) & y_{big} = 0, \\ \frac{1}{1+exp(\gamma_{b0}+\gamma_{b1}y_{big})} f_{\mathrm{NB}}(y_{big}; exp(\alpha_g + \beta_{gk} + \nu_{bg} + \delta_{bi}), \phi_{bg}) & y_{big} > 0. \end{cases}$$

Supplementary Note 2: Markov chain Monte Carlo (MCMC) algorithm

We develop an MCMC algorithm under a Bayesian framework (Supplementary Information). First, we assign independent priors to all of the parameters with hyper-parameters specified as shown in **Supplementary Tab. 3**.

Here, we detail out how we obtain the empirical estimates for m_g^as, m_{bg}^cs and m_{bi}^ds. We first give a crude estimate of the cell size factor δ_{bi} by taking the logarithm of the ratio of the total read counts of cell i over the total read counts of the first cell in batch b:

$$m_{bi}^d = log(\frac{\sum_{g=1}^G y_{big}}{\sum_{g=1}^G y_{b1g}}). \tag{4}$$

Then, we randomly assign labels $w_{bi}^{(0)}$ to all cells. Because we assume $\beta_{g1} = 0$ and $\nu_{1g} = 0$, the cells in the first batch and the first cell type have the mean expression level $exp(\alpha_g + \delta_{1i})$. Thus, we estimate the log-scale baseline expression levels α_g as following:

$$m_g^a = \frac{\sum_{i=1}^{n_1} I(w_{1i}^{(0)} = 1) log(1 + \frac{Y_{1ig}}{exp(m_{1i}^d)})}{\sum_{i=1}^{n_1} I(w_{1i}^{(0)} = 1) + 1}, \tag{5}$$

where we add one to the denominator to ensure that the denominator is positive. Furthermore, we compare the expression levels of the cells assigned to the first cell type across different batches to estimate the batch effects.

$$m_{bg}^c = \frac{\sum_{i=1}^{n_b} I(w_{bi}^{(0)} = 1) log(1 + \frac{Y_{big}}{exp(m_{bi}^d)})}{\sum_{i=1}^{n_b} I(w_{bi}^{(0)} = 1) + 1} - \frac{\sum_{i=1}^{n_1} I(w_{1i}^{(0)} = 1) log(1 + \frac{Y_{1ig}}{exp(m_{1i}^d)})}{\sum_{i=1}^{n_1} I(w_{1i}^{(0)} = 1) + 1}. \tag{6}$$

We then draw a series of samples from the posterior distribution. After the burn-in period, we take the mean of the posterior samples to estimate $\boldsymbol{\gamma}_b, \alpha_g, \beta_{gk}, \nu_{bg}, \delta_{bi}$ and ϕ_{bg} and use the mode of posterior samples of W_{bi} to infer the cell type for each cell. The posterior distribution is derived as following. At iteration t:

1. Update $z_{big}^{[t]}$ and $x_{big}^{[t]}$ sequentially for (b, i, g), if $y_{big} = 0$:

$$z_{big}^{[t]} \begin{cases} = 1 & , \text{ if } x_{big}^{[t-1]} > 0; \\ \sim Bernoulli(\frac{exp(\gamma_{b0}^{[t-1]})}{1+exp(\gamma_{b0}^{[t-1]})}) & , \text{ if } x_{big}^{[t-1]} = 0. \end{cases}$$

$$x_{big}^{[t]} \begin{cases} = 0 & , \text{ if } z_{big}^{[t]} = 0; \\ \propto \frac{exp(\gamma_{b0}^{[t-1]}+\gamma_{b1}^{[t-1]}x_{big}^{[t]})}{1+exp(\gamma_{b0}^{[t-1]}+\gamma_{b1}^{[t-1]}x_{big}^{[t]})} \frac{\Gamma(\phi_{bg}^{[t-1]}+x_{big}^{[t]})(\mu_{big}^{[t-1]})^{x_{big}^{[t]}}}{\Gamma(x_{big}^{[t]})(\phi_{bg}^{[t-1]}+\mu_{big}^{[t-1]})^{\phi_{bg}^{[t-1]}+x_{big}^{[t]}}} & , \text{ if } z_{big}^{[t]} = 1. \end{cases}$$

where $\mu_{big}^{[t-1]} = exp(\alpha_g^{[t-1]} + \beta_{gw_{bi}^{[t-1]}}^{[t-1]} + \nu_{bg}^{[t-1]} + \delta_{bi}^{[t-1]})$, and $\Gamma(\cdot)$ represents the Gamma function. When $z_{big}^{[t]} = 1$, we find that

$$\begin{aligned} f(x|\gamma_{b0}^{[t-1]}, \gamma_{b1}^{[t-1]}, \mu_{big}^{[t-1]}, \phi_{bg}^{[t-1]}) &\propto \frac{exp(\gamma_{b0}^{[t-1]} + \gamma_{b1}^{[t-1]}x)}{1 + exp(\gamma_{b0}^{[t-1]} + \gamma_{b1}^{[t-1]}x)} \cdot \frac{\Gamma(\phi_{bg}^{[t-1]} + x)(\mu_{big}^{[t-1]})^x}{\Gamma(x)(\phi_{bg}^{[t-1]} + \mu_{big}^{[t-1]})^{\phi_{bg}^{[t-1]}+x}} \\ &\propto \frac{exp(\gamma_{b0}^{[t-1]} + \gamma_{b1}^{[t-1]}x)}{1 + exp(\gamma_{b0}^{[t-1]} + \gamma_{b1}^{[t-1]}x)} \cdot p_{\text{NB}}(x|\mu_{big}^{[t-1]}, \phi_{bg}^{[t-1]}), \end{aligned}$$

where $p_{\text{NB}}(x|\mu, \phi)$ denotes the PMF of the negative binomial distribution with mean μ and overdispersion ϕ. Therefore, we incorporate a Metropolis-Hasting (MH) step [2]. We sample x_{big}^* from the proposal distribution $NB(\mu_{big}^{[t-1]}, \phi_{bg}^{[t-1]})$ and accept the proposal with probability

$$\begin{aligned} \rho &= \min\{\frac{f(x_{big}^*|\gamma_{b0}^{[t-1]}, \gamma_{b1}^{[t-1]}, \mu_{big}^{[t-1]}, \phi_{bg}^{[t-1]})p_{\text{NB}}(x_{big}^{[t-1]}|\mu_{big}^{[t-1]}, \phi_{bg}^{[t-1]})}{f(x_{big}^{[t-1]}|\gamma_{b0}^{[t-1]}, \gamma_{b1}^{[t-1]}, \mu_{big}^{[t-1]}, \phi_{bg}^{[t-1]})p_{\text{NB}}(x_{big}^*|\mu_{big}^{[t-1]}, \phi_{bg}^{[t-1]})}, 1\} \\ &= \min\{\frac{\frac{exp(\gamma_{b0}^{[t-1]}+\gamma_{b1}^{[t-1]}x_{big}^*)}{1+exp(\gamma_{b0}^{[t-1]}+\gamma_{b1}^{[t-1]}x_{big}^*)}p_{\text{NB}}(x_{big}^*|\mu_{big}^{[t-1]}, \phi_{bg}^{[t-1]})p_{\text{NB}}(x_{big}^{[t-1]}|\mu_{big}^{[t-1]}, \phi_{bg}^{[t-1]})}{\frac{exp(\gamma_{b0}^{[t-1]}+\gamma_{b1}^{[t-1]}x_{big}^{[t-1]})}{1+exp(\gamma_{b0}^{[t-1]}+\gamma_{b1}^{[t-1]}x_{big}^{[t-1]})}p_{\text{NB}}(x_{big}^{[t-1]}|\mu_{big}^{[t-1]}, \phi_{bg}^{[t-1]})p_{\text{NB}}(x_{big}^*|\mu_{big}^{[t-1]}, \phi_{bg}^{[t-1]})}, 1\} \\ &= \min\{\frac{\frac{exp(\gamma_{b0}^{[t-1]}+\gamma_{b1}^{[t-1]}x_{big}^*)}{1+exp(\gamma_{b0}^{[t-1]}+\gamma_{b1}^{[t-1]}x_{big}^*)}}{\frac{exp(\gamma_{b0}^{[t-1]}+\gamma_{b1}^{[t-1]}x_{big}^{[t-1]})}{1+exp(\gamma_{b0}^{[t-1]}+\gamma_{b1}^{[t-1]}x_{big}^{[t-1]})}}, 1\} \\ &= \min\{\frac{1 + exp(-\gamma_{b0}^{[t-1]} - \gamma_{b1}^{[t-1]}x_{big}^{[t-1]})}{1 + exp(-\gamma_{b0}^{[t-1]} - \gamma_{b1}^{[t-1]}x_{big}^*)}, 1\}. \end{aligned}$$

On the other hand, if $y_{big} > 0$, then $z_{big}^{[t]} = 0$ and $x_{big}^{[t]} = y_{big}$.

2. Update $\gamma_{b0}^{[t]}$ and $\gamma_{b1}^{[t]}$ sequentially. Because

$$L(\gamma_b^{[t]}) \propto \prod_{i=1}^{n_b}\prod_{g=1}^{G} \frac{exp[(\gamma_{b1}^{[t]} x_{big}^{[t]} + \gamma_{b0}^{[t]}) z_{big}^{[t]}]}{1 + exp(\gamma_{b1}^{[t]} x_{big}^{[t]} + \gamma_{b0}^{[t]})} \cdot exp(-\frac{(\gamma_{b0}^{[t]})^2}{2\sigma_{\gamma 0}^2}) \cdot (-\gamma_{b1}^{[t]})^{a_\gamma - 1} exp(b_\gamma \gamma_{b1}^{[t]}),$$

we update γ_{b0} by an MH step with the symmetric proposal distribution $g(\gamma_{b0}^*|\gamma_{b0}^{[t-1]}) \sim \mathrm{N}(\gamma_{b0}^{[t-1]}, \sigma_{\mathrm{MH}}^2)$. Consequently, the acceptance rate is

$$\begin{aligned}\rho &= \min\{\frac{L(\gamma_{b0}^*|-)}{L(\gamma_{b0}^{[t-1]}|-)}, 1\} \\ &= \min\{\prod_{i=1}^{n_b}\prod_{g=1}^{G} \frac{exp(\gamma_{b0}^* z_{big}^{[t]})[1 + exp(\gamma_{b1}^{[t-1]} x_{big}^{[t]} + \gamma_{b0}^{[t-1]})]}{exp(\gamma_{b0}^{[t-1]} z_{big}^{[t]})[1 + exp(\gamma_{b1}^{[t-1]} x_{big}^{[t]} + \gamma_{b0}^*)]} \cdot exp(-\frac{(\gamma_{b0}^*)^2 - (\gamma_{b0}^{[t-1]})^2}{2\sigma_{\gamma 0}^2}), 1\}.\end{aligned}$$

To update γ_{b1}, we incorporate an MH step with the proposal distribution $g(-\gamma_{b1}^*|\gamma_{b1}^{[t-1]}) \sim \mathrm{Gamma}(-10\gamma_{b1}^{[t-1]}, 10)$, and the acceptance rate being

$$\begin{aligned}\rho &= \min\{\frac{L(\gamma_{b1}^*|-)}{L(\gamma_{b1}^{[t-1]}|-)}, 1\} \\ &= \min\{\prod_{i=1}^{n_b}\prod_{g=1}^{G} \frac{exp(\gamma_{b1}^* x_{big}^{[t]} z_{big}^{[t]})[1 + exp(\gamma_{b1}^{[t-1]} x_{big}^{[t]} + \gamma_{b0}^{[t]})]}{exp(\gamma_{b1}^{[t-1]} x_{big}^{[t]} z_{big}^{[t]})[1 + exp(\gamma_{b1}^* x_{big}^{[t]} + \gamma_{b0}^{[t]})]} \\ &\cdot \frac{(-\gamma_{b1}^{[t-1]})^{-a_\gamma - 10\gamma_{b1}^*} 10^{-10\gamma_{b1}^*} \Gamma(-10\gamma_{b1}^{[t-1]})}{(-\gamma_{b1}^*)^{-a_\gamma - 10\gamma_{b1}^{[t-1]}} 10^{-10\gamma_{b1}^{[t-1]}} \Gamma(-10\gamma_{b1}^*)} exp[(10 - b_\gamma)(\gamma_{b1}^{[t-1]} - \gamma_{b1}^*)], 1\}.\end{aligned}$$

3. For each gene g, we use an MH step to update α_g. Specifically, we let the proposal distribution be the symmetric $g(\alpha_g^*|\alpha_g^{[t-1]}) \sim \mathrm{N}(\alpha_g^{[t-1]}, \sigma_{\mathrm{MH}}^2)$ and the acceptance rate be:

$$\begin{aligned}\rho &= \min\{\frac{L(\alpha_g^*|-)}{L(\alpha_g^{[t-1]}|-)}, 1\} \\ &= \min\{\prod_{b=1}^{B}\prod_{i=1}^{n_b} exp((\alpha_g^* - \alpha_g^{[t-1]}) x_{big}^{[t]}) (\frac{\phi_{bg}^{[t-1]} + exp(\alpha_g^{[t-1]} + \beta_{gw_{bi}^{[t-1]}}^{[t-1]} + \nu_{bg}^{[t-1]} + \delta_{bi}^{[t-1]})}{\phi_{bg}^{[t-1]} + exp(\alpha_g^* + \beta_{gw_{bi}^{[t-1]}}^{[t-1]} + \nu_{bg}^{[t-1]} + \delta_{bi}^{[t-1]})})^{\phi_{bg}^{[t-1]} + x_{big}^{[t]}} \\ &\cdot exp(-\frac{(\alpha_g^* - m_g^a)^2 - (\alpha_g^{[t-1]} - m_g^a)^2}{2\sigma_a^2}), 1\}.\end{aligned}$$

4. For each gene g and for $2 \le k \le K$, we sample the indicator $L_{gk}^{[t]}$ from:

$$L_{gk}^{[t]} \sim \text{Bernoulli}(\frac{p^{[t-1]}\text{N}(\beta_{gk}^{[t-1]}; 0, (\tau_{\beta 1}^{[t-1]})^2)}{p^{[t-1]}\text{N}(\beta_{gk}^{[t-1]}; 0, (\tau_{\beta 1}^{[t-1]})^2) + (1 - p^{[t-1]})\text{N}(\beta_{gk}^{[t-1]}; 0, \tau_{\beta 0}^2)}).$$

5. Update the inclusion probability $p^{[t]}$ for $L_{gk}^{[t]}$s by sampling:

$$p^{[t]} \sim \text{Beta}(\sum_{g=1}^{G}\sum_{k=2}^{K} L_{gk}^{[t]} + a_p, G(K-1) - \sum_{g=1}^{G}\sum_{k=2}^{K} L_{gk}^{[t]} + b_p).$$

6. Update the variance of the spike component of the spike-and-slab prior $(\tau_{\beta 0}^{[t]})^2$ by sampling:

$$(\tau_{\beta 0}^{[t]})^2 \sim \text{Inv-Gamma}(a_\tau + \frac{1}{2}\#\{(g,k) : L_{gk}^{[t]} = 0, 1 \le g \le G, 2 \le k \le K\},$$
$$b_\tau + \frac{1}{2}\sum_{g=1}^{G}\sum_{k=2}^{K} I(L_{gk}^{[t]} = 0) \cdot (\beta_{gk}^{[t-1]})^2),$$

where $\#\{\cdot\}$ represents the number of elements in the set, and $I(\cdot)$ denotes the indicator function.

7. To update $\beta_{gk}^{[t]}$ for cell type two to K and each gene g, we use an MH step with the symmetric proposal distribution $g(\beta_{gk}^*|\beta_{gk}^{[t-1]}) \sim \text{N}(\beta_{gk}^{[t-1]}, \sigma_{\text{MH}}^2)$ and the acceptance rate

$$\rho = \min\{\frac{L(\beta_{gk}^*|-)}{L(\beta_{gk}^{[t-1]}|-)}, 1\}$$
$$= \min\{\prod_{(b,i):w_{bi}^{[t-1]}=k} exp((\beta_{gk}^* - \beta_{gk}^{[t-1]})x_{big}^{[t]})(\frac{\phi_{bg}^{[t-1]} + exp(\alpha_g^{[t]} + \beta_{gk}^{[t-1]} + \nu_{bg}^{[t-1]} + \delta_{bi}^{[t-1]})}{\phi_{bg}^{[t-1]} + exp(\alpha_g^{[t]} + \beta_{gk}^{*} + \nu_{bg}^{[t-1]} + \delta_{bi}^{[t-1]})})^{\phi_{bg}^{[t-1]} + x_{big}^{[t]}}$$
$$\cdot exp(-\frac{(\beta_{gk}^*)^2 - (\beta_{gk}^{[t-1]})^2}{2(\tau_{\beta L_{gk}^{[t]}}^{[t]})^2}), 1\}.$$

8. Update $\nu_{bg}^{[t]}$ by an MH step with the symmetric proposal distribution $g(\nu_{bg}^*|\nu_{bg}^{[t-1]}) \sim$

$\mathrm{N}(\nu_{bg}^{[t-1]}, \sigma^2_{\mathrm{MH}})$ and the acceptance rate

$$\rho = \min\{\frac{L(\nu_{bg}^{*}|-)}{L(\nu_{bg}^{[t-1]}|-)}, 1\}$$
$$= \min\{\prod_{i=1}^{n_b} exp((\nu_{bg}^{*} - \nu_{bg}^{[t-1]})x_{big}^{[t]})(\frac{\phi_{bg}^{[t-1]} + exp(\alpha_g^{[t]} + \beta_{gk}^{[t]} + \nu_{bg}^{[t-1]} + \delta_{bi}^{[t-1]})}{\phi_{bg}^{[t-1]} + exp(\alpha_g^{[t]} + \beta_{gk}^{[t]} + \nu_{bg}^{*} + \delta_{bi}^{[t-1]})})^{\phi_{bg}^{[t-1]} + x_{big}^{[t]}}$$
$$\cdot exp(-\frac{(\nu_{bg}^{*} - m_{bg}^{c})^2 - (\nu_{bg}^{[t-1]} - m_{bg}^{c})^2}{2\sigma_c^2}), 1\}.$$

9. Update $\delta_{bi}^{[t]}$ by an MH step with the symmetric proposal distribution $g(\delta_{bi}^{*}|\delta_{bi}^{[t-1]}) \sim \mathrm{N}(\delta_{bi}^{[t-1]}, \sigma^2_{\mathrm{MH}})$ and the acceptance rate

$$\rho = \min\{\frac{L(\delta_{bi}^{*}|-)}{L(\delta_{bi}^{[t-1]}|-)}, 1\}$$
$$= \min\{\prod_{g=1}^{G} exp((\delta_{bi}^{*} - \delta_{bi}^{[t-1]})x_{big}^{[t]})(\frac{\phi_{bg}^{[t-1]} + exp(\alpha_g^{[t]} + \beta_{gk}^{[t]} + \nu_{bg}^{[t]} + \delta_{bi}^{[t-1]})}{\phi_{bg}^{[t-1]} + exp(\alpha_g^{[t]} + \beta_{gk}^{[t]} + \nu_{bg}^{[t]} + \delta_{bi}^{*})})^{\phi_{bg}^{[t-1]} + x_{big}^{[t]}}$$
$$\cdot exp(-\frac{(\delta_{bi}^{*} - m_{bi}^{d})^2 - (\delta_{bi}^{[t-1]} - m_{bi}^{d})^2}{2\sigma_d^2}), 1\}.$$

10. Update $\phi_{bg}^{[t]}$ by an MH step with the proposal distribution $g(\phi_{bg}^{*}|\phi_{bg}^{[t-1]}) \sim \mathrm{Gamma}(\phi_{bg}^{[t-1]}, 1)$ and the acceptance rate

$$\rho = \min\{\frac{L(\phi_{bg}^{*})g(\phi_{bg}^{[t-1]}|\phi_{bg}^{*})}{L(\phi_{bg}^{[t-1]})g(\phi_{bg}^{*}|\phi_{bg}^{[t-1]})}, 1\}$$
$$= \min\{\prod_{i=1}^{n_b}[\frac{\Gamma(\phi_{bg}^{*} + x_{big}^{[t]})(\phi_{bg}^{*})^{\phi_{bg}^{*}}}{\Gamma(\phi_{bg}^{*})(\phi_{bg}^{*} + \eta_{big}^{[t]})^{\phi_{bg}^{*} + x_{big}^{[t]}}} \cdot \frac{\Gamma(\phi_{bg}^{[t-1]})(\phi_{bg}^{[t-1]} + \eta_{big}^{[t]})^{\phi_{bg}^{[t-1]} + x_{big}^{[t]}}}{\Gamma(\phi_{bg}^{[t-1]} + x_{big}^{[t]})(\phi_{bg}^{[t-1]})^{\phi_{bg}^{[t-1]}}}]$$
$$\cdot \frac{(\phi_{bg}^{*})^{a_\phi - 1}}{(\phi_{bg}^{[t-1]})^{a_\phi - 1}} exp(-b_\phi(\phi_{bg}^{*} - \phi_{bg}^{[t-1]})) \frac{(\phi_{bg}^{[t-1]})^{\phi_{bg}^{*} - 1}\Gamma(\phi_{bg}^{[t-1]})}{(\phi_{bg}^{*})^{\phi_{bg}^{[t-1]} - 1}\Gamma(\phi_{bg}^{*})} exp(\phi_{bg}^{*} - \phi_{bg}^{[t-1]}), 1\},$$

where $\eta_{big}^{[t]} = exp(\alpha_g^{[t]} + \beta_{gw_{bi}^{[t-1]}}^{[t]} + \nu_{bg}^{[t]} + \delta_{bi}^{[t]})$ denotes the mean gene expression level for gene g in cell i of batch b.

11. The conditional posterior distribution for the cell type indicator $w_{bi}^{[t]}$ of cell i in batch b is:

$$Pr(w_{bi}^{[t]} = k|-) \propto \pi_{bk}^{[t-1]} \prod_{g=1}^{G} \frac{exp[(\alpha_g^{[t]} + \beta_{gk}^{[t]} + \nu_{bg}^{[t]} + \delta_{bi}^{[t]})x_{big}^{[t]}]}{(exp(\alpha_g^{[t]} + \beta_{gk}^{[t]} + \nu_{bg}^{[t]} + \delta_{bi}^{[t]}) + \phi_{bg}^{[t]})^{x_{big}^{[t]} + \phi_{bg}^{[t]}}}.$$

We implement an MH step with the symmetric proposal distribution $Pr(w_{bi}^{[t]} = k^*|w_{bi}^{[t-1]} = k) \sim \text{Multinomial}(1; \frac{1}{K}, \cdots, \frac{1}{K})$ and the acceptance rate

$$\begin{aligned}\rho &= \min\{\frac{Pr(w_{bi}^{[t]} = k^*|-)}{Pr(w_{bi}^{[t]} = k|-)}, 1\} \\ &= \min\{\frac{\pi_{bk^*}^{[t-1]}}{\pi_{bk}^{[t-1]}} \prod_{g=1}^{G} exp[(\beta_{gk^*}^{[t]} - \beta_{gk}^{[t]})x_{big}^{[t]}](\frac{exp(\alpha_g^{[t]} + \beta_{gk}^{[t]} + \nu_{bg}^{[t]} + \delta_{bi}^{[t]}) + \phi_{bg}^{[t]}}{exp(\alpha_g^{[t]} + \beta_{gk^*}^{[t]} + \nu_{bg}^{[t]} + \delta_{bi}^{[t]}) + \phi_{bg}^{[t]}})^{x_{big}^{[t]} + \phi_{bg}^{[t]}}, 1\}.\end{aligned}$$

12. Update $\pi_b^{[t]}$ by sampling from the Dirichlet distribution

$$\text{Dir}(\xi + \sum_{i=1}^{n_b} I(w_{bi}^{[t]} = 1), \xi + \sum_{i=1}^{n_b} I(w_{bi}^{[t]} = 2), \cdots, \xi + \sum_{i=1}^{n_b} I(w_{bi}^{[t]} = K)).$$

The Markov chain of the MCMC algorithm can get stuck in the local modes of the posterior distribution for a long period of time. In principle, we can further incorporate the Metropolis coupled MCMC algorithm [3] to jump out of the local modes more easily. In practice, we recommend running multiple chains with different initial values and then choosing the chain that gives the largest value of the observed data likelihood to conduct the posterior inference. According to our experiences, we can usually achieve good posterior estimations with five Markov chains each with a different initial value by randomly sampling a seed from 1 to 10,000.

Supplementary Note 3: Identifiability theorems and proofs

Lemma 1. *Let $\mathcal{F}^G$ be the family of $G(\geq 2)$-dimensional multivariate distribution with the probability mass function for $\boldsymbol{y} = (y_1, \cdots, y_G)$ as*

$$\begin{aligned}f^G(\boldsymbol{y}|\boldsymbol{\gamma}, \boldsymbol{\phi}, \boldsymbol{\mu}) = \prod_{g=1}^{G} \{ & [\frac{1}{1 + exp(\gamma_0 + \gamma_1 y_g)} f_{NB}(y_g; \mu_g, \phi_g)]^{I(y_g > 0)} \\ & \cdot [\sum_{x=1}^{\infty} \frac{exp(\gamma_0 + \gamma_1 x)}{1 + exp(\gamma_0 + \gamma_1 x)} f_{NB}(x; \mu_g, \phi_g) + f_{NB}(0; \mu_g, \phi_g)]^{I(y_g = 0)}\}\end{aligned} \quad (7)$$

such that $\gamma_1 < 0$ and for any two distinct elements $f_{k_1}^G = f^G(\boldsymbol{y}|\boldsymbol{\gamma}_{k_1}, \boldsymbol{\phi}_{k_1}, \boldsymbol{\mu}_{k_1}) \in \mathcal{F}^G, f_{k_2}^G = f^G(\boldsymbol{y}|\boldsymbol{\gamma}_{k_2}, \boldsymbol{\phi}_{k_2}, \boldsymbol{\mu}_{k_2}) \in \mathcal{F}^G$, there exist at least two dimensions g_1 and g_2 with $\mu_{g_1 k_1} \neq \mu_{g_1 k_2}$ and $\mu_{g_2 k_1} \neq \mu_{g_2 k_2}$, then the class of all finite mixtures of $\mathcal{F}^G$ is identifiable (up to label switching).

Proof. We reparameterize (μ_g, ϕ_g) as (p_g, ϕ_g) such that $p_g = \frac{\mu_g}{\mu_g+\phi_g}$ for all $g = 1, 2, \cdots, G$. Consequently, the identifiability with respect to $(\boldsymbol{\gamma}, \boldsymbol{\phi}, \boldsymbol{\mu})$ is equivalent to that with respect to $(\boldsymbol{\gamma}, \boldsymbol{\phi}, \boldsymbol{p})$. With a little bit abuse of notations, we still use $f^G(\boldsymbol{y}|\boldsymbol{\gamma}, \boldsymbol{\phi}, \boldsymbol{p})$ to indicate the probability mass function of the new parameterization hereafter. Suppose that the finite mixture of $\mathcal{F}^G$ is not identifiable, then we have two different representations of the probability mass function $h(\boldsymbol{y})$ of the same finite mixtures:

$$h(\boldsymbol{y}) = \sum_{k=1}^{K} \pi_k f^G(\boldsymbol{y}|\boldsymbol{\gamma}, \boldsymbol{\phi}, \boldsymbol{p}_k) = \sum_{l=1}^{L} \xi_l f^G(\boldsymbol{y}|\boldsymbol{\delta}, \boldsymbol{\psi}, \boldsymbol{r}_l). \tag{8}$$

where the tuples $(\boldsymbol{\gamma}, \boldsymbol{\phi}, \boldsymbol{p}_k)$ for $k = 1, 2, \cdots, K$ are mutually distinct, and so are the tuples $(\boldsymbol{\delta}, \boldsymbol{\psi}, \boldsymbol{r}_l)$ for $l = 1, 2, \cdots, L$.

We define a total ordering $(\succeq)$ of $\mathcal{F}^G$. For $f_1^G, f_2^G \in \mathcal{F}^G$, $f_1^G \succeq f_2^G$ if:

1. there exists a $g \geq 1$ such that for all $j < g$, $p_{j1} = p_{j2}$ and $\phi_{j1} = \phi_{j2}$ but $p_{g1} > p_{g2}$;
2. or there exists a g such that for all $j < g$, $p_{j1} = p_{j2}$ and $\phi_{j1} = \phi_{j2}$ as well as $p_{g1} = p_{g2}$ but $\phi_{g1} > \phi_{g2}$;
3. or $\boldsymbol{p}_1 = \boldsymbol{p}_2$ and $\boldsymbol{\phi}_1 = \boldsymbol{\phi}_2$ but $\gamma_{11} < \gamma_{21}$;
4. or $\boldsymbol{p}_1 = \boldsymbol{p}_2, \boldsymbol{\phi}_1 = \boldsymbol{\phi}_2$ and $\gamma_{11} = \gamma_{21}$ but $\gamma_{10} \leq \gamma_{20}$.

Without loss of generality, we assume that $f^G(\boldsymbol{y}|\boldsymbol{\gamma}, \boldsymbol{\phi}, \boldsymbol{p}_1) \succeq f^G(\boldsymbol{y}|\boldsymbol{\delta}, \boldsymbol{\psi}, \boldsymbol{r}_1)$ and the mixture components on both sides of Equation (8) are ordered:

$$f^G(\boldsymbol{y}|\boldsymbol{\gamma}, \boldsymbol{\phi}, \boldsymbol{p}_1) \succeq f^G(\boldsymbol{y}|\boldsymbol{\gamma}, \boldsymbol{\phi}, \boldsymbol{p}_2) \succeq \cdots \succeq f^G(\boldsymbol{y}|\boldsymbol{\gamma}, \boldsymbol{\phi}, \boldsymbol{p}_K),$$
$$f^G(\boldsymbol{y}|\boldsymbol{\delta}, \boldsymbol{\psi}, \boldsymbol{r}_1) \succeq f^G(\boldsymbol{y}|\boldsymbol{\delta}, \boldsymbol{\psi}, \boldsymbol{r}_2) \succeq \cdots \succeq f^G(\boldsymbol{y}|\boldsymbol{\delta}, \boldsymbol{\psi}, \boldsymbol{r}_L).$$

For $k = 1$, we use mathematical induction to prove that for every $G_0 \in \{1, 2, \cdots, G\}$,

$$r_{j1} = p_{j1}, \phi_j = \psi_j, \forall j \in \{1, 2, \cdots, G_0\}, \tag{9}$$

and there exist a K_{G_0} and an L_{G_0} such that

$$\sum_{k=1}^{K_{G_0}} \pi_k f^{G-G_0}(\boldsymbol{y}_{-G_0}|\boldsymbol{\gamma}, \boldsymbol{\phi}_{-G_0}, \boldsymbol{p}_{-G_0,k}) = \sum_{l=1}^{L_{G_0}} \xi_l f^{G-G_0}(\boldsymbol{y}_{-G_0}|\boldsymbol{\delta}, \boldsymbol{\psi}_{-G_0}, \boldsymbol{r}_{-G_0,l}), \tag{10}$$

where the subscript $-G_0$ denotes that the first G_0 entries in the original vectors are excluded. Specifically, $\boldsymbol{y}_{-G_0} = (y_{G_0+1}, y_{G_0+2}, \cdots, y_G)^T$.

We first prove Equations (9) and (10) hold for $G_0 = 1$. We define a linear mapping that maps a probability distribution of $\mathcal{F}^G$ to a function that shares a similar spirit as a probability generating function $M_1 : f^G(\boldsymbol{y}) \in \mathcal{F}^G \to \Phi_1(t_1, \boldsymbol{y}_{-1}) \in \mathcal{G}_1$ such that $M_1(f^G(\boldsymbol{y})) = \Phi_1(t_1, \boldsymbol{y}_{-1}) = \sum_{y_1=1}^{\infty} f^G(\boldsymbol{y}|\boldsymbol{\gamma}, \boldsymbol{\phi}, \boldsymbol{p}) t_1^{y_1} = \sum_{y_1=1}^{\infty} f^1(y_1|\boldsymbol{\gamma}, \phi_1, p_1) t_1^{y_1} \cdot f^{G-1}(\boldsymbol{y}_{-1}|\boldsymbol{\gamma}, \boldsymbol{\phi}_{-1}, \boldsymbol{p}_{-1})$. Notice that $\Phi_1(t_1, \boldsymbol{y}_{-1})$ does not include the term of $y_1 = 0$, that is, $f^1(0|\boldsymbol{\gamma}, \phi_1, p_1) t_1^0 \cdot f^{G-1}(\boldsymbol{y}_{-1}|\boldsymbol{\gamma}, \boldsymbol{\phi}_{-1}, \boldsymbol{p}_{-1})$. Specifically, we denote $\Phi_{1k}(t_1, \boldsymbol{y}_{-1}) = M_1(f^G(\boldsymbol{y}|\boldsymbol{\gamma}, \boldsymbol{\phi}, \boldsymbol{p}_k))$ and $\Psi_{1l}(t_1, \boldsymbol{y}_{-1}) = M_1(f^G(\boldsymbol{y}|\boldsymbol{\delta}, \boldsymbol{\psi}, \boldsymbol{r}_l)) \in \mathcal{G}_1$ for $k = 1, 2, \cdots, K$ and $l = 1, 2, \cdots, L$. It is noteworthy that M_1 is a linear mapping so that if applying M_1 to both sides of Equation (8), then we have

$$\sum_{k=1}^{K} \pi_k \Phi_{1k}(t_1, \boldsymbol{y}_{-1}) = \sum_{l=1}^{L} \xi_l \Psi_{1l}(t_1, \boldsymbol{y}_{-1}). \tag{11}$$

More specifically,

$$\begin{aligned}\Phi_{1k}(t_1, \boldsymbol{y}_{-1}) &= \sum_{y_1=1}^{\infty} \frac{1}{1 + exp(\gamma_0 + \gamma_1 y_1)} C_{y_1}^{\phi_1 + y_1 - 1} (p_{1k})^{y_1} (1 - p_{1k})^{\phi_1} (t_1)^{y_1} \cdot f^{G-1}(\boldsymbol{y}_{-1}|\boldsymbol{\gamma}, \boldsymbol{\phi}_{-1}, \boldsymbol{p}_{-1,k}) \\ &= [(\frac{1 - p_{1k}}{1 - p_{1k} t_1})^{\phi_1} - R_{1k}(t_1)] \cdot f^{G-1}(\boldsymbol{y}_{-1}|\boldsymbol{\gamma}, \boldsymbol{\phi}_{-1}, \boldsymbol{p}_{-1,k}),\end{aligned} \tag{12}$$

where $R_{1k}(t_1) = (1 - p_{1k})^{\phi_1} + \sum_{y_1=1}^{\infty} \frac{exp(\gamma_0 + \gamma_1 y_1)}{1 + exp(\gamma_0 + \gamma_1 y_1)} C_{y_1}^{\phi_1 + y_1 - 1} (p_{1k} t_1)^{y_1} (1 - p_{1k})^{\phi_1}$ is the residual part. Let $t_1 \to \frac{1}{p_{11}}$, because $\gamma_1 < 0$ so that $\frac{p_{1k} exp(\gamma_1)}{p_{11}} < \frac{p_{1k}}{p_{11}} \leq 1$, we have

$$\begin{aligned}\lim_{t_1 \to \frac{1}{p_{11}}} R_{1k}(t_1) &\leq (1 - p_{1k})^{\phi_1} + \lim_{t_1 \to \frac{1}{p_{11}}} \sum_{y_1=1}^{\infty} exp(\gamma_0 + \gamma_1 y_1) C_{y_1}^{\phi_1 + y_1 - 1} (p_{1k} t_1)^{y_1} (1 - p_{1k})^{\phi_1} \\ &= [1 - exp(\gamma_0)](1 - p_{1k})^{\phi_1} + exp(\gamma_0)(\frac{1 - p_{1k}}{1 - p_{1k} exp(\gamma_1)/p_{11}})^{\phi_1} < \infty,\end{aligned} \tag{13}$$

Similarly, $\Psi_{1l}(t_1, \boldsymbol{y}_{-1}) = \sum_{y_1=1}^{\infty} f^G(\boldsymbol{y}|\boldsymbol{\delta}, \boldsymbol{\psi}, \boldsymbol{r}_l) t_1^{y_1} = [(\frac{1 - r_{1l}}{1 - r_{1l} t_1})^{\psi_1} - S_{1l}(t_1)] \cdot f^{G-1}(\boldsymbol{y}_{-1}|\boldsymbol{\delta}, \boldsymbol{\psi}_{-1}, \boldsymbol{r}_{-1,l})$,

$$\begin{aligned}S_{1l}(t_1) &= (1 - r_{1l})^{\psi_1} + \sum_{y_1=1}^{\infty} exp(\delta_0 + \delta_1 y_1) C_{y_1}^{\psi_1 + y_1 - 1} (r_{1l} t_1)^{y_1} (1 - r_{1l})^{\psi_1} \\ &\leq [1 - exp(\delta_0)](1 - r_{1l})^{\psi_1} + exp(\delta_0)(\frac{1 - r_{1l}}{1 - r_{1l} exp(\delta_1) t_1})^{\psi_1}\end{aligned} \tag{14}$$

As $t_1 \to \frac{1}{p_{11}}$, because $\frac{r_{1l} exp(\gamma_1)}{p_{11}} < \frac{r_{1l}}{p_{11}} \leq \frac{r_{11}}{p_{11}} \leq 1$, we have $\lim_{t_1 \to \frac{1}{p_{11}}} S_{1l}(t_1) < \infty$.
Notice that $f^G(\boldsymbol{y}|\boldsymbol{\gamma}, \boldsymbol{\phi}, \boldsymbol{p}_1) \succeq f^G(\boldsymbol{y}|\boldsymbol{\delta}, \boldsymbol{\psi}, \boldsymbol{r}_1)$ implies $r_{11} < p_{11}$ or $r_{11} = p_{11}, \psi_1 \leq \phi_1$. According

to Equations (13) and (14), we have

$$\begin{aligned}
\lim_{t_1\to\frac{1}{p_{11}}} \frac{\Psi_{1l}(t_1,\boldsymbol{y}_{-1})}{\Phi_{11}(t_1,\boldsymbol{y}_{-1})} &= \lim_{t_1\to\frac{1}{p_{11}}} \frac{(\frac{1-r_{1l}}{1-r_{1l}t_1})^{\psi_1} - S_{1l}(t_1)}{(\frac{1-p_{11}}{1-p_{11}t_1})^{\phi_1} - R_{11}(t_1)} \cdot \frac{f^{G-1}(\boldsymbol{y}_{-1}|\boldsymbol{\delta},\boldsymbol{\psi}_{-1},\boldsymbol{r}_{-1,l})}{f^{G-1}(\boldsymbol{y}_{-1}|\boldsymbol{\gamma},\boldsymbol{\phi}_{-1},\boldsymbol{p}_{-1,1})} \\
&= \frac{f^{G-1}(\boldsymbol{y}_{-1}|\boldsymbol{\delta},\boldsymbol{\psi}_{-1},\boldsymbol{r}_{-1,l})}{f^{G-1}(\boldsymbol{y}_{-1}|\boldsymbol{\gamma},\boldsymbol{\phi}_{-1},\boldsymbol{p}_{-1,1})} \cdot \lim_{t_1\to\frac{1}{p_{11}}} \frac{(\frac{1-r_{1l}}{1-r_{1l}t_1})^{\psi_1}(1-p_{11}t_1)^{\phi_1} - S_{1l}(t_1)(1-p_{11}t_1)^{\phi_1}}{(1-p_{11})^{\phi_1} - R_{11}(t_1)(1-p_{11}t_1)^{\phi_1}} \\
&= \begin{cases} \frac{f^{G-1}(\boldsymbol{y}_{-1}|\boldsymbol{\delta},\boldsymbol{\psi}_{-1},\boldsymbol{r}_{-1,l})}{f^{G-1}(\boldsymbol{y}_{-1}|\boldsymbol{\gamma},\boldsymbol{\phi}_{-1},\boldsymbol{p}_{-1,1})} & , \ if\ r_{1l}=p_{11}, \psi_1=\phi_1 \\ 0 & , \ if\ r_{1l}=p_{11}, \psi_1<\phi_1 \\ 0 & , \ if\ r_{1l}<p_{11} \end{cases} \qquad (15)
\end{aligned}$$

If $r_{11} < p_{11}$ or $r_{11} = p_{11}, \psi_1 < \phi_1$, then dividing $\Phi_{11}(t_1, \boldsymbol{y}_{-1})$ on both sides of Equation (11) and let $t_1 \to \frac{1}{p_{11}}$, we have

$$\lim_{t_1\to\frac{1}{p_{11}}} \sum_{k=1}^{K} \pi_k \frac{\Phi_{1k}(t_1,\boldsymbol{y}_{-1})}{\Phi_{11}(t_1,\boldsymbol{y}_{-1})} \geq \lim_{t_1\to\frac{1}{p_{11}}} \pi_1 \frac{\Phi_{11}(t_1,\boldsymbol{y}_{-1})}{\Phi_{11}(t_1,\boldsymbol{y}_{-1})} = \pi_1 > 0 = \lim_{t_1\to\frac{1}{p_{11}}} \sum_{l=1}^{L} \xi_l \frac{\Psi_{1l}(t_1,\boldsymbol{y}_{-1})}{\Phi_{11}(t_1,\boldsymbol{y}_{-1})},$$

which contradicts with Equation (8). Thus, $r_{11} = p_{11}$ and $\psi_1 = \phi_1$, which means Equation (9) holds. Similar to Equation (15), we have

$$\lim_{t_1\to\frac{1}{p_{11}}} \frac{\Phi_{1k}(t_1,\boldsymbol{y}_{-1})}{\Phi_{11}(t_1,\boldsymbol{y}_{-1})} = \begin{cases} \frac{f^{G-1}(\boldsymbol{y}_{-1}|\boldsymbol{\gamma},\boldsymbol{\phi}_{-1},\boldsymbol{p}_{-1,k})}{f^{G-1}(\boldsymbol{y}_{-1}|\boldsymbol{\gamma},\boldsymbol{\phi}_{-1},\boldsymbol{p}_{-1,1})} & , \ if\ p_{1k}=p_{11} \\ 0 & , \ if\ p_{1k}<p_{11} \end{cases}$$

Moreover, there exists a $K_1 \leq K$ such that $p_{1k} = p_{11}$ for $k = 1, 2, \cdots, K_1$ but $p_{1k} < p_{11}$ for $k = K_1 + 1, \cdots, K$. There also exists an $L_1 \leq L$ such that $r_{1l} = p_{11}$ for $l = 1, 2, \cdots, L_1$ but $r_{1l} < p_{11}$ for $l = L_1 + 1, \cdots, L$. $p_{11} = p_{11}$ and $r_{11} = p_{11}$, therefore, $K_1 \geq 1$ and $L_1 \geq 1$. Dividing $\Phi_{11}(t_1, \boldsymbol{y}_{-1})$ on both sides of Equation (11) and let $t_1 \to \frac{1}{p_{11}}$, we have

$$\begin{aligned}
\sum_{k=1}^{K_1} \pi_k \frac{\Phi_{1k}(t_1,\boldsymbol{y}_{-1})}{\Phi_{11}(t_1,\boldsymbol{y}_{-1})} &= \sum_{l=1}^{L_1} \xi_l \frac{\Psi_{1l}(t_1,\boldsymbol{y}_{-1})}{\Phi_{11}(t_1,\boldsymbol{y}_{-1})} \\
\Rightarrow \sum_{k=1}^{K_1} \pi_k f^{G-1}(\boldsymbol{y}_{-1}|\boldsymbol{\gamma},\boldsymbol{\phi}_{-1},\boldsymbol{p}_{-1,k}) &= \sum_{l=1}^{L_1} \xi_l f^{G-1}(\boldsymbol{y}_{-1}|\boldsymbol{\delta},\boldsymbol{\psi}_{-1},\boldsymbol{r}_{-1,l}). \qquad (16)
\end{aligned}$$

Thus, we have proven that Equation (10) holds.

Now let us assume that Equations (9) and (10) hold for for $G_0 = g$. In other words, $p_{j1} =$

$r_{j1}, \phi_j = \psi_j$ for all $j = 1, 2, \cdots, g$ and there are $K_g \geq 1$ and $L_g \geq 1$ such that

$$\sum_{k=1}^{K_g} \pi_k f^{G-g}(\boldsymbol{y}_{-g}|\boldsymbol{\gamma}, \boldsymbol{\phi}_{-g}, \boldsymbol{p}_{-g,k}) = \sum_{l=1}^{L_g} \xi_l f^{G-g}(\boldsymbol{y}_{-g}|\boldsymbol{\delta}, \boldsymbol{\psi}_{-g}, \boldsymbol{r}_{-g,l}). \tag{17}$$

Let $G_0 = g + 1$. Similar to M_1, we define a linear map $M_{g+1} : \mathcal{F}^{G-g} \to \mathcal{G}_{g+1}$ such that $M_{g+1}(f^{G-g}(\boldsymbol{y}_{-g}|\boldsymbol{\gamma}, \boldsymbol{\phi}_{-g}, \boldsymbol{p}_{-g})) = \Phi_{g+1}(t_{g+1}, \boldsymbol{y}_{-(g+1)}) = \sum_{y_{g+1}=1}^{\infty} f^{G-g}(\boldsymbol{y}_{-g}|\boldsymbol{\gamma}, \boldsymbol{\phi}_{-g}, \boldsymbol{p}_{-g}) t_{g+1}^{y_{g+1}} = \sum_{y_{g+1}=1}^{\infty} f^1(y_{g+1}|\gamma, \phi_{g+1}, p_{g+1}) t_{g+1}^{y_{g+1}} \cdot f^{G-(g+1)}(\boldsymbol{y}_{-(g+1)}|\boldsymbol{\gamma}, \boldsymbol{\phi}_{-(g+1)}, \boldsymbol{p}_{-(g+1)})$. Consequently,

$$\begin{aligned} &M_{g+1}(f^{G-g}(\boldsymbol{y}_{-g}|\boldsymbol{\gamma}, \boldsymbol{\phi}_{-g}, \boldsymbol{p}_{-g,k})) = \Phi_{g+1,k}(t_{g+1}, \boldsymbol{y}_{-(g+1)}) \\ &= [(\frac{1 - p_{g+1,k}}{1 - p_{g+1,k} t_{g+1}})^{\phi_{g+1}} - R_{g+1,k}(t_{g+1})] \cdot f^{G-(g+1)}(\boldsymbol{y}_{-(g+1)}|\boldsymbol{\gamma}, \boldsymbol{\phi}_{-(g+1)}, \boldsymbol{p}_{-(g+1),k}),\ k = 1, \cdots, K_g; \end{aligned}$$

$$\begin{aligned} &M_{g+1}(f^{G-g}(\boldsymbol{y}_{-g}|\boldsymbol{\delta}, \boldsymbol{\psi}_{-g}, \boldsymbol{r}_{-g,l})) = \Psi_{g+1,l}(t_{g+1}, \boldsymbol{y}_{-(g+1)}) \\ &= [(\frac{1 - r_{g+1,l}}{1 - p_{g+1,l} t_{g+1}})^{\psi_{g+1}} - S_{g+1,l}(t_{g+1})] \cdot f^{G-(g+1)}(\boldsymbol{y}_{-(g+1)}|\boldsymbol{\delta}, \boldsymbol{\psi}_{-(g+1)}, \boldsymbol{r}_{-(g+1),l}),\ l = 1, \cdots, L_g. \end{aligned}$$

If we apply M_{g+1} to both sides of Equation (17), then we have

$$\sum_{k=1}^{K_g} \pi_k \Phi_{g+1,k}(t_{g+1}, \boldsymbol{y}_{-(g+1)}) = \sum_{l=1}^{L_g} \xi_l \Psi_{g+1,l}(t_{g+1}, \boldsymbol{y}_{-(g+1)}). \tag{18}$$

Notice that given $p_{j1} = r_{j1}, \phi_j = \psi_j$ for all $j = 1, 2, \cdots, g$, $f^G(\boldsymbol{y}|\boldsymbol{\gamma}, \boldsymbol{\phi}, \boldsymbol{p}_1) \succeq f^G(\boldsymbol{y}|\boldsymbol{\delta}, \boldsymbol{\psi}, \boldsymbol{r}_1)$ implies $r_{g+1,1} < p_{g+1,1}$ or $r_{g+1,1} = p_{g+1,1}, \psi_{g+1} \leq \phi_{g+1}$. Similar to Equation (15), we have

$$\begin{aligned} &\lim_{t_{g+1} \to \frac{1}{p_{g+1,1}}} \frac{\Psi_{g+1,l}(t_{g+1}, \boldsymbol{y}_{-(g+1)})}{\Phi_{g+1,1}(t_{g+1}, \boldsymbol{y}_{-(g+1)})} \\ &= \lim_{t_{g+1} \to \frac{1}{p_{g+1,1}}} \frac{(\frac{1-r_{g+1,l}}{1-r_{g+1,l}t_{g+1}})^{\psi_{g+1}} - S_{g+1,l}(t_{g+1})}{(\frac{1-p_{g+1,1}}{1-p_{g+1,1}t_{g+1}})^{\phi_{g+1}} - R_{g+1,1}(t_{g+1})} \cdot \frac{f^{G-(g+1)}(\boldsymbol{y}_{-(g+1)}|\boldsymbol{\delta}, \boldsymbol{\psi}_{-(g+1)}, \boldsymbol{r}_{-(g+1),l})}{f^{G-(g+1)}(\boldsymbol{y}_{-(g+1)}|\boldsymbol{\gamma}, \boldsymbol{\phi}_{-(g+1)}, \boldsymbol{p}_{-(g+1),1})} \\ &= \frac{f^{G-(g+1)}(\boldsymbol{y}_{-(g+1)}|\boldsymbol{\delta}, \boldsymbol{\psi}_{-(g+1)}, \boldsymbol{r}_{-(g+1),l})}{f^{G-(g+1)}(\boldsymbol{y}_{-(g+1)}|\boldsymbol{\gamma}, \boldsymbol{\phi}_{-(g+1)}, \boldsymbol{p}_{-(g+1),1})} \cdot \lim_{t_{g+1} \to \frac{1}{p_{g+1,1}}} \frac{(\frac{1-r_{g+1,l}}{1-r_{g+1,l}t_{g+1}})^{\psi_{g+1}} - S_{g+1,l}(t_{g+1})}{(\frac{1-p_{g+1,1}}{1-p_{g+1,1}t_{g+1}})^{\phi_{g+1}} - R_{g+1,1}(t_{g+1})} \\ &= \begin{cases} \frac{f^{G-(g+1)}(\boldsymbol{y}_{-(g+1)}|\boldsymbol{\delta}, \boldsymbol{\psi}_{-(g+1)}, \boldsymbol{r}_{-(g+1),l})}{f^{G-(g+1)}(\boldsymbol{y}_{-(g+1)}|\boldsymbol{\gamma}, \boldsymbol{\phi}_{-(g+1)}, \boldsymbol{p}_{-(g+1),1})} & ,\ if\ r_{g+1,l} = p_{g+1,1}, \psi_{g+1} = \phi_{g+1} \\ 0 & ,\ if\ r_{g+1,l} = p_{g+1,1}, \psi_{g+1} < \phi_{g+1} \\ 0 & ,\ if\ r_{g+1,l} < p_{g+1,1} \end{cases} \end{aligned} \tag{19}$$

If $r_{g+1,l} < p_{g+1,1}$ or $r_{g+1,l} = p_{g+1,1}, \psi_{g+1} < \phi_{g+1}$, then dividing $\Phi_{g+1,1}(t_{g+1}, \boldsymbol{y}_{-(g+1)})$ on both sides of Equation (18) and letting $t_{g+1} \to \frac{1}{p_{g+1,1}}$, we have

$$\lim_{t_{g+1}\to\frac{1}{p_{g+1,1}}} \sum_{k=1}^{K_g} \pi_k \frac{\Phi_{g+1,k}(t_{g+1}, \boldsymbol{y}_{-(g+1)})}{\Phi_{g+1,1}(t_{g+1}, \boldsymbol{y}_{-(g+1)})} \geq \lim_{t_{g+1}\to\frac{1}{p_{g+1,1}}} \pi_1 \frac{\Phi_{g+1,1}(t_{g+1}, \boldsymbol{y}_{-(g+1)})}{\Phi_{g+1,1}(t_{g+1}, \boldsymbol{y}_{-(g+1)})} = \pi_1$$

$$> 0 = \lim_{t_{g+1}\to\frac{1}{p_{g+1,1}}} \sum_{l=1}^{L_g} \xi_l \frac{\Psi_{g+1,l}(t_{g+1}, \boldsymbol{y}_{-(g+1)})}{\Phi_{g+1,1}(t_{g+1}, \boldsymbol{y}_{-(g+1)})}, \quad (20)$$

which contradicts with Equation (17). Thus, $r_{g+1,l} = p_{g+1,1}$ and $\psi_{g+1} = \phi_{g+1}$, which means that Equation (9) holds. Similar to Equation (19), for $k = 1, 2, \cdots, K_g$, we have

$$\lim_{t_{g+1}\to\frac{1}{p_{g+1,1}}} \frac{\Phi_{g+1,k}(t_{g+1}, \boldsymbol{y}_{-(g+1)})}{\Phi_{g+1,1}(t_{g+1}, \boldsymbol{y}_{-(g+1)})} = \begin{cases} \frac{f^{G-(g+1)}(\boldsymbol{y}_{-(g+1)}|\boldsymbol{\gamma}, \boldsymbol{\phi}_{-(g+1)}, \boldsymbol{p}_{-(g+1),k})}{f^{G-(g+1)}(\boldsymbol{y}_{-(g+1)}|\boldsymbol{\gamma}, \boldsymbol{\phi}_{-(g+1)}, \boldsymbol{p}_{-(g+1),1})} & , \ if \ p_{g+1,k} = p_{g+1,1} \\ 0 & , \ if \ p_{g+1,k} < p_{g+1,1} \end{cases}$$

Further, there exists a $K_{g+1} \leq K_g$ such that $p_{g+1,k} = p_{g+1,1}$ for $k = 1, 2, \cdots, K_{g+1}$ but $p_{g+1,k} < p_{g+1,1}$ for $k = K_{g+1}+1, K_{g+1}+2, \cdots, K_g$. There also exists an $L_{g+1} \leq L_g$ such that $r_{g+1,l} = p_{g+1,1}$ for $l = 1, 2, \cdots, L_{g+1}$ but $r_{g+1,l} < p_{g+1,1}$ for $l = L_{g+1} + 1, L_{g+1} + 2, \cdots, L_g$. $p_{g+1,1} = p_{g+1,1}$ and $p_{g+1,1} = r_{g+1,1}$, therefore, $K_{g+1} \geq 1$ and $L_{g+1} \geq 1$. Dividing $\Phi_{g+1,1}(t_{g+1}, \boldsymbol{y}_{-(g+1)})$ on both sides of Equation (18) and letting $t_{g+1} \to \frac{1}{p_{g+1,1}}$, we have,

$$\sum_{k=1}^{K_{g+1}} \pi_k \frac{\Phi_{g+1,k}(t_{g+1}, \boldsymbol{y}_{-(g+1)})}{\Phi_{g+1,1}(t_{g+1}, \boldsymbol{y}_{-(g+1)})} = \sum_{l=1}^{L_{g+1}} \xi_l \frac{\Psi_{g+1,l}(t_{g+1}, \boldsymbol{y}_{-(g+1)})}{\Phi_{g+1,1}(t_{g+1}, \boldsymbol{y}_{-(g+1)})}$$

$$\Rightarrow \sum_{k=1}^{K_{g+1}} \pi_k f^{G-(g+1)}(\boldsymbol{y}_{-(g+1)}|\boldsymbol{\gamma}, \boldsymbol{\phi}_{-(g+1)}, \boldsymbol{p}_{-(g+1),k}) = \sum_{l=1}^{L_{g+1}} \xi_l f^{G-(g+1)}(\boldsymbol{y}_{-(g+1)}|\boldsymbol{\delta}, \boldsymbol{\psi}_{-(g+1)}, \boldsymbol{r}_{-(g+1),l}),$$

so Equation (10) holds for $G_0 = g + 1$.

Consequently, by mathematical induction, we have shown that Equations (9) and (10) hold for any $G_0 \in \{1, \cdots, G\}$, which implies that $\boldsymbol{p}_1 = \boldsymbol{r}_1$ and $\boldsymbol{\phi} = \boldsymbol{\psi}$.

For $G_0 = G$ and $G_0 = G - 1$, Equation (10) gives

$$\sum_{k=1}^{K_G} \pi_k = \sum_{l=1}^{L_G} \xi_l, \quad (21)$$

$$\sum_{k=1}^{K_{G-1}} \pi_k f^1(y_G|\boldsymbol{\gamma}, \phi_G, p_{Gk}) = \sum_{l=1}^{L_{G-1}} \xi_l f^1(y_G|\boldsymbol{\delta}, \phi_G, r_{Gl}). \quad (22)$$

For any two distinct elements $f^G(\boldsymbol{y}|\boldsymbol{\gamma},\boldsymbol{\phi},\boldsymbol{p}_1)$ and $f^G(\boldsymbol{y}|\boldsymbol{\gamma},\boldsymbol{\phi},\boldsymbol{p}_k), k=2,3,\cdots,K_{G-2}$, because there exist at least two different dimensions and $p_{g1}=p_{gk}$ with $g=1,2,\cdots,G-2$, $p_{G-1,k}\neq p_{G-1,1}$ and $p_{Gk}\neq p_{G1}$. Therefore, $K_G=K_{G-1}=1$. Similarly, we have $L_G=L_{G-1}=1$. Thus, Equations (21) and (22) turn to

$$\begin{aligned}\pi_1 &= \xi_1\\ f^1(y_G|\boldsymbol{\gamma},\phi_G,p_{G1}) &= f^1(y_G|\boldsymbol{\delta},\phi_G,r_{G1}), \forall y_G\in\mathbb{N}.\end{aligned} \tag{23}$$

Plugging $y_G=1$ and $y_G=2$ into Equation (23), we have

$$\begin{aligned}\frac{1}{1+exp(\gamma_0+\gamma_1)}C_1^{\phi_G}p_{G1}(1-p_{G1})^{\phi_G} &= \frac{1}{1+exp(\delta_0+\delta_1)}C_1^{\phi_G}p_{G1}(1-p_{G1})^{\phi_G}\\ \frac{1}{1+exp(\gamma_0+2\gamma_1)}C_2^{\phi_G+1}p_{G1}^2(1-p_{G1})^{\phi_G} &= \frac{1}{1+exp(\delta_0+2\delta_1)}C_2^{\phi_G+1}p_{G1}^2(1-p_{G1})^{\phi_G},\end{aligned}$$

therefore $\boldsymbol{\gamma}=\boldsymbol{\delta}$.

Plugging $\boldsymbol{\gamma}=\boldsymbol{\delta},\boldsymbol{\phi}=\boldsymbol{\psi},\boldsymbol{p}_1=\boldsymbol{r}_1$ and $\pi_1=\xi_1$ into Equation (8), we have

$$\sum_{k=2}^{K}\pi_k f^G(\boldsymbol{y}|\boldsymbol{\gamma},\boldsymbol{\phi},\boldsymbol{p}_k)=\sum_{l=2}^{L}\xi_l f^G(\boldsymbol{y}|\boldsymbol{\gamma},\boldsymbol{\phi},\boldsymbol{r}_l) \tag{24}$$

Similarly, we can apply mathematical induction to prove that $\boldsymbol{p}_k=\boldsymbol{r}_k$ and $\pi_k=\xi_k$ sequentially for $k=2,3,\cdots,\min\{K,L\}$. Finally, if $K\neq L$, without loss of generality, let us assume that $K>L$, then $\sum_{k=L+1}^{K}\pi_k=1-\sum_{k=1}^{L}\pi_k=1-\sum_{l=1}^{L}\xi_l=0$, which contradicts with $\pi_k>0$ for all $k=1,2,\cdots,K$. Thus, $K=L,\boldsymbol{\gamma}=\boldsymbol{\delta},\boldsymbol{\phi}=\boldsymbol{\psi},\boldsymbol{\pi}=\boldsymbol{\xi}$ and $\boldsymbol{p}_k=\boldsymbol{r}_k$ for all $k=1,2,\cdots,K$. Therefore, the class of all finite mixtures of $\mathcal{F}^G$ is identifiable. □

Theorem 1. *(The Complete Setting)*
If $\pi_{bk}>0$ for every batch b and cell type k, given that (I) $\gamma_{b1}<0$ for every b, (II) for any two cell types k_1 and k_2, there exist at least two differentially expressed genes g_1 and g_2— $\beta_{g_1k_1}\neq\beta_{g_1k_2}$ and $\beta_{g_2k_1}\neq\beta_{g_2k_2}$, and (III) for any two distinct cell-type pairs $(k_1,k_2)\neq(k_3,k_4)$, their differences in cell-type effects are not the same $\boldsymbol{\beta}_{k_1}-\boldsymbol{\beta}_{k_2}\neq\boldsymbol{\beta}_{k_3}-\boldsymbol{\beta}_{k_4}$, then BUSseq is identifiable (up to label switching) in the sense that $L_o(\boldsymbol{\Theta}|\boldsymbol{y})=L_o(\boldsymbol{\Theta}^*|\boldsymbol{y})$ for any $\boldsymbol{y}$ implies that $\pi_{bk}=\pi^*_{b\rho(k)},(\gamma_{b0},\gamma_{b1})=(\gamma^*_{b0},\gamma^*_{b1}),\alpha_g+\beta_{gk}=\alpha^*_g+\beta^*_{g\rho(k)},\nu_{gb}=\nu^*_{gb},\delta_{bi}=\delta^*_{bi}$ and $\phi_{bg}=\phi^*_{bg}$ for every gene g and batch b, where ρ is a permutation of $\{1,2,\cdots,K\}$.

Proof. Let $\boldsymbol{Y}_b\in N^{n_b\times G}$ denote the data from batch b and collect $\boldsymbol{Y}=\{\boldsymbol{Y}_b,1\leq b\leq B\}$ and $\boldsymbol{m}_{bik}=exp(\boldsymbol{\alpha}+\boldsymbol{\beta}_k+\boldsymbol{\nu}_b+\delta_{bi}\mathbf{1})$, then the marginal distribution for $f(\boldsymbol{Y}_b|\boldsymbol{\Theta})=\prod_{i=1}^{n_b}[\sum_{k=1}^{K}\pi_{bk}f^G(\boldsymbol{y}_b|\gamma_b,\boldsymbol{\phi}_b,\boldsymbol{m}_{bik})]$ with $f^G(\boldsymbol{y}_b|\gamma_b,\boldsymbol{\phi}_b,\boldsymbol{m}_{bik})\in\mathcal{F}^G$ reduces to a mixture of G-dimensional zero-inflated negative binomial (ZINB) model on batch b. Therefore, we can view the BUSseq model as a combination of B ZINB models with the constraints that $\boldsymbol{\beta}_k^{(1)}=\cdots=\boldsymbol{\beta}_k^{(B)}=\boldsymbol{\beta}_k$ for each k and $\boldsymbol{\alpha}^{(1)}=\cdots=\boldsymbol{\alpha}^{(B)}=\boldsymbol{\alpha}$.

According to conditions (I)-(III), Lemma 1 and Teicher [4], the ZINB model for batch b is identifiable up to label switching in the sense that $f(\boldsymbol{Y}_b|\boldsymbol{\Theta}) = f(\boldsymbol{Y}_b|\boldsymbol{\Theta}^*)$ for any $\boldsymbol{Y}_b$ implies that $\pi_{bk} = \pi^*_{b\rho_b(k)}, \gamma_b = \boldsymbol{\gamma}^*_b, \boldsymbol{\alpha} + \boldsymbol{\beta}_k + \boldsymbol{\nu}_b + \delta_{bi}\mathbf{1} = log(\boldsymbol{m}_{bik}) = log(\boldsymbol{m}^*_{bi\rho_b(k)}) = \boldsymbol{\alpha}^* + \boldsymbol{\beta}^*_{\rho_b(k)} + \boldsymbol{\nu}^*_b + \delta^*_{bi}\mathbf{1}$ and $\boldsymbol{\phi}_b = \boldsymbol{\phi}^*_b$ for a permutation ρ_b of $\{1, 2, \cdots, K\}$, where $\mathbf{1}$ denotes a vector of one with length G.

We first prove that the permutation ρ_b is the same for all of the batches. Recall that we take the first cell type as the reference cell type with $\boldsymbol{\beta}_1 = 0$. Therefore, the ratio of mean expression levels between cell type k and cell type one is

$$\frac{\boldsymbol{m}_{bik}}{\boldsymbol{m}_{bi1}} = \frac{\boldsymbol{m}^*_{bi\rho_b(k)}}{\boldsymbol{m}^*_{bi\rho_b(1)}} \Rightarrow exp(\boldsymbol{\beta}_k) = exp(\boldsymbol{\beta}^*_{\rho_b(k)} - \boldsymbol{\beta}^*_{\rho_b(1)}) \tag{25}$$

Notice the left hand side of Equation (25) is invariant to the batch indicator b, and therefore $\boldsymbol{\beta}^*_{\rho_b(k)} - \boldsymbol{\beta}^*_{\rho_b(1)} = \boldsymbol{\beta}^*_{\rho_1(k)} - \boldsymbol{\beta}^*_{\rho_1(1)}$ for every k. By condition (III), $\rho_b = \rho_1$ for every b.

Let us then compare $log(\boldsymbol{m}_{b1k})$ with $log(\boldsymbol{m}_{11k})$. Because $\boldsymbol{\nu}_1 = \boldsymbol{\nu}^*_1 = \mathbf{0}$ and $\delta_{b1} = \delta^*_{b1} = 0$, we have

$$\boldsymbol{\alpha} + \boldsymbol{\beta}_k = \boldsymbol{\alpha}^* + \boldsymbol{\beta}^*_{\rho(k)}, \boldsymbol{\alpha} + \boldsymbol{\beta}_k + \boldsymbol{\nu}_b = \boldsymbol{\alpha}^* + \boldsymbol{\beta}^*_{\rho(k)} + \boldsymbol{\nu}^*_b. \tag{26}$$

Thus, we have proven $\boldsymbol{\nu}_b = \boldsymbol{\nu}^*_b$.

Next we compare $log(\boldsymbol{m}_{bik})$ with $log(\boldsymbol{m}_{b1k})$ for each batch. Then, we have

$$\boldsymbol{\alpha} + \boldsymbol{\beta}_k + \boldsymbol{\nu}_b = \boldsymbol{\alpha}^* + \boldsymbol{\beta}^*_{\rho(k)} + \boldsymbol{\nu}_b, \boldsymbol{\alpha} + \boldsymbol{\beta}_k + \boldsymbol{\nu}_b + \delta_{bi}\mathbf{1} = \boldsymbol{\alpha}^* + \boldsymbol{\beta}^*_{\rho(k)} + \boldsymbol{\nu}_b + \delta^*_{bi}\mathbf{1}. \tag{27}$$

Consequently, $\delta_{bi} = \delta^*_{bi}$ for any cell i in any batch. Therefore, BUSseq is identifiable (up to label switching). □

Theorem 2. *(The Reference Panel Design)*
If there are a total of K cell types $\cup_{b=1}^B C_b = \{1, 2, \cdots, K\}$, where C_b denotes the cell types that are present in batch b, the number of cell types existing in batch b $K_b = |C_b| \geq 2$ for every batch b, and there exists a batch $\tilde{b}$ such that it contains all of the cell types $C_{\tilde{b}} = \{1, 2, \cdots, K\}$, then given that conditions (I)-(III) hold, BUSseq is identifiable (up to label switching).

Proof. In the reference panel design, any batch b shares at least two cell types with the first batch. If we compare the two distinct cell types k_1 and k_2 shared by batch b and batch one in terms of the log-scale mean expression levels, respectively, then we have

$$\left.\begin{array}{l} \boldsymbol{\alpha} + \boldsymbol{\beta}_{k_1} + \boldsymbol{\nu}_b + \delta_{bi}\mathbf{1} = \boldsymbol{\alpha}^* + \boldsymbol{\beta}^*_{\rho_b(k_1)} + \boldsymbol{\nu}^*_b + \delta^*_{bi}\mathbf{1}, \\ \boldsymbol{\alpha} + \boldsymbol{\beta}_{k_2} + \boldsymbol{\nu}_b + \delta_{bi}\mathbf{1} = \boldsymbol{\alpha}^* + \boldsymbol{\beta}^*_{\rho_b(k_2)} + \boldsymbol{\nu}^*_b + \delta^*_{bi}\mathbf{1}, \end{array}\right\} \Rightarrow \boldsymbol{\beta}_{k_1} - \boldsymbol{\beta}_{k_2} = \boldsymbol{\beta}^*_{\rho_b(k_1)} - \boldsymbol{\beta}^*_{\rho_b(k_2)},$$

$$\left.\begin{array}{l}\boldsymbol{\alpha}+\boldsymbol{\beta}_{k_1}+\delta_{1i}\mathbf{1}=\boldsymbol{\alpha}^*+\boldsymbol{\beta}^*_{\rho_1(k_1)}+\delta^*_{1i}\mathbf{1},\\ \boldsymbol{\alpha}+\boldsymbol{\beta}_{k_2}+\delta_{1i}\mathbf{1}=\boldsymbol{\alpha}^*+\boldsymbol{\beta}^*_{\rho_1(k_2)}+\delta^*_{1i}\mathbf{1}.\end{array}\right\}\Rightarrow\boldsymbol{\beta}_{k_1}-\boldsymbol{\beta}_{k_2}=\boldsymbol{\beta}^*_{\rho_1(k_1)}-\boldsymbol{\beta}^*_{\rho_1(k_2)}.$$

Further, according to condition (III), $\boldsymbol{\beta}_{k_1}-\boldsymbol{\beta}_{k_2}=\boldsymbol{\beta}^*_{\rho_b(k_1)}-\boldsymbol{\beta}^*_{\rho_b(k_2)}=\boldsymbol{\beta}^*_{\rho_1(k_1)}-\boldsymbol{\beta}^*_{\rho_1(k_2)}$ implies that $\rho_b(k)=\rho_1(k)$ for each cell type $k\in C_b$ ($b\geq 2$).

Finally, similar to Equations (26) and (27), for a shared cell type k between batch b and batch one, we have

$$\begin{aligned}\boldsymbol{\alpha}+\boldsymbol{\beta}_k&=\boldsymbol{\alpha}^*+\boldsymbol{\beta}^*_{\rho_1(k)}\\ \boldsymbol{\alpha}+\boldsymbol{\beta}_k+\boldsymbol{\nu}_b&=\boldsymbol{\alpha}^*+\boldsymbol{\beta}^*_{\rho_b(k)}+\boldsymbol{\nu}^*_b\\ \boldsymbol{\alpha}+\boldsymbol{\beta}_k+\boldsymbol{\nu}_b+\delta_{bi}\mathbf{1}&=\boldsymbol{\alpha}^*+\boldsymbol{\beta}^*_{\rho_b(k)}+\boldsymbol{\nu}^*_b+\delta^*_{bi}\mathbf{1}.\end{aligned}$$

Thus, $\rho_b(k)=\rho_1(k)$ for each $k\in C_b$ ($b\geq 2$) implies that $\boldsymbol{\nu}_b=\boldsymbol{\nu}^*_b$ and $\delta_{bi}=\delta^*_{bi}$. □

Theorem 3. *(The Chain-type Design)*
If there are a total of K cell types $\cup_{b=1}^B C_b=\{1,2,\cdots,K\}$ and every two consecutive batches share at least two cell types $|C_b\cap C_{b-1}|\geq 2$ for all $b\geq 2$, then given that conditions (I)-(III) hold, BUSseq is identifiable (up to label switching).

Proof. Our objective is to prove that for any two distinct batches b and $\tilde{b}$, $\rho_b(k)=\rho_{\tilde{b}}(k)$ holds for any cell type $k\in C_b\bigcap C_{\tilde{b}}$ shared by these two batches.

First, we prove that $\rho_b(k)=\rho_{b-1}(k)$ for the shared cell types $k\in C_b\bigcap C_{b-1}, 2\leq b\leq B$ in any two consecutive batches. Notice that $|C_b\cap C_{b-1}|\geq 2$, so for any two shared cell types k_1 and k_2 between batch b and batch $b-1$, we have

$$\left.\begin{array}{l}\boldsymbol{\alpha}+\boldsymbol{\beta}_{k_1}+\boldsymbol{\nu}_b+\delta_{bi}\mathbf{1}=\boldsymbol{\alpha}^*+\boldsymbol{\beta}^*_{\rho_b(k_1)}+\boldsymbol{\nu}^*_b+\delta^*_{bi}\mathbf{1},\\ \boldsymbol{\alpha}+\boldsymbol{\beta}_{k_2}+\boldsymbol{\nu}_b+\delta_{bi}\mathbf{1}=\boldsymbol{\alpha}^*+\boldsymbol{\beta}^*_{\rho_b(k_2)}+\boldsymbol{\nu}^*_b+\delta^*_{bi}\mathbf{1},\end{array}\right\}\Rightarrow\boldsymbol{\beta}_{k_1}-\boldsymbol{\beta}_{k_2}=\boldsymbol{\beta}^*_{\rho_b(k_1)}-\boldsymbol{\beta}^*_{\rho_b(k_2)}$$

$$\left.\begin{array}{l}\boldsymbol{\alpha}+\boldsymbol{\beta}_{k_1}+\boldsymbol{\nu}_{b-1}+\delta_{b-1,i}\mathbf{1}=\boldsymbol{\alpha}^*+\boldsymbol{\beta}^*_{\rho_{b-1}(k_1)}+\boldsymbol{\nu}^*_{b-1}+\delta^*_{b-1,i}\mathbf{1},\\ \boldsymbol{\alpha}+\boldsymbol{\beta}_{k_2}+\boldsymbol{\nu}_{b-1}+\delta_{b-1,i}\mathbf{1}=\boldsymbol{\alpha}^*+\boldsymbol{\beta}^*_{\rho_{b-1}(k_2)}+\boldsymbol{\nu}^*_{b-1}+\delta^*_{b-1,i}\mathbf{1}.\end{array}\right\}\Rightarrow\boldsymbol{\beta}_{k_1}-\boldsymbol{\beta}_{k_2}=\boldsymbol{\beta}^*_{\rho_{b-1}(k_1)}-\boldsymbol{\beta}^*_{\rho_{b-1}(k_2)}$$

Further, according to condition (III), $\boldsymbol{\beta}_{k_1}-\boldsymbol{\beta}_{k_2}=\boldsymbol{\beta}^*_{\rho_b(k_1)}-\boldsymbol{\beta}^*_{\rho_b(k_2)}=\boldsymbol{\beta}^*_{\rho_{b-1}(k_1)}-\boldsymbol{\beta}^*_{\rho_{b-1}(k_2)}$ implies that $\rho_b(k)=\rho_{b-1}(k)$ for $2\leq b\leq B, k\in C_b\bigcap C_{b-1}$.

Consequently, for a cell type $k\in C_b\bigcap C_{b-1}$ shared by two consecutive batches b and $b-1$, similar to Equation (26), we have

$$\left.\begin{array}{l}\boldsymbol{\alpha}+\boldsymbol{\beta}_k+\boldsymbol{\nu}_{b-1}=\boldsymbol{\alpha}^*+\boldsymbol{\beta}^*_{\rho_{b-1}(k)}+\boldsymbol{\nu}^*_{b-1}\\ \boldsymbol{\alpha}+\boldsymbol{\beta}_k+\boldsymbol{\nu}_b=\boldsymbol{\alpha}^*+\boldsymbol{\beta}^*_{\rho_b(k)}+\boldsymbol{\nu}^*_b\end{array}\right\}\Rightarrow\boldsymbol{\nu}_b-\boldsymbol{\nu}_{b-1}=\boldsymbol{\nu}^*_b-\boldsymbol{\nu}^*_{b-1}.$$

Because $\boldsymbol{\nu}_1=\boldsymbol{\nu}^*_1=0$, $\boldsymbol{\nu}_b=\sum_{j=2}^{b}(\boldsymbol{\nu}_j-\boldsymbol{\nu}_{j-1})=\sum_{j=2}^{b}(\boldsymbol{\nu}^*_j-\boldsymbol{\nu}^*_{j-1})=\boldsymbol{\nu}^*_b$. Moreover, similar to Equation (27), we have $\delta_{bi}=\delta^*_{bi}$ for each cell i of each batch b.

Now for any two distinct batches b and $\tilde{b}$, we can directly compare the mean expression levels of their shared cell type $k\in C_b\bigcap C_{\tilde{b}}$:

$$\left.\begin{array}{l}\boldsymbol{\alpha}+\boldsymbol{\beta}_k+\boldsymbol{\nu}_b+\delta_{bi}\mathbf{1}=\boldsymbol{\alpha}^*+\boldsymbol{\beta}^*_{\rho_b(k)}+\boldsymbol{\nu}_b+\delta_{bi}\mathbf{1}\\ \boldsymbol{\alpha}+\boldsymbol{\beta}_k+\boldsymbol{\nu}_{\tilde{b}}+\delta_{\tilde{b}i}\mathbf{1}=\boldsymbol{\alpha}^*+\boldsymbol{\beta}^*_{\rho_{\tilde{b}}(k)}+\boldsymbol{\nu}_{\tilde{b}}+\delta_{\tilde{b}i}\mathbf{1}\end{array}\right\}\Rightarrow\boldsymbol{\beta}^*_{\rho_b(k)}=\boldsymbol{\beta}^*_{\rho_{\tilde{b}}(k)}$$

□

Theorem 4. *(The Connected Design)*
We define a batch graph $G=(V,E)$. Each node $b\in V$ represents a batch. There is an edge $e\in E$ between two nodes b_1 and b_2 if and only if batches b_1 and b_2 share at least two cell types. If the batch graph is connected and conditions (I)-(III) hold, then BUSseq is identifiable (up to label switching).

Proof. Our object is to prove that for any two distinct batches b and $\tilde{b}$, $\rho_b(k)=\rho_{\tilde{b}}(k)$ holds for any cell type $k\in C_b\bigcap C_{\tilde{b}}$ shared by these two batches. At the same time, $\boldsymbol{\nu}_b=\boldsymbol{\nu}^*_b$ and $\delta_{bi}=\delta^*_{bi}$ for each cell i in batch b.

For any two connected batches (b_1,b_2), we have $|C_{b_1}\cap C_{b_2}|\geq 2$. Thus, for any two shared cell types $k_1,k_2\in C_{b_1}\cap C_{b_2}$, we have

$$\left.\begin{array}{l}\boldsymbol{\alpha}+\boldsymbol{\beta}_{k_1}+\boldsymbol{\nu}_{b_1}+\delta_{b_1,i}\mathbf{1}=\boldsymbol{\alpha}^*+\boldsymbol{\beta}^*_{\rho_{b_1}(k_1)}+\boldsymbol{\nu}^*_{b_1}+\delta^*_{b_1,i}\mathbf{1},\\ \boldsymbol{\alpha}+\boldsymbol{\beta}_{k_2}+\boldsymbol{\nu}_{b_1}+\delta_{b_1,i}\mathbf{1}=\boldsymbol{\alpha}^*+\boldsymbol{\beta}^*_{\rho_{b_1}(k_2)}+\boldsymbol{\nu}^*_{b_1}+\delta^*_{b_1,i}\mathbf{1},\end{array}\right\}\Rightarrow\boldsymbol{\beta}_{k_1}-\boldsymbol{\beta}_{k_2}=\boldsymbol{\beta}^*_{\rho_{b_1}(k_1)}-\boldsymbol{\beta}^*_{\rho_{b_1}(k_2)}$$

$$\left.\begin{array}{l}\boldsymbol{\alpha}+\boldsymbol{\beta}_{k_1}+\boldsymbol{\nu}_{b_2}+\delta_{b_2,i}\mathbf{1}=\boldsymbol{\alpha}^*+\boldsymbol{\beta}^*_{\rho_{b_2}(k_1)}+\boldsymbol{\nu}^*_{b_2}+\delta^*_{b_2,i}\mathbf{1},\\ \boldsymbol{\alpha}+\boldsymbol{\beta}_{k_2}+\boldsymbol{\nu}_{b_2}+\delta_{b_2,i}\mathbf{1}=\boldsymbol{\alpha}^*+\boldsymbol{\beta}^*_{\rho_{b_2}(k_2)}+\boldsymbol{\nu}^*_{b_2}+\delta^*_{b_2,i}\mathbf{1}.\end{array}\right\}\Rightarrow\boldsymbol{\beta}_{k_1}-\boldsymbol{\beta}_{k_2}=\boldsymbol{\beta}^*_{\rho_{b_2}(k_1)}-\boldsymbol{\beta}^*_{\rho_{b_2}(k_2)}$$

Further, according to condition (III), $\boldsymbol{\beta}_{k_1}-\boldsymbol{\beta}_{k_2}=\boldsymbol{\beta}^*_{\rho_{b_1}(k_1)}-\boldsymbol{\beta}^*_{\rho_{b_1}(k_2)}=\boldsymbol{\beta}^*_{\rho_{b_2}(k_1)}-\boldsymbol{\beta}^*_{\rho_{b_2}(k_2)}$ implies that $\rho_{b_1}(k)=\rho_{b_2}(k)$ for $k\in C_{b_1}\bigcap C_{b_2}$.

Consequently, for a cell type $k\in C_{b_1}\bigcap C_{b_2}$ shared by two connected batches b_1 and b_2, similar

to Equation (26), we have

$$\left.\begin{aligned}\boldsymbol{\alpha}+\boldsymbol{\beta}_k+\boldsymbol{\nu}_{b_1}&=\boldsymbol{\alpha}^*+\boldsymbol{\beta}^*_{\rho_{b_1}(k)}+\boldsymbol{\nu}^*_{b_1}\\ \boldsymbol{\alpha}+\boldsymbol{\beta}_k+\boldsymbol{\nu}_{b_2}&=\boldsymbol{\alpha}^*+\boldsymbol{\beta}^*_{\rho_{b_2}(k)}+\boldsymbol{\nu}^*_{b_2}\end{aligned}\right\}\Rightarrow \boldsymbol{\nu}_{b_1}-\boldsymbol{\nu}^*_{b_1}=\boldsymbol{\nu}_{b_2}-\boldsymbol{\nu}^*_{b_2}.$$

Because of the connectivity of the batch graph G, we can find a path $(1, b_1, b_2, \cdots, b_k, b), k \leq B-2$ between any batch b and the first batch in the batch graph G such that $\boldsymbol{\nu}_b - \boldsymbol{\nu}^*_b = \boldsymbol{\nu}_{b_k} - \boldsymbol{\nu}^*_{b_k} = \cdots = \boldsymbol{\nu}_{b_1} - \boldsymbol{\nu}^*_{b_1} = \boldsymbol{\nu}_1 - \boldsymbol{\nu}^*_1$. Notice that $\boldsymbol{\nu}_1 = \boldsymbol{\nu}^*_1 = 0$, so we have $\boldsymbol{\nu}_b = \boldsymbol{\nu}^*_b$. Moreover, similar to Equation (27), we have $\delta_{bi} = \delta^*_{bi}$ for each cell i in the batch b.

Now for any two distinct batches b and $\tilde{b}$, we can directly compare the mean expression levels of their shared cell type $k \in C_b \bigcap C_{\tilde{b}}$:

$$\left.\begin{aligned}\boldsymbol{\alpha}+\boldsymbol{\beta}_k+\boldsymbol{\nu}_b+\delta_{bi}\mathbf{1}&=\boldsymbol{\alpha}^*+\boldsymbol{\beta}^*_{\rho_b(k)}+\boldsymbol{\nu}_b+\delta_{bi}\mathbf{1}\\ \boldsymbol{\alpha}+\boldsymbol{\beta}_k+\boldsymbol{\nu}_{\tilde{b}}+\delta_{\tilde{b}i}\mathbf{1}&=\boldsymbol{\alpha}^*+\boldsymbol{\beta}^*_{\rho_{\tilde{b}}(k)}+\boldsymbol{\nu}_{\tilde{b}}+\delta_{\tilde{b}i}\mathbf{1}\end{aligned}\right\}\Rightarrow \boldsymbol{\beta}^*_{\rho_b(k)}=\boldsymbol{\beta}^*_{\rho_{\tilde{b}}(k)},$$

which implies that $\rho_b(k) = \rho_{\tilde{b}}(k)$ according to condition (II). □

Supplementary Note 4: Benchmarked normalization methods

Lun et al. [5] proposed a deconvolution normalization method and compared its performance with that of five existing normalization methods, including DESeq normalization [6], trimmed mean of M-values (TMM) normalization [7] and library size normalization. The authors drew a scatter plot of the estimated values by each normalization method against the true cell size factors and evaluated how close these scattered points were to the diagonal.

We compared the performance of the above methods with the size factor estimated by BUSseq on the simulation dataset, as the "golden truth" of parameters and latent variables is only available in the simulation study. Moreover, to avoid the impact of batch effects, we focused on the first batch with b = 1. Following Lun et al. [5], we applied the $estimateSizeFactorsForMatrix$ function of the R package $DESeq2$ with the option $geoMeans = gm$ for DESeq normalization, the $calcNormFactors$ function of the R package $edgeR$ for TMM normalization and directly calculated the total read count of each cell as the size factor for library size normalization. For the deconvolution method, we ran the $computeSumFactors$ function with and without prior clustering, respectively, using the R package $scran$. In BUSseq, the estimate of the size factor is the exponential of the posterior mean $exp(\hat{\delta}_{1i})$. **Supplementary Fig. 4** shows that BUSseq correctly estimates the cell size factors and outperforms all existing methods.

Supplementary Note 5: Benchmarked imputation methods

Andrews et al. [8] compared six different imputation methods—SAVER [9], DrImpute [10], scImpute [11], dca [12], MAGIC [13] and knn-smooth [14]. Only the first three imputation

methods use models to distinguish true biological zeros from zero induced by the dropout events. Therefore, we compared the first three methods with BUSseq in terms of the Euclidean distance between the imputed values and true values for all of the observed zero values and the accuracy in recovering the rates of biological zeros. We continue to use the first batch of our simulation dataset so that the true biological zero rate can be calculated for the underlying read count data x_{big}.

Following Andrews et al. [8], we first conducted library-size normalization and then applied SAVER to the normalized data. We applied scImpute to the raw observed count data and set the dropout threshold option as 0.5 and the number of clusters as the true value four. We took the natural logarithm of the normalized data plus one and applied DrImpute to the log-scale data with the number of clusters set to four.

For comparison, we first calculated the Euclidean distance between the imputed values and the underlying true read counts x_{big}s for all of the observed zeros. Notably, however, only scImpute and BUSseq worked on the raw count data. Therefore, to be fair, we standardized the imputed values of SAVER by multiplying the size factor of each cell back to obtain $\widehat{x}_{1ig}^{imputed}$. Similarity, we exponentiated the imputed values output by DrImputed, subtracted one, and finally multiplied the resulting values by the size factor of each cell for standardization to get $\widehat{x}_{1ig}^{imputed}$. Finally, we calculated the Euclidean distance d between x_{1ig} and $\widehat{x}_{1ig}^{imputed}$ for all of the observed zeros:

$$d = \sum_{i=1}^{n_1} \sum_{g=1}^{G} I(y_{1ig} = 0) \sqrt{[log(1 + x_{1ig}) - log(1 + \widehat{x}_{1ig}^{imputed})]^2},$$

where n_1 denotes the number of cells in the first batch, x_{1ig} is the underlying true read count of gene g for cell i in the first batch and $\widehat{x}_{1ig}^{imputed}$ represents the (standardized) imputed values. We measured distance on the log-scale to eliminate the impact of outliers.

We further compared the zero rates of the imputed values. As the three compared methods generated continuous values, we rounded the (standardized) imputed values to imputed read counts and then calculated the zero rates.

According to **Supplementary Tab. 2**, the read counts imputed by BUSseq are closest to the true values in terms of both the Euclidean distance and the rates of biological zeros.

Supplementary Note 6: Sensitivity analyses

Specification of hyper-parameters

We first investigated how hyperparameters of the prior distributions in the Bayesian analysis affect the posterior statistical inference in the simulation study. For each hyperparameter or each

pair of hyperparameters in **Supplementary Tab. 3**, we varied its value(s) to four different levels while fixing the other hyperparameters. Next, we applied BUSseq to each setting and calculated the corresponding ARI to evaluate the clustering accuracy. If the ARI is equal to one, all of the cells are clustered perfectly.

Although we varied the hyperparameters with a fold change at the scale of dozens or even as large as 100, all of the ARIs are equal to one except one setting whose corresponding ARI is 0.997. Therefore, BUSseq is robust to the specification of hyperparameters.

High zero rates

Next, we constructed a simulation dataset with high zero rates. More specifically, the zero rates of the four batches are 53.55%, 61.13%, 60.95% and 80.99%, respectively. Even though the last batch has more than 80% zero counts, BUSseq still correctly identifies the presence of five cell types and accurately estimates all the parameters (**Supplementary Fig. 5**).

Violation of model assumptions

We further applied BUSseq to a dataset where the model assumption is violated. In BUSseq, we assume that each gene's overdispersion parameter ϕ_{bg} is the same for all of the cells measured on a given batch; in the new simulation setting, we let the overdispersion parameters vary across cell types. Specifically, if a gene in a cell type has a higher expression level than the mean expression level of this gene across all cell types, then we regard this gene as an HEG. In the simulation dataset in the main text, the $K = 5$ cell types have 150, 100, 110, 100 and 155 HEGs, respectively.

In the new simulation setting, we let the overdispersion parameters vary across cell types. Under our parameterization of the negative binomial distribution, $f_{\mathrm{NB}}(x; \mu, \phi) = C_x^{\phi+x-1}(\frac{\mu}{\mu+\phi})^x(\frac{\phi}{\mu+\phi})^\phi$, the variance is $\mu + \frac{\mu^2}{\phi}$. Thus, a gene with higher ϕ_{bg} has lower overdispersion. If a gene g is highly expressed in a given cell type, we set its overdispersion parameter ϕ_{bg}^{High} in that cell type to be five times its overdispersion parameter ϕ_{bg}^{Low} in cell types where gene g is not highly expressed.

Once again, BUSseq is able to estimate the cell type effects, the batch effects and the cell-specific size factors and impute the dropout events very well when highly expressed genes have much lower true cell-type-specific overdispersion parameters (**Supplementary Fig. 6**).

Gene filtering

In real data analyses, researchers usually conduct gene filtering and focus on those HVGs [15]. Therefore, to mimic the real practice, we simulated a dataset with $G = 20,000$ genes and a total number of $N = 1,000$ cells of $K = 5$ cell types, measured by $B = 4$ batches. Among all $20,000$ genes, there are 1,326 intrinsic genes, which are differentially expressed between at least two different cell types. To select the highly variable genes, we followed the same gene

filtering step in the real data analyses to select the top 12,000 HVGs within each batch using the *trendVar* and *decomposeVar* function in the R package *scran* [16]. After gene filtering, we obtained 3,461 common HVGs across four batches (Supplementary Notes). Of the 1,326 assumed intrinsic genes, $1,223$ are retained as HVGs for the downstream analysis.

BUSseq correctly identifies the presence of five cell types, estimates all the parameters precisely, and calls $1,236$ intrinsic genes at the Bayesian false discovery rate 5% (**Supplementary Fig. 7**). As a result, all the 1,223 highly variable intrinsic genes are detected, and the actual false discovery rate is only 1.05%. Thus, gene filtering does not affect the performance of BUSseq.

Supplementary Note 7: Comparison measures and figures

ARI

The adjusted Rand index (ARI) measures the consistency between two clustering results and is between zero and one, a higher value indicating better consistency. It is defined as:

$$ARI = \frac{\sum_{i=1}^{I}\sum_{j=1}^{J}\binom{n_{ij}}{2} - [\sum_{i=1}^{I}\binom{a_i}{2}\cdot\sum_{j=1}^{J}\binom{b_j}{2}]\big/\binom{n}{2}}{(1/2)[\sum_{i=1}^{I}\binom{a_i}{2}+\sum_{j=1}^{J}\binom{b_j}{2}] - [\sum_{i=1}^{I}\binom{a_i}{2}\cdot\sum_{j=1}^{J}\binom{b_j}{2}]\big/\binom{n}{2}},$$

where n_{ij}, a_i, b_j are values from the contingency table of two clusterings. ARIs are calculated by R package *mclust* [17].

Silhouette coefficient

To evaluate the separation of different cell types after correction, we calculate the silhouette coefficient of each cell using the R package *cluster* [18]. We regard each cell type, either the truth known in the simulation study or the labeling according to FACS, as a cluster. Let $a(i)$ be the average distance of cell i to all the other cells assigned to the same cluster as cell i, and let $b(i)$ be the average distance of cell i to all cells in the neighboring cluster, i.e., the cluster with the lowest average distance to cell i's cluster. The silhouette coefficient for cell i is defined as:

$$s(i) = \frac{b(i) - a(i)}{min(a(i), b(i))}.$$

The silhouette coefficient $s(i)$ ranges from -1 to 1. The larger the values of $s(i)$, the closer cell i is to cells in the same cluster than cells in other clusters. We calculate the silhouette coefficient according to the t-SNE coordinates obtained from the corrected count data matrix (BUSseq and MNN) or from low-dimensional representations (LIGER, Scanorama, scVI, Seurat and ZINBWaVE).

Furthermore, we also compute the silhouette coefficients according to the first 10 principal components (PC) for all of the methods shown in **Supplementary Fig. 10b,c**. We set the

number of PCs as 10, as the lowest dimension of the corrected data output by all of the methods is 10, consistent with ZINB-WaVE. Once again, BUSseq has outstanding clustering performance in both the hematopoietics study and the pancreas study.

t-SNE plot

Following Haghverdi et al. [19], we apply the *Rtsne* function of the R package *Rtsne* with the perplexity set to 30. We directly input the Euclidean distances of cells to *Rtsne* and set the option *is_distance* as TRUE instead of running PCA a prior.

Mean-variance trend

We further investigated the mean-variance trend of the fitted model. Zappia et al. [20] proposed to use Splatter, an R Bioconductor package, to simulate a scRNA-seq dataset and compare its properties with those of real data.

As the first batch of each study is taken as the reference batch without batch effects, without loss of generality, here we show the mean-variance trends for the second batch $b = 2$ of both real datasets as illustrations. In the hematopoietic study, BUSseq identifies 6 cell types, with 234, 244, 216, 331, 257 and 638 cells, respectively. To generate the simulated count data for each cell type, we first calculated the estimated mean expression level $\widehat{m}_{2ig} = \widehat{\alpha}_g + \widehat{\beta}_{g,\widehat{w}_{2i}} + \widehat{\nu}_{2g} + \widehat{\delta}_{2i}$ for gene g in cell i. We then sampled the underlying expression level $\widehat{X}_{big}$s from a negative binomial distribution with mean $\widehat{m}_{2ig}$ and overdispersion $\widehat{\phi}_{2g}$. Next, we generated the dropout indicators $\widehat{Z}_{2i}$ from a Bernoulli distribution with probability $\frac{exp(\widehat{\gamma}_{20}+\widehat{\gamma}_{21}\widehat{x}_{2ig})}{1+exp(\widehat{\gamma}_{20}+\widehat{\gamma}_{21}\widehat{x}_{2ig})}$. If $\widehat{Z}_{2g} = 1$, then $\widehat{Y}_{2ig} = 0$; otherwise, $\widehat{Y}_{2ig} = \widehat{X}_{2ig}$. Following Zappia et al. [20], we plot the mean-variance trend on the log-scale. Therefore, we normalized the observed data Y_{2ig} and simulated data $\hat{Y}_{2ig}$ by counts per million reads, added one to the normalized data, and then took the logarithm to obtain the log-scale normalized expression levels Y^*_{2ig} and $\hat{Y}^*_{2ig}$. Finally, we calculated the mean μ_{gk} and variance σ_{gk} of the log-scale normalized expression levels within each cell type k (**Supplementary Fig. 9**), as well as the overall mean μ_g and variance σ_g, combining all cell

types (**Fig. 5a**) as follows:

$$\mu_{gk} = \frac{1}{m_k}\sum_{i=1}^{n_2} I(w_{2i} = k)y^*_{2ig};$$

$$\sigma_{gk} = \sqrt{\frac{1}{m_k - 1}\sum_{i=1}^{n_2} I(w_{2i} = k)(y^*_{2ig} - \mu_{gk})^2};$$

$$\mu_g = \frac{1}{n_2}\sum_{i=1}^{n_2} y^*_{2ig};$$

$$\sigma_g = \sqrt{\frac{1}{n_2 - 1}\sum_{i=1}^{n_2}\sum_{k=1}^{K} I(w_{2i} = k)(y^*_{2ig} - \mu_{gk})^2},$$

where $m_k = \sum_{i=1}^{n_2} I(w_{2i} = k)$ represents the number of cells of cell type k, $n_2 = \sum_{k=1}^{K} m_k$ denotes the total number of cells in the second batch and y^*_{2ig} can be the log-scale normalized expression levels of observed data Y^*_{2ig} or simulated data $\hat{Y}^*_{2ig}$ to calculate its corresponding mean and variance. **Fig. 5b** and **Supplementary Fig. 12** are drawn in the same manner.

Although the real data are slightly more overdispersed than the synthetic data, the mean-variance trends of the synthetic data generated according to the estimated values of BUSseq fit the mean-variance trends of the real data quite well.

Supplementary Note 8: Benchmarked batch-effects-correction methods

To ensure a fair comparison, we followed the implementation steps, especially preprocessing and clustering, of each method used for benchmarking according to their original publications [19, 21–25]. First, we respected the original clustering algorithm and analysis protocol of each benchmarked method. In the following, we briefly describe the clustering approach used by each method and summarize the approaches in **Supplementary Tab. 5**.

Linked Inference of Genomic Experimental Relationships (LIGER) [21] first normalizes the raw read count of each cell by the cell's total read counts. Then, LIGER identifies shared and batch-specific factors through integrative non-negative matrix factorization (iNMF). Next, it constructs a k-nearest-neighbor graph separately for each batch using the factor loadings of each cell. LIGER further connects two cells i and j from different batches if cell i and cell j's neighbors in their corresponding batches have similar cluster membership distributions, thus leading to a shared factor neighborhood (SFN) graph. Finally, LIGER performs Louvain community detection on the SFN graph to identify cell clusters with the *quantileAlignSNF*

function in the R package *liger* (see `https://github.com/MacoskoLab/liger`).

The mutual nearest neighbors (MNN) method [19] takes the first batch as the reference batch and normalizes the other batches to adjust for difference in sequencing depths. For clustering, MNN constructs a shared nearest neighbor (SNN) graph on the corrected count data and applies the "Walktrap" algorithm to the SNN graph (see `https://github.com/MarioniLab/MNN2017`).

Scanorama [22] conducts L_2-normalization in the preprocessing steps. For clustering, Scanorama applies k-means clustering for the corrected gene expression space to obtain 40 clusters and then assigns cell types to each cluster using knowledge of the previously provided cell-type labels. However, we do not use the cell-type label information in the clustering of other benchmarked methods, so we only assume that we know the true number of cell types K and performed the robust k-means clustering with the *pam* function in the R package *cluster* (see `https://github.com/brianhie/scanorama`).

scVI [23] directly works on the raw read count data and applies k-means clustering to their low-dimensional embedding of cells (see `https://github.com/YosefLab/scVI`).

Seurat [24] log-transforms and scales the observed read count data in the proprocessing. For clustering, Seurat first applies PCA to the dataset and then constructs an SNN graph using the first 30 principal components with the *FindNeighbors* function in the R package *Seurat*. Finally, Seurat determines the clusters by applying the *FindClusters* function in the R package *Seurat* to the SNN graph (see *Assignment of cell type labels for pancreatic islet cells* paragraph in the START★METHOD of [24]).

ZINB-WaVE [25] is directly implemented on the raw read count data with the number of factors as 10 see page 15 of Risso et al. [25] (*Clstering methods* paragraph in Methods) and finds subpopulations of cells using Seurat's clustering algorithm, following the vignettes in the R package *zinbwave* (see its vignette `https://bioconductor.org/packages/release/bioc/vignettes/zinbwave/inst/doc/intro.html#the-zinb-wave-model`).

We applied the robust k-means clustering with the *pam* function in the R package *cluster* [26] to the corrected count data, if not available then the low-dimensional embeddings, offered by each method. The FACS method labels seven cell types in both the mouse hematopoietic study and the pancreas study. Thus, we set the numbers of clusters to 7 for two real datasts when applying the k-means clustering.

For BUSseq, we ran the MCMC algorithm for 4,000, 8,000 and 8,000 iterations for the simulated data, the hematopoietic study and the pancreas study, respectively. In each case, we treated the first half of all the iterations as the burn-in period and used the posterior samples collected from the second half for statistical inference. Please see `https://github.com/songfd2018/BUSseq-1.1_implementation` for the specification of hyperparam-

eters used in this manuscript.

Besides their own clustering methods, we also applied robust k-means clustering using the *pam* function in the R package *cluster* to the corrected count data; if not available, we used the low-dimensional embeddings offered by each method. The mouse hematopoietic study involves 7 cell types labeled by FACS, including long-term hematopoietic stem cells, multi-potent progenitors, common myeloid progenitors, megakaryocyte-erythrocyte progenitor, granulocyte-monocyte progenitors, lymphoid-primed multipotent progenitors and other cells. In the human pancreas study, there are 7 cell types in the FACS labeling, including alpha cells, beta cells, delta cells, gamma cells, acinar cells, ductal cells and other cells. Thus, we set the numbers of clusters to 7 for both real datasets when applying k-means clustering. The resultant ARIs are presented in **Supplementary Tab. 5**. We can see that BUSseq still ranks the second for the hematopietic dataset and the first for the pancreas dataset.

CellAssign [27] requires prior knowledge of a set of marker genes for each cell type. We constructed marker genes for three LUAD cell lines— HCC827, H2228, H1975—using bulk RNA-seq data generated by Holik et al. [28] with the $FindMarkers$ function of the *scran* R package (see its vignette). A total of 96 marker genes are identified. We matched the gene set of each protocol to the 96 maker genes and finally obtained 85 common marker genes across the three batches. Furthermore, CellAssign works on the raw count data without normalization and requires the cell-specific size factors to be calculated over all genes. Thus, we extracted data for all common genes shared among the three batches and calculated the size factors over all common genes instead of only the common marker genes using the $computeSumFactors$ function of *scran* R package. Because there are three batches, we constructed a covariate matrix to indicate the corresponding batch of each cell. Then, we applied CellAssign with the batch information and set the other parameters the same as those in the example of the vignette (see its vignette). To evaluate the performance of clustering, CellAssign [27] calculates the accuracy and F1-score of the estimated cell types compared with the true cell type labels. We also considered the ARI between the estimated labels and true labels as a criterion.

Supplementary Note 9: BUSseq-nzf model

Here, we consider a simplified BUSseq model with no zero-inflation. The proposed model then becomes a negative binomial mixture model, abbreviated as "nzf", with the following structure:

$$Pr(W_{bi} = k) = \pi_{bk}, \sum_{k=1}^{K} \pi_{bk} = 1;$$
$$Y_{big}|W_{bi} = k \sim \text{NB}(\mu_{big}, \phi_{bg}), log(\mu_{big}) = \alpha_g + \beta_{gk} + \nu_{bg} + \delta_{bi}.$$

We compared BUSseq and BUSseq-nzf regarding both model fitting using posterior predictive check [29] for zero rates and the clustering accuracy with ARIs. Comparing the the seventh and

ninth columns of **Tab. 1** in the main text, we can see that BUSseq fits the zero rates much better than BUSseq-nzf.

For the ARIs between the estimated cell type labels $\hat{w}_{bi}$ and FACS labels, the value drops from 0.582 by BUSseq to 0.482 by BUSseq-nzf for the hematopoietic study and 0.608 by BUSseq to 0.437 by BUSseq-nzf for the pancreas study. As the modeling of dropout events does not reduce the clustering accuracy and improves the model fit dramatically, our recommendation is to use the full version of BUSseq.

Supplementary Note 10: Slingshot

Following the vignette of *slingshot* R package [30], we draw the 3-dimensional slingshot based on the clustering results by BUSseq in the hematopoietic studies (**Supplementary Fig. 11**). More specifically, the first three principal components of the corrected count data by BUSseq is used as the coordinates of each cell in the 3-dimensional figure. Then, cells are colored by their corresponding cell types inferred by BUSseq.

Supplementary Note 11: Pathway analysis

To identify the biological functions of the intrinsic genes, we conducted gene set enrichment analysis for the intrinsic genes on KEGG pathways using DAVID [1]. We controlled the Expression Analysis Systematic Explorer Score, a modified version of Fisher exact p-value, at the level of 0.05 to identify enriched pathways.

Supplementary Note 12: GPU parallelization

In the GPU version, for every iteration, parallelization is applied when:

- Proposing new values for a set of parameters or latent variables that are conditionally independent.
- Filling an array which carries the elements in the sum part of the log likelihoods/ acceptance rates.
- Summation of the individual elements in the calculation for the log likelihoods/ acceptance rates. Here, we adopt the reduction technique for GPU computing. Specifically, suppose that we want to sum over 1024 elements, in the first cycle, every two elements of the 1024 elements are summed to a total of 512 values in parallel, then the 512 numbers are further added up and reduced to 256 values in parallel in the second cycle and so on so forth. Finally, after 10 cycles, we obtain the summation over all the 1024 elements.
- Determine whether the newly proposed values are accepted and update them accordingly.

- For clearer illustration, we will explain the workflow for every parameter or latent variable involved in our algorithm.

For α_g:

- Firstly, an array with the length of G (the number of genes) is updated in parallel to store G α_g^*s, the proposed α_gs based on $\alpha_g^{[t-1]}$s from the last iteration.
- Then, for $g = 1, \cdots, G$,
  - An array of the length $\sum_{b=1}^{B} n_b = N$ (the number of cells) is filled in parallel with the N elements of the summation part in the log acceptance rate. In α_g, the log acceptance rate is

$$\sum_{b=1}^{B}\sum_{i=1}^{n_b}\Bigg\{(\alpha_g^* - \alpha_g^{[t-1]})x_{big}^{[t]} + (\phi_{bg}^{[t-1]} + x_{big}^{[t]})log\Big[\frac{\phi_{bg}^{[t-1]} + exp(\alpha_g^{[t-1]} + \beta_{gw_{bi}^{[t-1]}}^{[t-1]} + \nu_{bg}^{[t-1]} + \delta_{bi}^{[t-1]})}{\phi_{bg}^{[t-1]} + exp(\alpha_g^* + \beta_{gw_{bi}^{[t-1]}}^{[t-1]} + \nu_{bg}^{[t-1]} + \delta_{bi}^{[t-1]})}\Big]\Bigg\} - \frac{(\alpha_g^* - m_g^a)^2 - (\alpha_g^{[t-1]} - m_g^a)^2}{2\sigma_a^2},$$

  and the sum part is

$$\sum_{b=1}^{B}\sum_{i=1}^{n_b}\Bigg\{(\alpha_g^* - \alpha_g^{[t-1]})x_{big}^{[t]} + (\phi_{bg}^{[t-1]} + x_{big}^{[t]})log\Big[\frac{\phi_{bg}^{[t-1]} + exp(\alpha_g^{[t-1]} + \beta_{gw_{bi}^{[t-1]}}^{[t-1]} + \nu_{bg}^{[t-1]} + \delta_{bi}^{[t-1]})}{\phi_{bg}^{[t-1]} + exp(\alpha_g^* + \beta_{gw_{bi}^{[t-1]}}^{[t-1]} + \nu_{bg}^{[t-1]} + \delta_{bi}^{[t-1]})}\Big]\Bigg\}.$$

  - The N elements are summed with a parallelized reduction to become a single value.
- The G sums are then, in parallel, added with the constant term $-\frac{(\alpha_g^* - m_g^a)^2 - (\alpha_g^{[t-1]} - m_g^a)^2}{2\sigma_a^2}$ in the log acceptance rate, compared with G Uniform[0,1) random numbers to determine if the proposed α_g^*s are accepted, and the latest iteration of $\alpha_g^{[t]}$s are then updated accordingly.

For Z_{ig} and X_{ig}:

- For $i = 1, \cdots, N, g = 1, \cdots, G$,
  - If Y_{ig} is not zero, we assume there is no dropout and the observed signal is the real signal.
  - Otherwise, Z and X are updated simultaneously (i.e. in the same function) and in parallel across $N \times G$ based on $Z^{[t-1]}$s and $X^{[t-1]}$s from the last iteration.

For γ_{b0} and γ_{b1}:

- An array with the length of $2 \times B$ (γ_{b0} and γ_{b1} combined) is filled in parallel with γ_{b0}^* and γ_{b1}^* across B based on $\gamma_{b0}^{[t-1]}$s and $\gamma_{b1}^{[t-1]}$s from the last iteration.

- Then, for $b = 1, \cdots, B$,
  - For γ_{b0}, an array of length $n_b \times G$ (n_b is the number of samples in batch b) is filled in parallel with the $n_b \times G$ elements of the summation part in the log acceptance rate of γ_{b0}, i.e.

$$\sum_{i=1}^{n_b}\sum_{g=1}^{G}\Big\{(\gamma_{b0}^* - \gamma_{b0}^{[t-1]})z_{big}^{[t]} + log\Big[\frac{1 + exp(\gamma_{b1}^{[t-1]}x_{big}^{[t]} + \gamma_{b0}^{[t-1]})}{1 + exp(\gamma_{b1}^{[t-1]}x_{big}^{[t]} + \gamma_{b0}^*)}\Big]\Big\}.$$

  - For γ_{b1}, an array of length $n_b \times G$ (n_b is the number of samples in batch b) is filled in parallel with the $n_b \times G$ elements of the summation part in the log acceptance rate of γ_{b1}, i.e.

$$\sum_{i=1}^{n_b}\sum_{g=1}^{G}\Big\{(\gamma_{b1}^* - \gamma_{b1}^{[t-1]})x_{big}^{[t]}z_{big}^{[t]} + log\Big[\frac{1 + exp(\gamma_{b1}^{[t-1]}x_{big}^{[t]} + \gamma_{b0}^{[t]})}{1 + exp(\gamma_{b1}^* x_{big}^{[t]} + \gamma_{b0}^{[t]})}\Big]\Big\}.$$

  - The two arrays are summed through parallelized reduction into 2 single values.

- The B sums of γ_{b0} are then added in parallel with the constant term $-\frac{(\gamma_{b0}^*)^2 - (\gamma_{b0}^{[t-1]})^2}{2\sigma_{\gamma 0}^2}$ in the log acceptance rate, compared with B Uniform[0,1) random numbers to determine if the proposed γ_{b0}^*s are accepted, and the latest iteration of $\gamma_{b0}^{[t]}$s are then updated accordingly.

- The B sums of γ_{b1} are then added in parallel with the constant term

$$a_\gamma log(\frac{-\gamma_{b1}^*}{-\gamma_{b1}^{[t-1]}}) + b_\gamma(\gamma_{b1}^* - \gamma_{b1}^{[t-1]}) - log\Big[\frac{\Gamma(-10\gamma_{b1}^*)}{\Gamma(-10\gamma_{b1}^{[t-1]})}\Big]$$

$$-10\gamma_{b1}^* log(-\gamma_{b1}^{[t-1]}) + 10\gamma_{b1}^{[t-1]} log(-\gamma_{b1}^*) - 10(\gamma_{b1}^* - \gamma_{b1}^{[t-1]})(log(10) + 1)$$

  in the log acceptance rate, compared with B Uniform[0,1) random numbers to determine if the proposed γ_{b1}^*s are accepted, and the latest iteration of $\gamma_{b1}^{[t]}$s are then updated accordingly.

For L_{gk}:

- For $g = 1, \cdots, G, k = 2, \cdots, K$, acceptance rates and random acceptances for every L_{gk} are computed simultaneously and in parallel.

For p and $(\tau_{\beta 0})^2$:

- For $g = 1, \cdots, G, k = 2, \cdots, K$,
  - The array of $L_{gk}^{[t]}$ is summed through parallelized reduction into one single value.
  - An array of length $G \times (K-1)$ is filled in parallel with $I(L_{gk}^{[t]} = 0) \cdot (\beta_{gk}^{[t-1]})^2$, where 1 is the indicator function.
  - This array is then summed through parallelized reduction into one single value.
- A single Beta random variable, parameterized with terms consisting of the sum of $L_{gk}^{[t]}$, is generated as $p^{[t]}$(p of the latest iteration).
- A single Inv-Gamma random variable, parameterized with terms consisting of the two sums above, is generated as $(\tau_{\beta 0}^{[t]})^2$($(\tau_{\beta 0})^2$ of the latest iteration).

For β_{gk}:

- Firstly, an array with the length of $G \times (K-1)$ is updated in parallel to store $G \times (K-1)$ β_{gk}^*s, the proposed β_{gk}s based on $\beta_{gk}^{[t-1]}$s from the last iteration.
- For $g = 1, \cdots, G, k = 2, \cdots, K$,
  - An array of the length $\sum_{b=1}^{B} n_b = N$ (no. of samples) is filled in parallel with the N elements of the summation part in the log acceptance rate, i.e.

$$\sum_{b=1}^{B}\sum_{i=1}^{n_b} I(w_{bi}^{[t-1]} = k)\Big\{(\beta_{gk}^* - \beta_{gk}^{[t-1]})x_{big}^{[t]} + (\phi_{bg}^{[t-1]} + x_{big}^{[t]})log\Big(\frac{\phi_{bg}^{[t-1]} + exp(\alpha_g^{[t]} + \beta_{gk}^{[t-1]} + \nu_{bg}^{[t-1]} + \delta_{bi}^{[t-1]})}{\phi_{bg}^{[t-1]} + exp(\alpha_g^{[t]} + \beta_{gk}^* + \nu_{bg}^{[t-1]} + \delta_{bi}^{[t-1]})}\Big)\Big\}.$$

  - The array is then summed by with a parallelized reduction to become a single value.
- The $G \times (K-1)$ sums are then added in parallel with the constant term $-\frac{(\beta_{gk}^*)^2 - (\beta_{gk}^{[t-1]})^2}{2(\tau_{\beta L_{gk}^{[t]}}^{[t]})^2}$ in the log acceptance rate, compared with $G \times (K-1)$ Uniform[0,1) random numbers to determine if the proposed β_{gk}^*s are accepted, and $\beta_{gk}^{[t]}$ (β_{gk} of the latest iteration) are then updated accordingly.

For ν_{bg}:

- Firstly, an array with the length of $G \times (B-1)$ is updated in parallel to store $G \times (B-1)$ ν_{bg}^*s, the proposed ν_{bg}s based on $\nu_{bg}^{[t-1]}$s from the last iteration.

- For $b = 2, \cdots, B, g = 1, \cdots, G$,
  - An array with the length of n_b is filled in parallel with the n_b elements of the summation part in the log acceptance rate, i.e.

$$\sum_{i=1}^{n_b} \left\{ (\nu_{bg}^{*} - \nu_{bg}^{[t-1]}) x_{big}^{[t]} + (\phi_{bg}^{[t-1]} + x_{big}^{[t]}) log \left[\frac{\phi_{bg}^{[t-1]} + exp(\alpha_g^{[t]} + \beta_{gk}^{[t]} + \nu_{bg}^{[t-1]} + \delta_{bi}^{[t-1]})}{\phi_{bg}^{[t-1]} + exp(\alpha_g^{[t]} + \beta_{gk}^{[t]} + \nu_{bg}^{*} + \delta_{bi}^{[t-1]})} \right] \right\}.$$

  - The array is then summed with a parallelized reduction to become a single value.
- The $(B-1) \times G$ sums are then added in parallel with the constant term $-\frac{(\nu_{bg}^{*} - m_{bg}^{c})^2 - (\nu_{bg}^{[t-1]} - m_{bg}^{c})^2}{2\sigma_c^2}$ in the log acceptance rate, compared with $(B-1) \times G$ Uniform[0,1) random numbers to determine if the proposed ν_{bg}^{*}s are accepted, and $\nu_{bg}^{[t]}$ (ν_{bg} of the latest iteration) are then updated accordingly.

For δ_{bi}:

- Firstly, an array with the length of N is updated in parallel to store $\sum_{b=1}^{B} n_b = N$ δ_{bi}^{*}s, the proposed δ_{bi}s based on $\delta_{bi}^{[t-1]}$s from the last iteration.
- For $b = 1, \cdots, B, i = 2, \cdots, n_b$,
  - An array with the length of G is filled in parallel with the G elements of the summation part in the log acceptance rate, i.e.

$$\sum_{g=1}^{G} \left\{ (\delta_{bi}^{*} - \delta_{bi}^{[t-1]}) x_{big}^{[t]} + (\phi_{bg}^{[t-1]} + x_{big}^{[t]}) log \left[\frac{\phi_{bg}^{[t-1]} + exp(\alpha_g^{[t]} + \beta_{gk}^{[t]} + \nu_{bg}^{[t]} + \delta_{bi}^{[t-1]})}{\phi_{bg}^{[t-1]} + exp(\alpha_g^{[t]} + \beta_{gk}^{[t]} + \nu_{bg}^{[t]} + \delta_{bi}^{*})} \right] \right\}.$$

  - The array is then summed with a parallelized reduction to become a single value.
- The N sums are then added in parallel with the constant term $-\frac{(\delta_{bi}^{*} - m_{bi}^{d})^2 - (\delta_{bi}^{[t-1]} - m_{bi}^{d})^2}{2\sigma_d^2}$ in the log acceptance rate, compared with G Uniform[0,1) random numbers to determine if the proposed δ_{bi}^{*}s are accepted, and $\delta_{bi}^{[t]}$ (δ_{bi} of the latest iteration) are then updated accordingly.

For ϕ_{bg}:

- Firstly, an array with the length of $B \times G$ is updated in parallel to store $B \times G$ ϕ_{bg}^{*}s, the proposed ϕ_{bg}s based on $\phi_{bg}^{[t-1]}$s from the last iteration.
- For $b = 1, \cdots, B, g = 1, \cdots, G$,
  - Let $\eta_{big}^{[t]} = exp(\alpha_g^{[t]} + \beta_{gw_{bi}^{[t-1]}}^{[t]} + \nu_{bg}^{[t]} + \delta_{bi}^{[t]})$ denotes the mean gene expression level for gene g in cell i of batch b.

- An array with the length of n_b is filled in parallel with the n_b elements of the summation part in the log acceptance rate, i.e.

$$\sum_{i=1}^{n_b}\Bigg\{log\Big[\frac{\Gamma(\phi_{bg}^{*}+x_{big}^{[t]})(\phi_{bg}^{*})^{\phi_{bg}^{*}}}{\Gamma(\phi_{bg}^{*})(\phi_{bg}^{*}+\eta_{big}^{[t]})^{\phi_{bg}^{*}+x_{big}^{[t]}}}\Big]+log\Big[\frac{\Gamma(\phi_{bg}^{[t-1]})(\phi_{bg}^{[t-1]}+\eta_{big}^{[t]})^{\phi_{bg}^{[t-1]}+x_{big}^{[t]}}}{\Gamma(\phi_{bg}^{[t-1]}+x_{big}^{[t]})(\phi_{bg}^{[t-1]})^{\phi_{bg}^{[t-1]}}}\Big]\Bigg\},$$

- The array is then summed with a parallelized reduction to become a single value.

- The $B \times G$ sums are then added in parallel with the constant term $(a_\phi - 1)log(\frac{\phi_{bg}^{*}}{\phi_{bg}^{[t-1]}}) + (1-b_\phi)(\phi_{bg}^{*}-\phi_{bg}^{[t-1]}) + log\Big[\frac{(\phi_{bg}^{[t-1]})^{\phi_{bg}^{*}-1}\Gamma(\phi_{bg}^{[t-1]})}{(\phi_{bg}^{*})^{\phi_{bg}^{[t-1]}-1}\Gamma(\phi_{bg}^{*})}\Big]$ in the log acceptance rate, compared with $B \times G$ Uniform[0,1) random numbers to determine if the proposed ϕ_{bg}^{*}s are accepted, and $\phi_{bg}^{[t]}$ (ϕ_{bh} of the latest iteration) are then updated accordingly.

For w_{bi}:

- Firstly, an array with the length of N is updated in parallel to store $\sum_{b=1}^{B} n_b = N$ w_{bi}^{*}s, the proposed w_{bi}s based on $w_{bi}^{[t-1]}$s from the last iteration.
- For $b = 1, \cdots, B, i = 1, \cdots, n_b$,
  - An array with the length of G is filled in parallel with the G elements of the summation part in the log acceptance rate, i.e.

    $$\sum_{g=1}^{G}\Bigg\{(\beta_{gk^*}^{[t]}-\beta_{gk}^{[t]})x_{big}^{[t]}+(x_{big}^{[t]}+\phi_{bg}^{[t]})log\Big[\frac{exp(\alpha_g^{[t]}+\beta_{gk}^{[t]}+\nu_{bg}^{[t]}+\delta_{bi}^{[t]})+\phi_{bg}^{[t]}}{exp(\alpha_g^{[t]}+\beta_{gk^*}^{[t]}+\nu_{bg}^{[t]}+\delta_{bi}^{[t]})+\phi_{bg}^{[t]}}\Big]\Bigg\},$$

    where $w_{bi}^{*} = k^{*}, w_{bi}^{[t-1]} = k$.
  - The array is then summed with a parallelized reduction to become a single value.
- The N sums are then in parallel added with the constant term $\log(\frac{\pi_{bk^*}^{[t-1]}}{\pi_{bk}^{[t-1]}})$ in the log acceptance rate, compared with N Uniform[0,1) random numbers to determine if the proposed w_{bi}^{*}s are accepted, and $w_{bi}^{[t]}$ (w_{bi} of the latest iteration) are then updated accordingly.

For $\boldsymbol{\pi}_b$:

- For $b = 1, \cdots, B, k = 1, \cdots, K$,
  - An array with the length of n_b is filled in parallel with the value of the function $I(w_{bi} = k)$, where 1 is the indicator function.
  - The array is then summed with a parallelized reduction to become a single value.

- The $N \times K$ sums are then added in parallel with a constant ξ, the parameter of the prior distribution.
- For $b = 1, \cdots, B$, $\boldsymbol{\pi}_b^{[t]}$, $\boldsymbol{\pi}_b$ of the latest iteration, is updated in parallel by sampling from the Dirichlet distribution with the sums above as parameters.

For more details, please refer to the MCMC Algorithm for BUSseq in the supplementary notes.

Supplementary Note 13: Scalability

We ran BUSseq on simulation datasets with different numbers of genes or cells to evaluate the scalability of BUSseq in the running time and RAM usage. Theoretically, the time complexity of the MCMC algorithm is $O(NGK)$ in terms of the number of genes G and the total number of cells $N = \sum_{b=1}^{B} n_b$. On the other hand, the dominating memory consumption is the storage of the posterior samples in the MCMC algorithm. Fortunately, if given a machine with small random-access memory (RAM), we can write the posterior samples to the hard disk every n_s iterations and then load the recorded posterior samples one iteration a time when conducting posterior inference. Consequently, by storing the posterior samples from the RAM to the hard disk every a few iterations, we can control the memory consumption. For instance, we can run a total of 4,000 iterations and store the posterior samples to the hard disk after every 1,000 iterations. Theoretically, when n_s is large, the RAM consumption is proportional to n_s and to the total number of parameters $|\boldsymbol{\Theta}|$. $|\boldsymbol{\Theta}| = O(N + 2(B + K)G)$, where B denotes the number of batches and K represents the number of cell types. On the other hand, when n_s is small, the RAM consumption is dominated by the two count data matrices $\boldsymbol{X}$ and $\boldsymbol{Y}$ and the dropout indicators $\boldsymbol{Z}$, whose memory consumption is $O(N \cdot G)$. Therefore, the RAM consumption should be $O((N + 2(B + K)G)n_s + 3NG)$ when both K and B are far smaller than N or G, the typical case for scRNA-seq data.

To verify the theoretical analysis, we recorded how the RAM consumption and the running time varied with the number of genes G, the number of cells N and the number of iterations n_s per hard-disk writing for the CPU parallel version of the code. **Supplementary Tab. 11** shows that the RAM consumption increases linearly with respect to the number of cells N, the number of genes G and the number of iterations per storage n_s. Meanwhile, the running time is linear to the number of cells N and the number of genes G. Although we can expect that writing to the hard-disk can take longer than writing to RAM, it turned out that the running time is almost constant to the frequency of writing to hard disk when running BUSseq on a cluster node with two 8-core Dual Intel Xeon E5-2650 v2 2.60GHz processors. For example, if we apply BUSseq on a scRNA-seq data with 100,000 cells and 3,000 genes and store the posterior sampling to the hard disk per 10 iterations, the running time and the RAM consumption can be estimated by multiplying 1000 on those of the case with $N = 1000, G = 3000$ and $n_s = 10$. According to **Supplementary Tab. 11**, it will consume 6.5 GB RAM to run BUSseq on the dataset with 100,000 cells. Therefore, in principle, as long as we write out the posterior samples to the hard disk frequently (e.g. every 10 iterations), we can run BUSseq on a huge scRNA-seq dataset with

100,000 cells and 3,000 genes on a desktop with 8G RAM. However, taking time consumption into consideration, we recommend running BUSseq on a cluster node instead.

Supplementary Note 14: Convergence diagnostic

EPSR factors

We start $\frac{m}{2}$ chains (assign $\frac{m}{2}$) with different initial values and run each chain for $4n$ iterations, where m is even ($m \geq 4$) and n is an integer. For each chain, we keep only the second half of the iterations, ignoring the first half as burn-ins. This results in $\frac{m}{2}$ chains with $2n$ iterations each. Next, we further cut each chain into two chains of equal length, obtaining $m = \frac{m}{2} \times 2$ chains, with $n = \frac{2n}{2}$ iterations each. Let θ denote a parameter of interest; we then use $\theta_{ij}, i = 1, 2, \cdots, n, j = 1, 2, \cdots, m$ to indicate the ith sample of θ collected in chain j. With these notations, we define

$$\bar{\theta}_{\cdot j} = \frac{1}{n}\sum_{i=1}^{n}\theta_{ij},\ \bar{\theta}_{\cdot\cdot} = \frac{1}{m}\sum_{i=1}^{n}\bar{\theta}_{\cdot j},\ s_j^2 = \frac{1}{n-1}\sum_{i=1}^{n}(\theta_{ij} - \bar{\theta}_{\cdot j})^2,$$
$$B = \frac{n}{m-1}\sum_{j=1}^{m}(\bar{\theta}_{\cdot j} - \bar{\theta}_{\cdot\cdot})^2,\ W = \frac{1}{m}\sum_{j=1}^{m}s_j^2.$$

B describes the between-chain variance, and W measures the within-chain variances. When multiple chains become stationary and mix well with each other, B should be very close to W. Subsequently, the EPSR factor for assessing the convergence of θ, which is defined as $\widehat{R} = \sqrt{\frac{n-1}{n} + \frac{1}{n}\frac{B}{W}}$, should also be close to one.

We are interested in the log-scale baseline expression level $\{\alpha_g, g = 1, 2, \cdots, G\}$, the cell type effects $\{\beta_{gk}, g = 1, 2, \cdots, G, k = 2, 3, \cdots, K\}$, the location batch effects $\{\nu_{bg}, g = 1, 2, \cdots, G, b = 2, 3, \cdots, B\}$ and the overdispersion parameters $\{\phi_{bg}, g = 1, 2, \cdots, G, b = 1, 2, \cdots, B\}$. To avoid the impact of label switching of cell types, we consider the log-scale cell type specific expression level $\theta_{gk} = \alpha_g + \beta_{gk}, g = 1, 2, \cdots, G, k = 1, 2, \cdots, K$ and match the cell type indicators in different chains such that most cells in the different chains are assigned to the same cell types. If the EPSR factors of most parameters are close to one, we treat the posterior sampling as attaining stationary. Thus, we used the following rule to diagnose the convergence of the MCMC algorithm for BUSseq:

1. More than 80% of $\{\text{EPSR}(\theta_{gk})\}$ are less than 1.3
2. More than 80% of $\{\text{EPSR}(\nu_{bg})\}$ are less than 1.3
3. More than 80% of $\{\text{EPSR}(\phi_{bg})\}$ are less than 1.3

We calculated the EPSR factors in the simulation study and the two real applications. In the simulation, we initiated $\frac{m}{2} = 2$ chains. After running 4,000 iterations and taking the first 2,000

as burn-ins, 98.29% of {EPSR(θ_{gk})} are less than 1.3, 98.19% of {EPSR(ν_{bg})} are less than 1.3, and 99.09% of {EPSR(ϕ_{bg})} are less than 1.3. Therefore, the MCMC algorithm has converged after 2,000 burn-in iterations. In the mouse hematopoietic study, we also initiated two chains. After running 8,000 iterations and taking the first 4,000 as burn-ins, 89.09% of {EPSR(θ_{gk})} are less than 1.3, 88.73% of {EPSR(ν_{bg})} are less than 1.3, and 97.94% of {EPSR(ϕ_{bg})} are less than 1.3. In the pancreas study, we also initiated two chains. After running 8,000 iterations and taking the first 4,000 as burn-ins, 84.08% of {EPSR(θ_{gk})} are less than 1.3, 86.03% of {EPSR(ν_{bg})} are less than 1.3, and 97.96% of {EPSR(ϕ_{bg})} are less than 1.3.

Acceptance rate of Metropolis-Hasting updates

The parameters using Metropolis-Hasting updates include $\gamma_{b0}, \gamma_{b1}, \alpha_g, \beta_{gk}, \nu_{bg}, \delta_{bi}$ and ϕ_{bg}. Gelman et al. [31] studied the most efficient symmetric jumping kernels for simulating a normal target distribution using the Metropolis algorithm. Theoretically, they found that the optimal target acceptance rate is 0.44 when the target distribution is a one-dimensional normal distribution. The acceptance rates of most parameters of BUSseq except $\boldsymbol{\gamma}$ are close to 0.44 (**Supplementary Tab. 13**). Although the acceptance rate of $\boldsymbol{\gamma}$ is low (**Supplementary Tab. 13**), the comparison of the posterior means $\hat{\boldsymbol{\gamma}}$ and their true values $\boldsymbol{\gamma}$ in **Supplementary Tab. 14** show that the inference of $\boldsymbol{\gamma}$ is precise. Although adaptive MCMC algorithms can have better mixing properties than comparable non-adaptive algorithms [32], we find the non-adaptive algorithms work reasonably well in our case, so we opt to stick with the non-adaptive MCMC.

Label switching

Label switching is often observed for low dimensional mixture models. However, for high-dimensional models, our experience is that samples from MCMC algorithms are likely to wander around one of the symmetric modes of the posterior distribution for a very long time as it becomes difficult to jump from one symmetric mode to another. Ideally, we could have waited for the samples to explore all of the symmetric modes and then applied label switching algorithms for adjustment and inference. In practice, we found that performing posterior inference based on samples around one of the symmetric modes of the posterior distribution gave good enough results. In neither the simulation studies nor the real data analyses did we observe label switching among the samples that we collected. Moreover, we checked the posterior probability of $Pr(W_{bi} = k|\widehat{\boldsymbol{X}}, \widehat{\boldsymbol{\Theta}})$ for each cell based on the posterior mean estimates of the parameters $\widehat{\boldsymbol{\Theta}} = (\widehat{\boldsymbol{\alpha}}, \widehat{\boldsymbol{\beta}}, \widehat{\boldsymbol{\gamma}}, \widehat{\boldsymbol{\nu}}, \widehat{\boldsymbol{\phi}}, \widehat{\boldsymbol{\delta}}, \widehat{\boldsymbol{\pi}})$.

$$Pr(W_{bi} = k|\widehat{\boldsymbol{X}}, \widehat{\boldsymbol{\Theta}}) \propto \widehat{\pi}_{bk} \prod_{g=1}^{G} \frac{exp(\widehat{\beta}_{gk}\widehat{x}_{big})}{(exp(\widehat{\alpha}_g + \widehat{\beta}_{gk} + \widehat{\nu}_{bg} + \widehat{\delta}_{bi}) + \widehat{\phi}_{bg})^{\widehat{x}_{big}+\widehat{\phi}_{bg}}},$$

where $\widehat{\boldsymbol{X}}$ denotes the imputed read counts. Recall that we assign cell i from batch b to cell type w_{bi} if $w_{bi} = arg\max_k Pr(W_{bi} = k|\widehat{\boldsymbol{X}})$. In **Supplementary Fig. 16**, we draw the distribution

of $\max_k Pr(W_{bi} = k|\widehat{\boldsymbol{X}}, \widehat{\boldsymbol{\Theta}})$ for all the cells for the two real applications. We can see that the highest conditional probabilities are always very close to one for most cells in these two studies. Thus, we claim that no label switching issue exists in our inference.

Supplementary Note 15: Preprocessing details

Hematopoietic study

There were two batches of gene expression count data, one generated on the SMART-seq2 platform by [33] with accession number GSE81682 and another generated on the MARS-seq platform by [34] with accession number GSE72857. Haghverdi et al. [19] first labeled the cells using FACS and then performed size factor normalization within each batch. Next, the authors filtered out the common HVGs identified by [33] between the two datasets. These HVGs were denoted by their Ensembl ID. The genes in the GSE81682 dataset were named by Ensembl ID, but the genes in the GSE72857 dataset were named by Gene Symbol. The authors used the R package *biomaRt* to query the corresponding Gene Symbol by Ensembl ID. Finally, there were 3,470 common HVGs between the two datasets in our manuscript.

Moreover, to alleviate the impact of sequencing depths, Haghverdi et al. [19] used m_g^{MARS} and m_g^{SMART} to denote the mean expression levels of gene g across all cells in the MARS-seq and SMART-seq2 batches, respectively, and scaled the MARS-seq data to match the coverage of the SMART-seq2 batch using the median of the mean expression ratio $\rho = \text{Median}(\frac{m_g^{\text{SMART}}}{m_g^{\text{MARS}}})$ across all genes: $y_{ig}^{\text{scaled}} = \rho \cdot y_{ig}$, where y_{ig} denotes the normalized expression levels of gene g in the ith cell of the MARS-seq batch. Thus, we draw t-SNE and PCA plots of the uncorrected count data after applying size factor normalization and gene coverage scaling between the two datasets (**Supplementary Fig. 8**).

Because different correction methods apply different normalization strategies and some methods (BUSseq and ZINB-WaVE) do not require prior normalization, we created PCA and t-SNE plots using the raw count data without normalization (**Fig. 4**).

Pancreas study

Haghverdi et al. [19] first removed poor-quality cells and genes and then normalized the raw count data using the deconvolution methods proposed by Lun et al. [5]. Next, the authors identified HVGs within each batch following [15]. Two batches (GSE86473 and EMATB-5061) had the cell-type labels for all of the cells, but the cell type labels of the other two datasets profiled by the CEL-seq2 platform (GSE81076 and GSE85241) were inferred by the marker genes used in the original publications by Lawlor et al. [35] and Grün et al. [36].

To assign cell type labels for the GSE81076 and GSE85241 datasets, following Haghverdi et al. [19], we first extracted the normalized expression levels of the selected HVGs within each

dataset, respectively. Next, we obtained the low dimensional embedding of HVGs by tSNE for visualization. At the same time, we applied robust k-means clustering to the normalized expression levels of the selected HVGs using the *pam* function in the R package *cluster*. The number of clusters was set as 9. Next, we drew t-SNE plots colored by the expression levels of the marker genes. GCG is highly expressed in alpha islet cells, INS in beta islet cells, SST in delta islet cells, PPY in gamma islet cells (pancreatic polypeptide cells), $PRSS1$ in acinar cells, $KRT19$ in ductal cells and $COL1A1$ in mescenchymal cells [35, 36]. Finally, we labeled each cluster by its corresponding highly expressed marker gene.

Similar to the preprocessing pipeline in the hematopoietic study, we drew PCA and tSNE plots of the raw count data without normalization. In **Supplementary Fig. 8**, we show t-SNE and PCA plots of the normalized uncorrected count data.

LUAD study

Different levels of data sparsity

One experiment of the LUAD study assayed three lung adenocarcinoma cell lines—HCC827, H1975 and H2228—on three platforms with CELseq2, 10x Chromium and Drop-seq protocols, respectively. The accession number of this study is GSE118767. As a result, each batch consists of three cell types, and data from different batches have different levels of sparsity. We downloaded the raw count data from the GitHub repository "https://github.com/LuyiTian/sc_mixology." We selected the top 6,000 highly variable genes (HVGs) within each batch using the $trendVar$ and $decomposeVar$ functions in the R package *scran* [16] and obtained 2,267 common HVGs across three batches.

The t-SNE and PCA plots of the raw count data (**Fig. 8**) show that significant batch effects occurred across the three protocols. Thus, we applied BUSseq and varied the number of cell type K from 2 to 6. The Bayesian information criterion (BIC) obtains its minimum value at $K = 4$ (**Supplementary Fig. 14**). Although the BIC selects four cell types instead of three cell lines, two of the four identified clusters actually correspond to two subpopulations of the H1975 cell lines (**Supplementary Tab. 8**). We further visualize the log-scale mean expression levels of intrinsic genes of the four learned cell types **(Fig. 8e)**. The first two cell types have similar expression patterns, but some differentially expressed genes are observed between them. Moreover, the PCA (**Supplementary Fig. 13c**) and t-SNE (**Fig. 8a**) plots demonstrate the high level of similarity of the first two estimated cell types. Meanwhile, the PCA (**Supplementary Fig. 13d**) and tSNE (**Fig. 8b**) plots confirm that the corrected count data $\tilde{x}_{big}$ obtained by BUSseq cluster cells by cell type rather than by batch (**Fig. 8f**). We also applied the benchmarked methods to evaluate their clustering accuracy. Once again, BUSseq outperforms all of the other methods according to ARIs.

Different levels of sample complexity

Tian et al. [37] also assayed four batches of pseudo-cells profiled by CELseq2 protocol. Specifi-

cally, in each batch, single cells from the three cell lines were sorted into 384-well plates with 9 cells per well (a small proportion of wells had only 3 cells to mimic small cells) in different combinations. RNAs from all of the 9 cells in the same well were then pooled and sub-sampled such that an approximately single-cell quantity of RNA was extracted from each well. [37] used a triad (a, b, c) to denote a combination of a HCC827 cells, b H1975 cells and c H2228 cells for each well, and generated 35 groups (34 9-cell mixtures and 1 small (three-cell) mixture) of pseudo cells with distinct combinations. Pseudo cells are much more similar to each other than cells from the three pure cell lines, so we can assess the performance of BUSseq in the experiment with a complex cell population.

To assess the performance of selecting the number of cell types by BIC across different levels of sample complexity, we first applied BUSseq to three pure cell line mixtures, following [37]. Specifically, we collected cells in the three pure cell line mixtures—denoted by the triads (9,0,0), (0,9,0) and (0,0,9)—in the four batches. Again, we downloaded the raw count data from the GitHub repository "https://github.com/LuyiTian/sc_mixology." We selected the top 6,000 HVGs in each of the four batches using the R package *scran* [16] and used the common HVGs across the four batches for the downstream analysis. The t-SNE and PCA plots in **Supplementary Fig. 18a-d** show that batch effects occur, but are smaller than those induced by different protocols in the first set of experiments. We applied BUSseq to the resulting dataset and varied the number of cell types K from 2 to 6. BUSseq successfully learned the number of cell types as $K = 3$ by BIC (**Supplementary Fig. 17a**) and perfectly clusters these cells such that the adjusted random index (ARI) is one. After batch effects correction, cells from the same cell line but assayed in different batches are clustered together (**Supplementary Fig. 18e-h**).

We then incorporated two intermediate cell line mixtures (4,0,5) and (5,0,4) into the analysis in addition to the pure cell line mixtures (9,0,0), (0,9,0) and (0,0,9), to mimic an intermediate "development state" between HCC827 cells and H2228 cells. We provided PCA and t-SNE plots of the raw count data (**Supplementary Fig. 19a-d**). We applied BUSseq to this dataset with five cell line mixtures. **Supplementary Tab. 15** shows the distribution of each cell line mixture involved.

However, BIC still selected the optional number of cell types as $K = 3$ for the dataset with five cell line mixtures. The cells in the cell line mixture (5,0,4) or (4,0,5) are clustered into the two pure cell line mixtures (9,0,0) and (0,0,9). After batch effects correction, these intermediate cells are located between the two pure cell line clusters (**Supplementary Fig. 19e**), but they fail to generate a new cluster. The main reason for the failure of BIC to identify the existence of four or five clusters is the limited sample size—there are only 215 cells in total for the five cell line mixtures. To differentiate between more subtle cell type differences, statistical inference requires larger sample sizes. Nevertheless, **Supplementary Fig. 19e** demonstrate that BUSseq is able to preserve the "development trajectory."

Identifying rare cell types

We subsampled cells in the cell line mixture study to mimic unevenly distributed rare cell types. Here, we consider the three pure cell lines $(9, 0, 0)$, $(0, 9, 0)$ and $(0, 0, 9)$ that correspond to the HCC827, H1975 and H2228 cell lines, respectively, in the LUAD dataset. **Supplementary Tab. 15** shows the cell numbers of the three pure cell lines in the four batches. We reduced the number of HCC827 cells by random sampling and investigated whether we can still identify HCC827 cells when they become rare.

Specifically, we subsampled m HCC827 cells from each batch, varying m among $(10, 8, 6, 5, 4, 3, 2, 1)$. Note that $m = 18$ indicates that all HCC827 cells were involved. We then applied BUSseq to the resulting datasets with $K = 3$. For each dataset, we selected the top 6,000 HVGs within each of the four batches using the R package *scran* [16] and took the common HVGs across the four batches for the downstream analysis. We ran BUSseq for 8,000 iterations and discarded the first 4,000 iterations as burn-ins. Finally, we calculated the ARIs between the identified cell clusters and the true cell type labels. BUSseq perfectly clusters the rare cell type HCC827 unless there is only one HCC827 cell in each batch (**Supplementary Tab. 16**).

In summary, when the number of cell type is unknown, BUSseq learns the number of cell types using BIC. The BIC balances the goodness-of-fit of the model to the data and the complexity of the model. As with all statistical methods, detecting weak signals asks for a large sample size. Thus, given a small sample size, BUSseq is able to capture the clustering hierarchy but may merge close cell types with subtle differences into one group (**Supplementary Figs. 17-19**). Nevertheless, the corrected count data output by BUSseq preserve the biological variabilities (**Supplementary Fig. 19e-h**), and researchers can perform downstream analysis using the corrected count data. Besides, when the number of cell types is known a priori as assumed by Zhang et al. [27], we find that BUSseq is able to detect rare cell types under such an extreme case that one cell type has only two cells per batch in real data analysis (**Supplementary Tab. 16**). Calculating the exact sample size required to differentiate two high-dimensional vectors is an active research field [38–41], and we will further investigate the sample size calculation problem for BUSseq in our future study.

Supplementary Note 16: Posterior predictive check

In the posterior predictive check, we take MCMC samples of all of the parameters after the burn-in iterations to simulate replicated datasets $Y_j^{rep}, j = 1, 2, \cdots, J$ for G genes and $N = \sum_{b=1}^{B} n_b$ cells, where J denotes the total number of collected iterations after burn-ins. In our real data analyses, we ran 8,000 iterations with the first 4,000 iterations as burn-ins, so we generated $J = 8,000 - 4,000 = 4,000$ replicated datasets for both the hematopoietic and pancreas studies. For each generated replicate dataset, we calculated the zero rates of each batch by

$$\rho_0 = \frac{1}{G \cdot n_b} \sum_{g=1}^{G} \sum_{i=1}^{n_b} I(y_{big} = 0).$$

Finally, we averaged the zero rates over all J iterations to calculate the posterior mean $\widehat{\rho}_0$ of the zero rate of each batch and compared it with the corresponding observed zero rate.

Supplementary Note 17: Comparison of gene filtering strategies

In addition to filtering highly variable genes, we also followed Duò et al. [42] in selecting the genes with the highest mean expression levels across all cells. In preprocessing, Duò et al. [42] first excluded the low quality genes and cells. Next, the normalization factors for the count values were calculated by the deconvolution method of the R package *scater*. For gene filtering, the authors retained genes with the top 10% highest average of log-scale expression values across all cells.

In the hematopoietic study, there are two batches of gene expression count data, one generated on the SMART-seq2 platform by Nestorowa et al. [33] with accession number GSE81682 and the other generated on the MARS-seq platform by Paul et al. [34] with accession number GSE72857. After removing low-quality genes, GSE81682 includes 46,175 genes, and GSE72857 contains 27,297 genes. Notably, the genes in the GSE81682 dataset were named by Ensembl ID, but the genes in the GSE72857 dataset were named by Gene Symbol. The R package *biomaRt* was applied to query the corresponding Gene Symbol by Ensembl ID. However, if we had retained only genes with the top 10% highest average of log-scale expression values, only 74 common genes would have been filtered out by both datasets. Instead, we retained genes with the top 50% highest average of log-scale expression values such that 2,843 common genes were retained from the two datasets for downstream analysis. We denote these genes as top ranked genes in terms of mean expression levels across cells (TEG).

For comparison, in our original analysis, following Haghverdi et al. [19], we had a dataset with 3,470 highly variable genes (HVG). We compared the results of applying BUSseq to the new dataset with the 2,843 TEGs to that of our original analysis (**Supplementary Tab. 17**). We can see that the diagonal elements dominate each row. The ARI between the cell labeling of TEGs and FACS labeling is 0.482, whereas the ARI between the cell labeling of HVGs and FACS labeling is 0.582.

We also compared the intrinsic genes identified by BUSseq when applied to the two datasets with different gene filtering criteria. The 3,470 selected HVGs and the 2,843 selected TEGs share only 475 genes. Of these 475 genes, 194 genes are identified as intrinsic genes by the dataset with HVGs, and 193 of these 194 genes were also called intrinsic genes by the dataset with TEGs (the dataset with TEGs called a total of 174 intrinsic genes for the 475 shared genes). Thus, the selection of intrinsic genes is robust to gene filtering methods.

In the pancreas study, four batches of gene expression count data were assayed. GSE81076 was profiled using the CEL-seq protocol, and GSE85241 was profiled using the CEL-seq1 protocol. Two remaining batches (GSE86473 and EMATB-5061) were profiled using the SMART-seq2

protocol. After removing low-quality cells and genes, we normalized the raw count data through the deconvolution methods via R package *scater* [5]. Four batches were obtained, containing 7,676, 10,312, 11,383 and 12,486 genes, respectively. If we had retained only genes with the 10% highest mean expression levels, no common gene would have been shared by the four batches. Instead, we also selected the genes with the 50% highest mean expression levels in terms of log-normalized values. As a result, 408 common TEGs were retained.

Similar to the pancreas study, we compared the cell labeling results of two gene filtering methods (**Supplementary Tab. 18**). Once again, the diagonal elements dominate each row. The ARI between the cell labeling of TEGs and FACS labeling is 0.437, whereas the ARI between the cell labeling of HVGs and FACS labeling is 0.608.

In the pancreas study, no gene is shared between the 2,480 HVGs and the 408 TEGs, BUSseq still offers consistent cell clustering in this extreme case. Therefore, BUSseq is robust to the gene filtering strategy.

Supplementary References

[1] Da Wei Huang, Brad T Sherman, and Richard A Lempicki. Systematic and integrative analysis of large gene lists using DAVID bioinformatics resources. *Nature Protocols*, 4(1): 44, 2009.

[2] W Keith Hastings. Monte Carlo sampling methods using Markov chains and their applications. *Biometrika*, 57(1):97–109, 1970.

[3] Gautam Altekar, Sandhya Dwarkadas, John P Huelsenbeck, and Fredrik Ronquist. Parallel metropolis coupled Markov chain Monte Carlo for Bayesian phylogenetic inference. *Bioinformatics*, 20(3):407–415, 2004.

[4] Henry Teicher. Identifiability of mixtures of product measures. *The Annals of Mathematical Statistics*, 38(4):1300–1302, 1967.

[5] Aaron TL Lun, Karsten Bach, and John C Marioni. Pooling across cells to normalize single-cell RNA sequencing data with many zero counts. *Genome Biology*, 17(1):75, 2016.

[6] Michael I Love, Wolfgang Huber, and Simon Anders. Moderated estimation of fold change and dispersion for RNA-seq data with deseq2. *Genome Biology*, 15(12):550, 2014.

[7] Mark D Robinson and Alicia Oshlack. A scaling normalization method for differential expression analysis of RNA-seq data. *Genome Biology*, 11(3):R25, 2010.

[8] Tallulah S Andrews and Martin Hemberg. False signals induced by single-cell imputation. *F1000Research*, 7, 2018.

[9] Mo Huang, Jingshu Wang, Eduardo Torre, Hannah Dueck, Sydney Shaffer, Roberto Bonasio, John I Murray, Arjun Raj, Mingyao Li, and Nancy R Zhang. Saver: gene expression recovery for single-cell rna sequencing. *Nature Methods*, 15(7):539, 2018.

[10] Wuming Gong, Il-Youp Kwak, Pruthvi Pota, Naoko Koyano-Nakagawa, and Daniel J Garry. Drimpute: imputing dropout events in single cell RNA sequencing data. *BMC Bioinformatics*, 19(1):220, 2018.

[11] Wei Vivian Li and Jingyi Jessica Li. An accurate and robust imputation method scImpute for single-cell RNA-seq data. *Nature Communications*, 9(1):1–9, 2018.

[12] Gökcen Eraslan, Lukas M Simon, Maria Mircea, Nikola S Mueller, and Fabian J Theis. Single-cell RNA-seq denoising using a deep count autoencoder. *Nature Communications*, 10(1):1–14, 2019.

[13] David Van Dijk, Roshan Sharma, Juozas Nainys, Kristina Yim, Pooja Kathail, Ambrose J Carr, Cassandra Burdziak, Kevin R Moon, Christine L Chaffer, Diwakar Pattabiraman,

et al. Recovering gene interactions from single-cell data using data diffusion. *Cell*, 174(3): 716–729, 2018.

[14] Florian Wagner, Yun Yan, and Itai Yanai. K-nearest neighbor smoothing for high-throughput single-cell RNA-Seq data. *BioRxiv*, 2018. doi: 10.1101/217737. URL `https://www.biorxiv.org/content/early/2018/04/09/217737`.

[15] Philip Brennecke, Simon Anders, Jong Kyoung Kim, Aleksandra A Kołodziejczyk, Xiuwei Zhang, Valentina Proserpio, Bianka Baying, Vladimir Benes, Sarah A Teichmann, John C Marioni, et al. Accounting for technical noise in single-cell RNA-seq experiments. *Nature Methods*, 10(11):1093, 2013.

[16] Aaron TL Lun, Davis J McCarthy, and John C Marioni. A step-by-step workflow for low-level analysis of single-cell RNA-seq data with Bioconductor. *F1000Research*, 5, 2016.

[17] Luca Scrucca, Michael Fop, Thomas Brendan Murphy, and Adrian E. Raftery. mclust 5: clustering, classification and density estimation using Gaussian finite mixture models. *The R Journal*, 8(1):205–233, 2016. URL `https://journal.r-project.org/archive/2016-1/scrucca-fop-murphy-etal.pdf`.

[18] Leonard Kaufman and Peter J Rousseeuw. *Finding Groups in Data: an introduction to cluster analysis*. John Wiley & Sons, 2009.

[19] Laleh Haghverdi, Aaron TL Lun, Michael D Morgan, and John C Marioni. Batch effects in single-cell RNA-sequencing data are corrected by matching mutual nearest neighbors. *Nature Biotechnology*, 36(5):421, 2018.

[20] Luke Zappia, Belinda Phipson, and Alicia Oshlack. Splatter: simulation of single-cell RNA sequencing data. *Genome Biology*, 18(1):174, 2017.

[21] Joshua D Welch, Velina Kozareva, Ashley Ferreira, Charles Vanderburg, Carly Martin, and Evan Z Macosko. Single-cell multi-omic integration compares and contrasts features of brain cell identity. *Cell*, 177(7):1873–1887.e17, 2019.

[22] Brian Hie, Bryan Bryson, and Bonnie Berger. Efficient integration of heterogeneous single-cell transcriptomes using Scanorama. *Nature Biotechnology*, 37(6):685, 2019.

[23] Romain Lopez, Jeffrey Regier, Michael B Cole, Michael I Jordan, and Nir Yosef. Deep generative modeling for single-cell transcriptomics. *Nature Methods*, 15(12):1053, 2018.

[24] Tim Stuart, Andrew Butler, Paul Hoffman, Christoph Hafemeister, Efthymia Papalexi, William M Mauck III, Yuhan Hao, Marlon Stoeckius, Peter Smibert, and Rahul Satija. Comprehensive integration of single-cell data. *Cell*, 177(7):1888–1902.e21, 2019.

[25] Davide Risso, Fanny Perraudeau, Svetlana Gribkova, Sandrine Dudoit, and Jean-Philippe

Vert. A general and flexible method for signal extraction from single-cell RNA-seq data. *Nature Communications*, 9(1):284, 2018.

[26] Alan P Reynolds, Graeme Richards, Beatriz de la Iglesia, and Victor J Rayward-Smith. Clustering rules: a comparison of partitioning and hierarchical clustering algorithms. *Journal of Mathematical Modelling and Algorithms*, 5(4):475–504, 2006.

[27] Allen W Zhang, Ciara OFlanagan, Elizabeth A Chavez, Jamie LP Lim, Nicholas Ceglia, Andrew McPherson, Matt Wiens, Pascale Walters, Tim Chan, Brittany Hewitson, et al. Probabilistic cell-type assignment of single-cell RNA-seq for tumor microenvironment profiling. *Nature Methods*, 16(10):1007–1015, 2019.

[28] Aliaksei Z Holik, Charity W Law, Ruijie Liu, Zeya Wang, Wenyi Wang, Jaeil Ahn, Marie-Liesse Asselin-Labat, Gordon K Smyth, and Matthew E Ritchie. RNA-seq mixology: designing realistic control experiments to compare protocols and analysis methods. *Nucleic Acids Research*, 45(5):e30–e30, 2017.

[29] Andrew Gelman, Xiao-Li Meng, and Hal Stern. Posterior predictive assessment of model fitness via realized discrepancies. *Statistica Sinica*, pages 733–760, 1996.

[30] Trevor Hastie and Werner Stuetzle. Principal curves. *Journal of the American Statistical Association*, 84(406):502–516, 1989.

[31] Andrew Gelman, Gareth O Roberts, Walter R Gilks, et al. Efficient metropolis jumping rules. *Bayesian Statistics*, 5(599-608):42, 1996.

[32] Gareth O Roberts and Jeffrey S Rosenthal. Examples of adaptive mcmc. *Journal of Computational and Graphical Statistics*, 18(2):349–367, 2009.

[33] Sonia Nestorowa, Fiona K Hamey, Blanca Pijuan Sala, Evangelia Diamanti, Mairi Shepherd, Elisa Laurenti, Nicola K Wilson, David G Kent, and Berthold Göttgens. A single cell resolution map of mouse haematopoietic stem and progenitor cell differentiation. *Blood*, 128(8):e20–31, 2016.

[34] Franziska Paul, Yaara Arkin, Amir Giladi, Diego Adhemar Jaitin, Ephraim Kenigsberg, Hadas Keren-Shaul, Deborah Winter, David Lara-Astiaso, Meital Gury, Assaf Weiner, et al. Transcriptional heterogeneity and lineage commitment in myeloid progenitors. *Cell*, 163(7): 1663–1677, 2015.

[35] Nathan Lawlor, Joshy George, Mohan Bolisetty, Romy Kursawe, Lili Sun, V Sivakamasundari, Ina Kycia, Paul Robson, and Michael L Stitzel. Single-cell transcriptomes identify human islet cell signatures and reveal cell-type-specific expression changes in type 2 diabetes. *Genome Research*, 27(2):208–222, 2017.

[36] Dominic Grün, Mauro J Muraro, Jean-Charles Boisset, Kay Wiebrands, Anna Lyubimova, Gitanjali Dharmadhikari, Maaike van den Born, Johan Van Es, Erik Jansen, Hans Clevers, et al. De novo prediction of stem cell identity using single-cell transcriptome data. *Cell Stem Cell*, 19(2):266–277, 2016.

[37] Luyi Tian, Xueyi Dong, Saskia Freytag, Kim-Anh Lê Cao, Shian Su, Abolfazl JalalAbadi, Daniela Amann-Zalcenstein, Tom S Weber, Azadeh Seidi, Jafar S Jabbari, et al. Benchmarking single cell RNA-sequencing analysis pipelines using mixture control experiments. *Nature Methods*, page 1, 2019.

[38] Steven N Hart, Terry M Therneau, Yuji Zhang, Gregory A Poland, and Jean-Pierre Kocher. Calculating sample size estimates for RNA sequencing data. *Journal of Computational Biology*, 20(12):970–978, 2013.

[39] Travers Ching, Sijia Huang, and Lana X Garmire. Power analysis and sample size estimation for RNA-Seq differential expression. *RNA*, 20(11):1684–1696, 2014.

[40] Hao Wu, Chi Wang, and Zhijin Wu. PROPER: comprehensive power evaluation for differential expression using RNA-seq. *Bioinformatics*, 31(2):233–241, 2015.

[41] Wei Vivian Li and Jingyi Jessica Li. A statistical simulator scDesign for rational scRNA-seq experimental design. *Bioinformatics*, 35(14):i41–i50, 2019.

[42] Angelo Duò, Mark D Robinson, and Charlotte Soneson. A systematic performance evaluation of clustering methods for single-cell RNA-seq data. *F1000Research*, 7, 2018.
