# Supplementary material for: Flexible experimental designs for valid single-cell RNA-sequencing experiments allowing batch effects correction
Source: Nat Commun. 2020 Jul 1;11:3274. doi: 10.1038/s41467-020-16905-2 (PMC7330047; doi:10.1038/s41467-020-16905-2)
Supplement: Supplementary file 3 — Reporting Summary [file 41467_2020_16905_MOESM3_ESM.pdf]

nature research

Corresponding author(s): Yingying Wei

Last updated by author(s): May 15, 2020

Reporting Summary

Nature Research wishes to improve the reproducibility of the work that we publish. This form provides structure for consistency and transparency in reporting. For further information on Nature Research policies, see Authors & Referees and the Editorial Policy Checklist.

Statistics

For all statistical analyses, confirm that the following items are present in the figure legend, table legend, main text, or Methods section.

n/a	Confirmed	
☐	☒	The exact sample size (n) for each experimental group/condition, given as a discrete number and unit of measurement
☒	☐	A statement on whether measurements were taken from distinct samples or whether the same sample was measured repeatedly
☐	☒	The statistical test(s) used AND whether they are one- or two-sided<br>*Only common tests should be described solely by name; describe more complex techniques in the Methods section.*
☐	☒	A description of all covariates tested
☐	☒	A description of any assumptions or corrections, such as tests of normality and adjustment for multiple comparisons
☐	☒	A full description of the statistical parameters including central tendency (e.g. means) or other basic estimates (e.g. regression coefficient) AND variation (e.g. standard deviation) or associated estimates of uncertainty (e.g. confidence intervals)
☐	☒	For null hypothesis testing, the test statistic (e.g. F, t, r) with confidence intervals, effect sizes, degrees of freedom and P value noted<br>*Give P values as exact values whenever suitable.*
☐	☒	For Bayesian analysis, information on the choice of priors and Markov chain Monte Carlo settings
☒	☐	For hierarchical and complex designs, identification of the appropriate level for tests and full reporting of outcomes
☒	☐	Estimates of effect sizes (e.g. Cohen's d, Pearson's r), indicating how they were calculated

Our web collection on statistics for biologists contains articles on many of the points above.

Software and code

Policy information about availability of computer code

Data collection	We conduct data-preprocessing referring to the GitHub repositories https://github.com/MarioniLab/MNN2017 and https://github.com/LuyiTian/sc_mixology.
Data analysis	The C++ source code of BUSseq is available on Github (https://github.com/songfd2018/BUSseq-1.0). All code used for the analysis and generation of the results is deposited at https://github.com/songfd2018/BUSseq-1.1_implementation. Furthermore, we wrap C++ source code as an R package, BUSseq (https://github.com/songfd2018/BUSseq-Rpackage). The CUDA C source code of the GPU version of BUSseq is available at https://github.com/Anguscgm/BUSseq_gpu.

For manuscripts utilizing custom algorithms or software that are central to the research but not yet described in published literature, software must be made available to editors/reviewers. We strongly encourage code deposition in a community repository (e.g. GitHub). See the Nature Research guidelines for submitting code & software for further information.

Data

Policy information about availability of data

All manuscripts must include a data availability statement. This statement should provide the following information, where applicable:

- Accession codes, unique identifiers, or web links for publicly available datasets
- A list of figures that have associated raw data
- A description of any restrictions on data availability

We used published data. GEO accession numbers: GSE72857 and GSE81682 for the hematopoietic study; GSE81076, GSE85241, GSE86473 and the ArrayExpress accession number E-MTAB-5061 for the pancreas study; GSE118767 for the lung adenocarcinoma study.

Field-specific reporting

Please select the one below that is the best fit for your research. If you are not sure, read the appropriate sections before making your selection.

☒ Life sciences ☐ Behavioural & social sciences ☐ Ecological, evolutionary & environmental sciences

For a reference copy of the document with all sections, see nature.com/documents/nr-reporting-summary-flat.pdf

Life sciences study design

All studies must disclose on these points even when the disclosure is negative.

Sample size	All data used in our manuscript are already published data, so the sample sizes are listed in the original publications. Each dataset contains hundreds of cells, and we have listed the exact number in our paper.
Data exclusions	We occasionally excluded cells without FACS sorting labels following Paul et al (2015). For the human pancreas study, we excluded individual pancreatic cells if their sequencing depths are low following Haghverdi et al (2018). Genes whose names can not be matched across different batches were also excluded from our analysis.
Replication	All code for reproducing results and figures in the manuscript is available on GitHub (https://github.com/songfd2018/BUSseq-1.1_implementation).
Randomization	We used all samples in all of the datasets, so there is no randomization issue.
Blinding	We used all samples in all of the datasets, so there is no blinding issue.

Reporting for specific materials, systems and methods

We require information from authors about some types of materials, experimental systems and methods used in many studies. Here, indicate whether each material, system or method listed is relevant to your study. If you are not sure if a list item applies to your research, read the appropriate section before selecting a response.

Materials & experimental systems

n/a	Involved in the study
☒	☐ Antibodies
☒	☐ Eukaryotic cell lines
☒	☐ Palaeontology
☒	☐ Animals and other organisms
☒	☐ Human research participants
☒	☐ Clinical data

Methods

n/a	Involved in the study
☒	☐ ChIP-seq
☒	☐ Flow cytometry
☒	☐ MRI-based neuroimaging
